# Targeting RNA polymerase I to boost natural killer cell anticancer activity in multiple myeloma

**DOI:** 10.1038/s41419-025-08196-6

**Published:** 2025-11-28

**Authors:** Elena Sproviero, Eleonora Gnocchini, Tommaso Cipollone, Sara Petillo, Chiara Cassone, Rosa Molfetta, Alessandra Zingoni, Alessandra Soriani, Cristina Cerboni, Maria Teresa Petrucci, Francesca Fazio, Rossella Paolini, Gabriella Palmieri, Marco Cippitelli

**Affiliations:** 1 Laboratory of Molecular Immunology and Immunopathology - Department of Molecular Medicine, Sapienza University of Rome, Rome, Italy; 2 National Eye Institute, NIH, Bethesda, MD USA; 3 Hematology, Department of Translational and Precision Medicine, Sapienza University of Rome, Rome, Italy; 4 Department of Experimental Medicine, Sapienza University of Rome, Rome, Italy

**Keywords:** Immunoediting, Myeloma

## Abstract

Multiple myeloma (MM) remains an incurable disease despite therapeutic advancements extending survival. Relapses driven by drug resistance and minimal residual disease underscore the need for novel treatment strategies. Natural Killer (NK) cells play a key role in MM immunity, yet their function is suppressed by inhibitory cytokines and metabolites from the tumor microenvironment. Developing anticancer drugs with immunomodulatory properties, such as enhancing tumor sensitivity to NK cell recognition, remains a critical challenge. MM cells exhibit high protein synthesis rates, making them vulnerable to proteostasis disruption. Dysregulated ribosome function and aberrant mRNA translation contribute to proteasome inhibitor resistance. RNA Polymerase I (RNA Pol I)-mediated rDNA transcription, the rate-limiting step in ribosome biogenesis (RiBi), is significantly upregulated in MM. Targeting rDNA transcription and inducing nucleolar stress response (NSR) presents a promising therapeutic approach, though its immunomodulatory role is not well understood. Our study examined two “first-in-cla*ss*” RNA Pol I inhibitors, CX-5461 and BMH-21, which differentially regulate NK cell-activating and inhibitory ligand expression in MM. BMH-21 enhanced NK cell degranulation and increased IFN-γ and TNF-α secretion, demonstrating stronger immunostimulatory effects than CX-5461. Conversely, CX-5461 induced a significant DNA damage response (DDR) and senescence, leading to HLA-E upregulation and suppressing NK cell activity. Mechanistic analyses revealed that HLA-E presentation is governed by ATR/AKT/mTORC1/S6K signaling and Pioneer Round of Translation (PRT), linking its regulation to DDR. This effect was modulated by Lenalidomide and Panobinostat. Moreover, RNA Pol I inhibition enhanced Daratumumab-mediated antibody-dependent cellular cytotoxicity (ADCC) of NK cells against MM, uncovering novel immuno-mediated antitumor mechanisms.

## Introduction

Multiple myeloma (MM) is an incurable hematologic malignancy characterized by the uncontrolled growth of fully developed plasma cells. Its progression and persistence are significantly shaped by the dynamic interactions between innate and adaptive immune responses, which regulate immune defense, impact the ability to recognize and attack malignant cells and play a crucial role in resistance or sensitivity to therapies. A hallmark of MM is the highly immunosuppressive bone marrow (BM) microenvironment, which impairs the functionality of cytotoxic lymphocytes including natural killer (NK) cells [1, 2], innate lymphoid cells equipped with a repertoire of germline-encoded activating and inhibitory receptors that enable them to distinguish between healthy cells and those that are infected, stressed, or transformed [3–6]. NK cells are integral to immunosurveillance against MM onset and progression, mediating both direct cytotoxicity against malignant cells and the secretion of a broad spectrum of cytokines and chemokines that shape the anti-tumor immune response. Therapeutic strategies aimed at enhancing NK cell functionality hold significant promise in MM treatment [2, 7–9]. In this context, chemo-immunotherapy represents a cutting-edge approach to enhance antitumor efficacy, by integrating the direct cytotoxic effects of chemotherapy with the immune-modulating properties of immunotherapy [1, 10–14]. Modulating the balance between activating and inhibitory NK cell signals, thus increasing the susceptibility of MM cells to NK cell-mediated recognition and lysis, may potentiate anti-myeloma immunity, and offer a synergistic approach to improve MM treatment outcome.

Due to their intrinsically high protein synthesis and processing demands, MM cells are particularly vulnerable to proteostasis perturbations. Indeed, pharmacological inhibition of the ubiquitin-proteasome system (UPS) by proteasome inhibitors (PIs), results in the accumulation of misfolded proteins within the endoplasmic reticulum (ER) and cell dysfunction. To maintain high protein synthesis, MM cells exhibit enhanced ribosomal biogenesis (RiBi) driven by increased transcription of ribosomal RNA (rRNA) via RNA Polymerase I (RNA Pol I). This upregulation enhances translational capacity, supporting the elevated production of immunoglobulins and oncogenic proteins essential for MM progression. Hence, therapeutic strategies aimed at disrupting ribosome function, either through direct inhibition of RNA Pol I or by modulating translation initiation, offer novel therapeutic potential, important also in overcoming MM treatment resistance. In this context, integrating ribosome-targeting approaches with existing chemo-immunotherapy regimens could enhance anti-myeloma efficacy by simultaneously disrupting protein synthesis in malignant cells and augmenting immune-mediated cytotoxicity.

This study investigated how modulation of RNA Pol I activity influences the NK cell-mediated immune response against MM and its potential to enhance NK cell-based immunotherapy.

Specifically, we analyzed the mechanisms regulating the expression of activating and inhibitory NK cell ligands on RNA Pol I-inhibited MM tumor cells as a strategy to enhance the cytotoxic functions of NK effector cells. To achieve this, we investigated the immunomodulatory properties of two “first in class” RNA Pol I-targeting agents, both individually and in combination with established therapies, such as IMiDs and the anti-CD38 monoclonal antibody Daratumumab.

Our data indicate that CX-5461 and BMH-21 RNA Pol I inhibitors differently regulate NK cell-mediated killing of MM cells. Mechanistically, although both RNA Pol I inhibitors effectively suppress rRNA synthesis and activate the Nucleolar Stress Response (NSR), their distinct ability to induce DNA damage [15–18] impacts on HLA-E expression levels in MM cells, a ligand for the cognate receptor heterodimer CD94/NKG2A which inhibits NK cell function [19–21], and the CD94/NKG2C, an activating receptor [22, 23]. Recent studies have demonstrated that the peptide repertoire of HLA-E is more diverse than previously understood. Specifically, HLA-E is capable of presenting a broader array of peptides beyond classical major histocompatibility complex (MHC) class I leader peptides containing the VL9 motif [24, 25]. This expanded repertoire includes peptides derived from various cellular proteins, particularly those linked to cellular stress, which influence the inhibitory function of NK cells mediated by CD94/NKG2A [26]. Notably, these peptides can also include proteins associated with cancer metastasis and apoptosis regulation [27], further reinforcing the concept that NK cells engage in the surveillance of cellular stress states. This complexity in self-peptides that stabilize HLA-E suggests an unexpected layer of immunological modulation.

The upregulation of HLA-E by the genotoxic RNA Pol I inhibitor CX-5461 was prevented by ATR kinase inhibition and by RNAi-mediated downregulation of Nuclear Cap Binding Protein Subunit 2 (CBP20/NCBP2) expression, a key regulator of the Pioneer Round of Translation (PRT), a molecular pathway essential for mRNA quality control/surveillance and recently proposed as a source of antigens in the classical HLA-I presentation pathway after genotoxic damage [28, 29]. Notably, the increased expression of HLA-E by CX-5461 was attenuated by co-treatment with Lenalidomide, an immunomodulatory drug, and by Panobinostat, a histone deacetylase inhibitor, both capable of further enhancing NK cell activity against MM cells. Furthermore, both RNA Pol I inhibitors were able to potentiate the action of the therapeutic anti-CD38 monoclonal antibody, Daratumumab [30, 31].

These findings provide novel insights into the immune-mediated antitumor effects of RNA Pol I and RiBi inhibition, as well as NSR activation. They highlight the impact of these mechanisms on natural NK cell effector functions and further clarify the molecular pathways regulating NK cell activity within the immunosuppressive MM microenvironment. Furthermore, they deepen our understanding of how these processes can integrate with existing chemo-immunotherapeutic strategies for MM treatment.

## Results

### Ribosome biogenesis (RiBi) as a therapeutic target in multiple myeloma

**Fig. 1:**
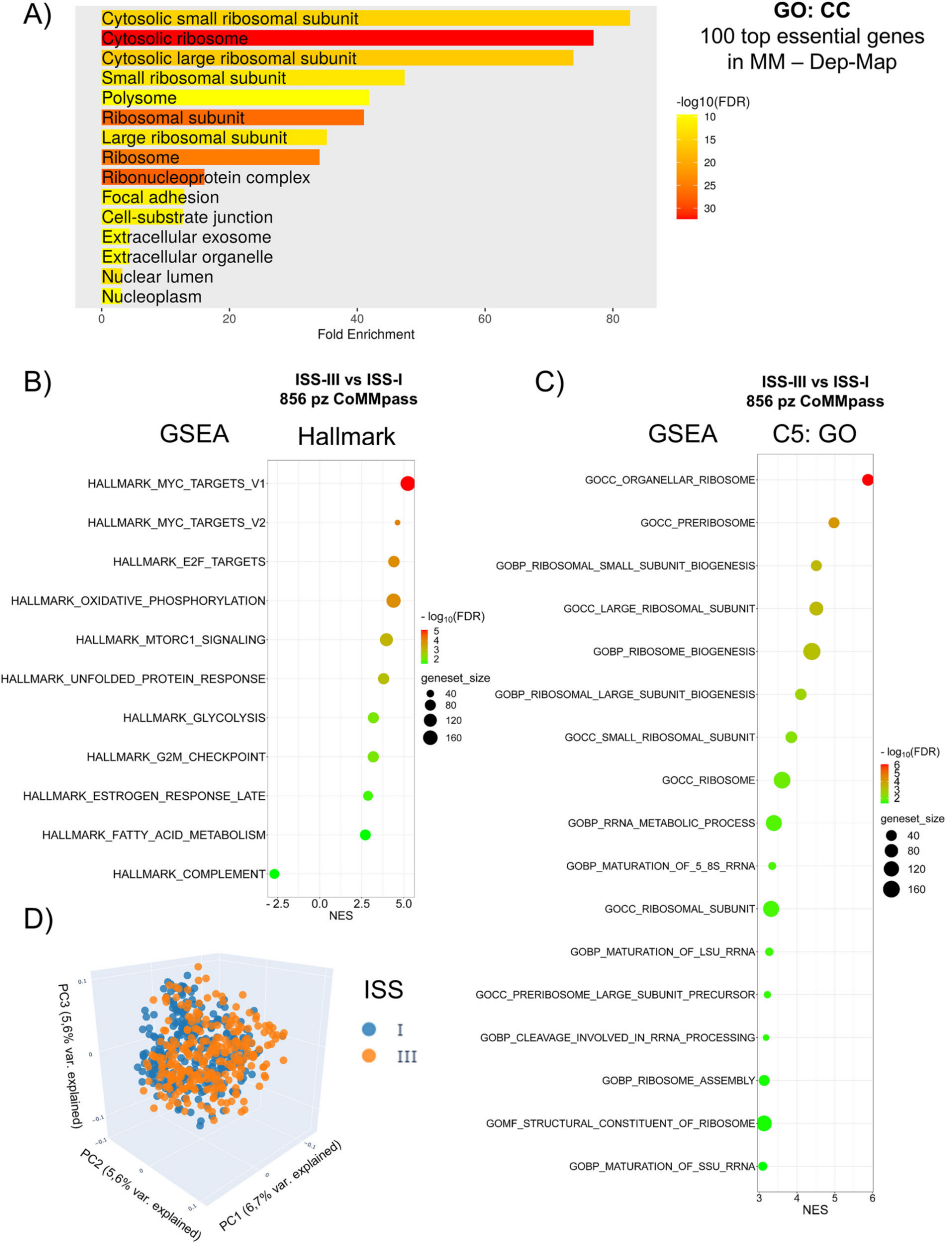
Analysis of molecular pathways related to ribosomal biogenesis and cancer progression in MM. **A** Barplot of the top Gene ontology GO - Cellular Component (CC) pathways enrichment terms for the top 100 most essential genes in MM (RNA-Seq data were extracted from the DepMap repository, *n* = 19 MM cell lines). **B**–**D** RNA-Seq data were extracted from GDC CoMMPass/MMRF database on MM patients with International Staging System (ISS)-III vs ISS-I. GSEA and PCA analysis was performed via Xena “blitzGSEA” web-based bioinformatics analysis platform (https://xenabrowser.net/). **D** PCA plot of MM patients ISS-III vs ISS-I.

To elucidate the role of essential genes in MM progression, we examined CRISPR knockout screening data from the DepMap database (https://depmap.org/portal). Across 19 distinct MM cell lines, the 100 most essential genes were predominantly ribosomal proteins (e.g., RPS6, RPL11, and others, as listed in Supplementary Table I), with significant enrichment in biological processes related to RiBi, translation, and RNA splicing (Fig. 1A). This highlights the critical function of RiBi in MM pathophysiology. Additionally, we analyzed publicly available datasets, including the GDC MMRF-COMMPASS dataset, which contains RNA sequencing (RNA-seq) data from 856 MM patients across different International Staging System (ISS) stages, as well as gene expression profiling arrays from datasets GSE47552 and GSE6477 comparing clonal MM plasma cells (PCs) with normal plasma cells (NPCs). Gene Set Enrichment Analysis (GSEA), performed via the Xena “blitzGSEA” web-based bioinformatics platform, demonstrated that differentially expressed genes between ISS stage III and stage I encompassed canonical tumor progression hallmarks as expected (e.g., MYC targets, E2F targets, and mTORC1) alongside hallmarks related to RiBi and ribosome maturation (Fig. 1B, C). Furthermore, ClueGO-Cytoscape analysis of Gene Ontology - Cellular Component (GO:CC) terms for genes significantly upregulated in MM patients compared to NPCs, based on GEO2R analysis of datasets GSE47552 and GSE6477, revealed substantial enrichment in pathways associated with “ribosomal subunits” and “cytosolic ribosomes” (Supplementary Fig. 1). These findings reinforce previous observations and underscore the therapeutic potential of targeting RiBi in MM, a malignancy characterized by an exceptionally high protein synthesis demand due to the continuous production of large quantities of monoclonal immunoglobulins and free light chains.

### Inhibition of RNA Pol I induces nucleolar stress response in MM cells and regulates NK cell recognition and activity

Ribosome production requires rRNA synthesis, processing, and assembly with ribosomal proteins in the nucleolus, followed by export of pre-ribosomal subunits to the cytoplasm for mRNA translation [32].

Recent studies have demonstrated the antitumor efficacy of RNA Pol I inhibitors (e.g., CX-5461/Pidnarulex) across multiple preclinical models of hematologic and solid tumors [15, 33–41]. Additionally, a phase I clinical trial involving 17 heavily pretreated patients with refractory blood cancers reported stable disease as the best observed outcome with Relapsed Refractory MM (RRMM) [42]. While these findings highlight the therapeutic potential of this strategy, limited data are currently available regarding its possible immunomodulatory effects.

**Fig. 2:**
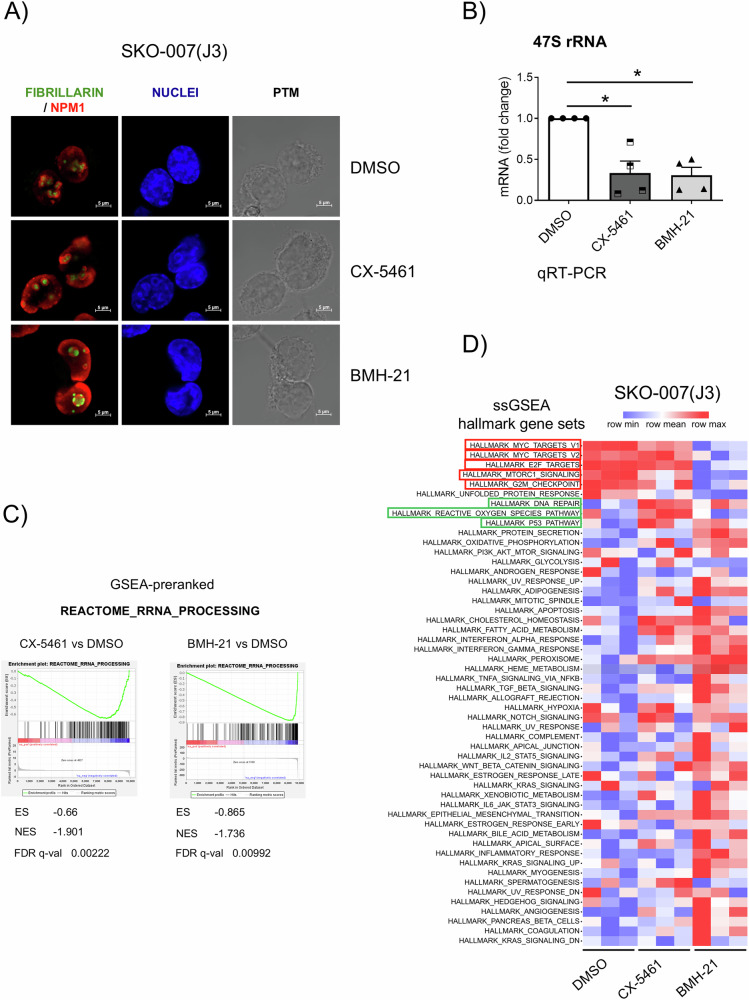
Analysis of RNA Pol I inhibition effects on nucleolar morphology, 47S mRNA levels, and transcriptomic signatures. **A** NPM1 ring formation and Fibrillarin condensation were examined in SKO-007(J3) cells via confocal microscopy. Cells were left untreated or treated with 200 nM CX-5461 or 400 nM BMH-21 for 48 h. Following fixation with paraformaldehyde and permeabilization, cells were stained for NPM1 (red) or Fibrillarin (green), and nuclei were counterstained with DAPI (blue). A representative image is shown. **B** Total mRNA extracted from SKO-007(J3) cells, untreated or treated with the specified RNA Pol I inhibitor for 48 h, was quantified using Real-Time PCR. Data, expressed as fold change values, were normalized to GAPDH and referenced to the untreated sample, which served as the calibrator (*P < 0.05). **C**, **D** Preranked-GSEA and Single Sample GSEA (ssGSEA) were performed on RNA sequencing data from SKO-007(J3) cells, untreated or treated with the specified RNA Pol I inhibitor for 24 h. In (**D**), red boxes indicate relevant downregulated signatures, while green boxes represent upregulated signatures.

**Fig. 3:**
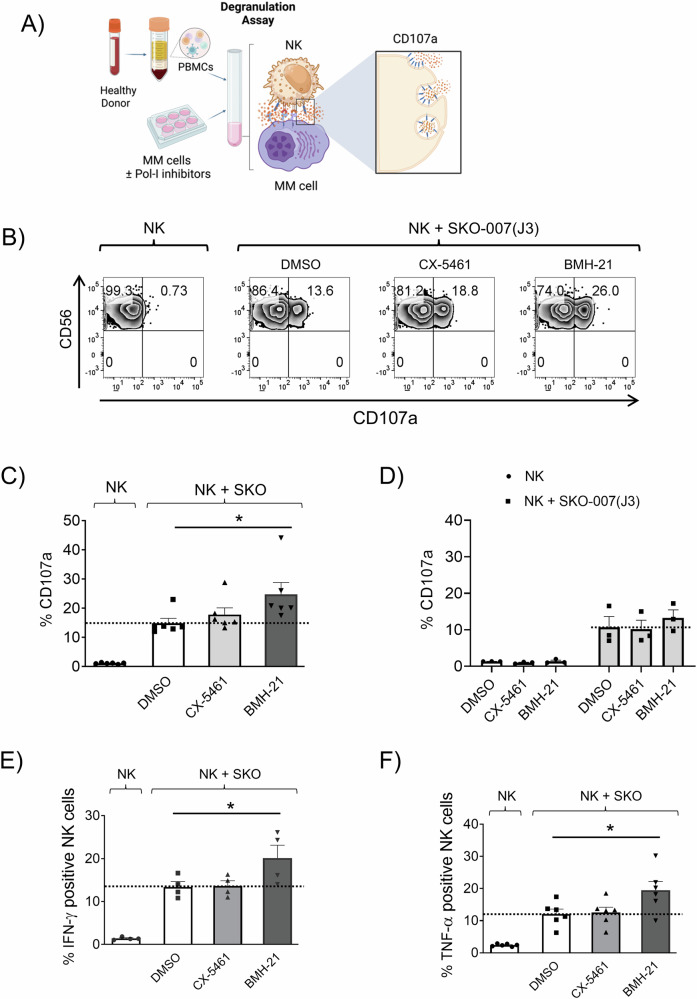
Functional analysis of NK cell degranulation and cytokine expression following RNA Pol I inhibition in MM target cells. **A** Experimental design of the NK cell degranulation assay evaluated via detection of the lysosomal marker CD107a. Peripheral blood mononuclear cells (PBMCs), isolated from healthy donor blood through Ficoll-Hypaque centrifugation, served as effector cells (NK gating strategy is shown in Supplementary Fig. 3). CD107a expression was measured on NK cells gated as CD14-CD19^-^CD45^+^CD3^-^CD138^-^CD56^+^CD16^+^ using a FACS Canto II flow cytometer (BD Biosciences), and the data were analyzed using FlowJo Cytometric Analysis Software (BD Biosciences). **B** A representative experiment is shown. **C** Histograms of the percentage of CD107a-positive NK cells represent the mean value from six independent experiments (*P < 0.05). **D** PBMCs, either untreated or exposed to RNA Pol I inhibitors for 48 h, were co-cultured with untreated SKO-007(J3) target cells. Histograms showing the percentage of CD107a-positive NK cells represent the mean values derived from three independent experiments (*P < 0.05). Panels (**E**) and (**F**) display IFN-γ and TNF-α expression in NK cells (gated from PBMCs) co-cultured with SKO-007(J3) target cells for 6 h. SKO-007-(J3) cells were either untreated or treated with RNA Pol I inhibitors for 48 h. The average percentage across four and six experiments for IFN-γ and TNF-α, respectively, is presented (*P < 0.05). Cytokine expression was evaluated in CD14-CD19^-^CD45^+^CD3^-^CD138^-^CD56^+^CD16^+^ NK cells.

We studied the possible effects of RNA Pol I inhibition on the susceptibility of MM to NK cell-mediated activity. Two “first in class” RNA Pol I inhibitors, CX-5451 and BMH-21 were used, small molecules able to block the transcription of the 47S rRNA genes, a rate-limiting step in ribosome production, thus inducing the NSR [43]. As shown in Fig. 2A, treatment of SKO-007(J3) human MM cells with nanomolar concentrations of CX-5461 or BMH21, induced alterations in nucleolar morphology as evidenced by confocal microscopy with the formation of nucleophosmin (NPM1) rings and condensation of Fibrillarin (NPM), typical features of NSR. To further confirm the effectiveness of the two drugs in this experimental system, treatment with these inhibitors was able to suppress the transcription of the 47S rRNA precursor (Fig. 2B). We then investigated more in detail the molecular pathways regulated by these inhibitors by performing RNA sequencing (RNA-seq) analysis followed by preranked-GSEA and single sample GSEA (ssGSEA). As shown in Fig. 2C, we confirmed the downregulation of genes involved in the regulatory pathway of rRNA processing. Moreover, hallmarks related to gene expression signatures, including c-MYC, E2F, and mTORC1 targets, were significantly inhibited, particularly by BMH-21. In contrast, CX-5461 treatment selectively resulted in the upregulation of gene expression profiles linked to DNA damage, reactive oxygen species (ROS) production, and p53 activation (Fig. 2D). Flow cytometric analyses assessing DNA damage, cellular senescence, and ROS production, key molecular pathways that enhance NK cell recognition of MM cells following genotoxic chemotherapeutic treatment [14, 35–38], demonstrated a preferential induction by treatment with CX-5461 and not BMH-21 in SKO-007(J3) and also in RPMI-8266 MM cells, in accord with previous experimental evidence on the distinct ability of these two inhibitors to induce genotoxic damage [15, 18, 37, 44–48] (Supplementary Fig. 2A–D). Unexpectedly, degranulation assays of NK cells derived from healthy donor PBMCs upon contact with SKO-007(J3) cells, revealed that BMH-21 treatment was more effective in promoting NK cell degranulation (Fig. 3A–C). In this setting, similar results were obtained for the expression of IFN-γ and TNF-α by NK cells (Fig. 3E, F), while no significant alterations in effector/target conjugate formation were observed (data not shown). Direct treatment of PBMCs with Pol I Inhibitors did not alter the basal levels of NK cells degranulation against untreated SKO-007(J3) cells (Fig. 3D), neither significantly affect the membrane expression of several key activating, inhibitory receptors and ligands for death receptors (FAS and TRAIL), except for NKG2D (an activating receptor) and TIGIT (an inhibitory receptor), which were significantly downregulated by BMH-21 inhibitor (Supplementary Fig. 4).

Collectively, these findings suggest that RNA Pol I inhibition, in conjunction with the distinct induction of DNA damage and reactive oxygen species (ROS) production by CX-5461 and BMH-21 in MM cells, differentially influences NK cell functionality. Although both inhibitors effectively induce NSR, they exhibit varying capacities to enhance NK cell-mediated recognition of MM cells.

### RNA Pol I inhibition upregulates NK cell-activating ligands expression on human MM cells

**Fig. 4:**
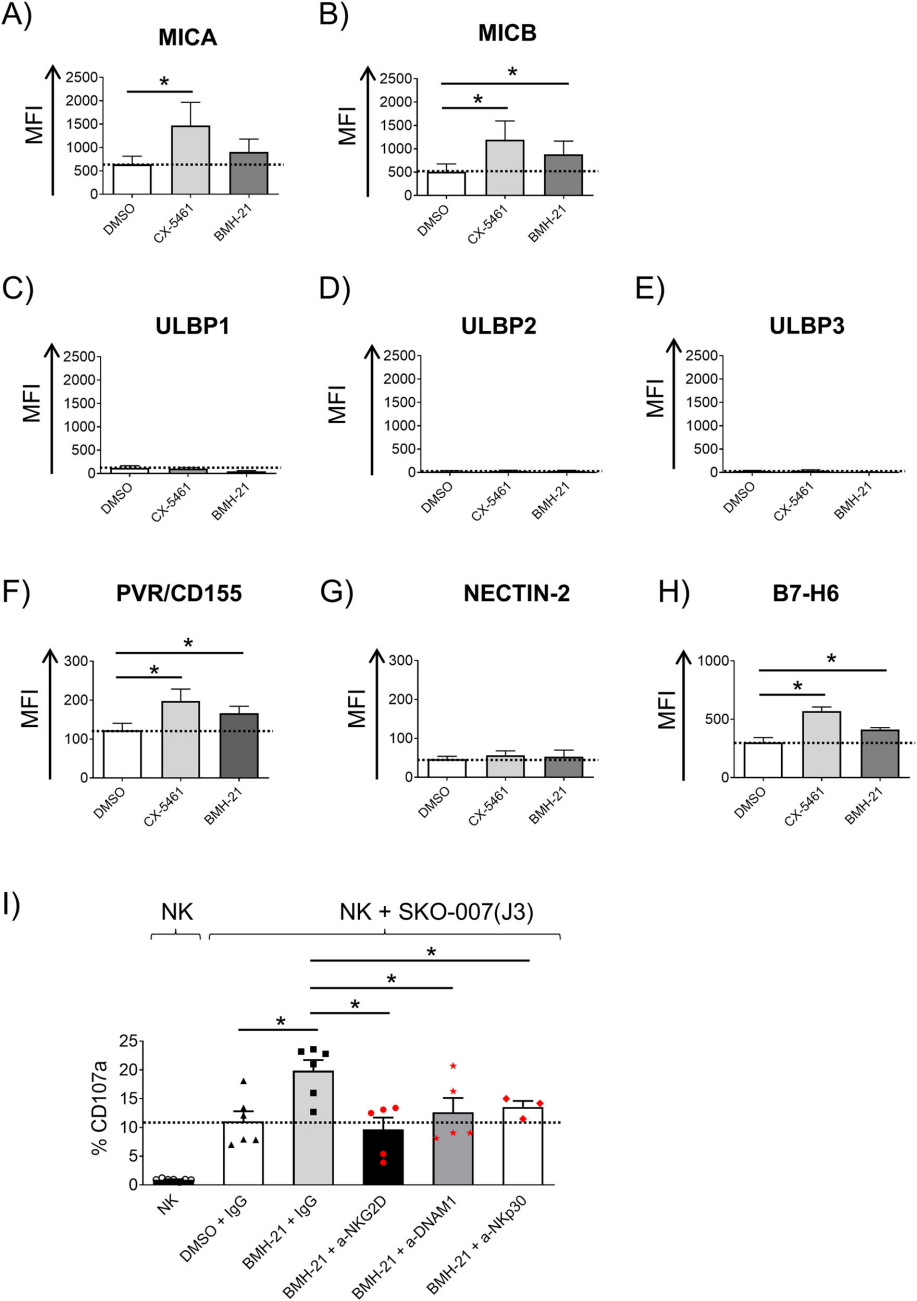
Regulation of NK cell-activating ligands expression by RNA Pol I inhibitors differentially in human MM cells. **A**–**H** Cell surface expression of the specified ligands was assessed on SKO-007(J3) cells treated with CX-5461 or BMH-21 (200 and 400 nM, respectively) for 48 h. Histograms depict the mean fluorescence intensity (MFI) of the specific monoclonal antibody, after subtracting the MFI of the isotype control (*P < 0.05). **I** PBMCs were co-cultured with SKO-007(J3) target cells, either untreated or treated with the indicated inhibitors for 48 h. The assay was conducted at an Effector/Target (E/T) ratio of 2.5:1. To assess the role of NKG2D, DNAM-1 and NKp30 receptors, NK cells pretreated with the specified monoclonal antibodies or IgG control were stimulated with BMH-21-treated target cells. The percentage of CD107a-positive NK cells represents the mean value from at least three independent experiments (*P < 0.05).

Building on these data, we thus investigated the influence of RNA Pol I inhibition on the expression of NK cell regulatory ligands in MM cells. To this end, human MM cell lines already characterized for the expression NKG2D and DNAM-1 ligands [(SKO-007(J3) and RPMI-8226)] [49, 50] were treated with CX-5461 or BMH-21. As shown in Fig. 4A–H, the two inhibitors differentially regulated the cell surface expression of NKG2D ligands (MICA, MICB), PVR/CD155, a ligand for the DNAM-1 receptor, and B7-H6, which binds to the NKp30 receptor. These results were further validated in RPMI-8226 cells (Supplementary Fig. 5), and partially at the mRNA level in the two cell lines, with some differences, for MICA/B, PVR/CD155, and NECTIN-2 (Supplementary Fig. 6). In this context, several experimental studies, including those conducted in our own laboratory (Laboratory of Molecular Immunology and Immunopathology/Laboratory affiliated to Istituto Pasteur Italia-Fondazione Cenci Bolognetti), have demonstrated that the expression of these ligands at the cell surface is regulated at multiple levels and often results from the integrated action of distinct pathways (including transcriptional, post-transcriptional, and post-translational mechanisms). In the present study, the molecular pathways involved in ligand regulation upon induction of the NSR by the two Pol I inhibitors were not investigated. Nevertheless, various mechanisms such as DNA damage induction, ROS production, cellular senescence and p53 activation, among others known to be activated during NSR and to play roles in the regulation of these ligands, may be involved at different stages. Further studies will be required to characterize these regulatory pathways in MM cells, and will be the subject of a dedicated future investigation. Degranulation assays in the presence of blocking antibodies confirmed the involvement of NKG2D, DNAM-1 and NKp30 receptors in NK cell enhanced killing of BMH-1 treated target cells. For these receptor blockade experiments, cells stimulated with BMH-21, displaying higher susceptibility to NK cell lysis, were used (Fig. 4I and Supplementary Fig. 5I). Notably, a more pronounced upregulation on the cell surface was observed with CX-5461 compared to BMH-21, a finding that contradicts the stronger NK cell-activating effect observed with BMH-21. Furthermore, FACS analysis confirmed in part these findings also in freshly isolated bone marrow CD138^+^ MM cells (Supplementary Fig. 7 and Supplementary Table II).

Taken together, these results indicate that RNA Pol I inhibition by CX-5461 and BMH-21 differentially regulates NK cell-activating ligand expression in MM cells and highlight the discrepancy between the upregulation of activating ligands and the effect on NK cell-mediated killing induced by the two inhibitors.

### Differential modulation of HLA-E expression by RNA Pol I inhibitors in MM cells

NK cell activation is regulated by a complex, multifactorial interplay of numerous inhibitory and activating receptors, each characterized by distinct activation thresholds [51]. Activating receptors can interact with “stress-induced ligands” on diseased/stressed cells triggering cell killing; in addition, NK cells express also inhibitory receptors, including killer cell immunoglobulin-like receptors (KIRs) and CD94/NKG2A, which specifically recognize classical (HLA-ABC) and non-classical (HLA-E) major histocompatibility complex (MHC) class I molecules, respectively. This interaction prevents NK cell-mediated lysis of healthy host cells [19, 20, 52]. In the progression of malignant tumors, cancerous cells often upregulate HLA-E expression as a mechanism to circumvent immune surveillance [20, 53–55]. Analysis of HLA-E mRNA expression data from the publicly available MMRF-COMMPASS dataset revealed no significant differences between International Staging System (ISS) stage I and II patients; however, a significant downregulation of HLA-E expression was observed in ISS stage III patients. Comparative analysis using additional publicly available datasets demonstrated that HLA-E expression was elevated in cells from individuals with monoclonal gammopathy of undetermined significance (MGUS), smoldering multiple myeloma (SMM), and multiple myeloma (MM) compared to normal plasma cells (NPCs) (Supplementary Fig. 8), suggesting a potential role for HLA-E in early events of myelomagenesis. In this context, heightened HLA-E expression on malignant plasma cells (PCs) correlates with poor prognosis in MM, particularly in specific patient subgroups, such as those carrying the t(4;14) translocation [56]. In addition, MM cells can also increase the expression of classical HLA class I molecules, leading to reduced sensitivity to NK cells [57].

**Fig. 5:**
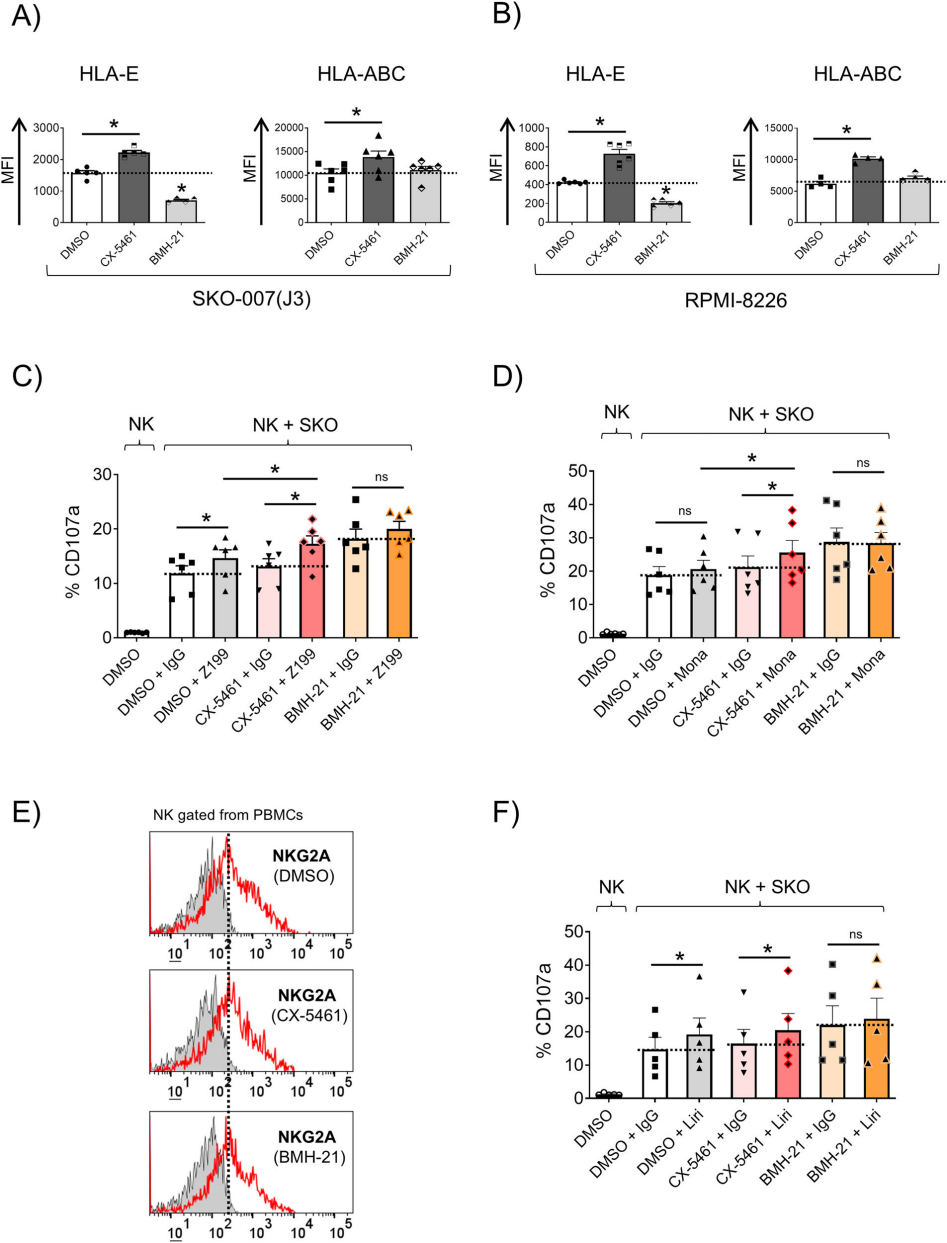
RNA Pol I inhibitors differentially regulate HLA-E and HLA-ABC expression in human MM cell lines. **A**, **B** Cell surface expression of HLA-E and HLA-ABC was assessed by flow cytometry on SKO-007(J3) and RPMI-8226 cells treated with CX-5461 or BMH-21 (200 and 400 nM) for 48 h. Histograms depict the mean fluorescence intensity (MFI) of the indicated HLA, after subtracting the MFI of the isotype control (*P < 0.05). **C**, **D**, **F** Healthy donor PBMCs were co-cultured with SKO-007(J3) target cells, either untreated or treated with the indicated inhibitors for 48 h. NK cell degranulation assay was conducted at an Effector/Target (E/T) ratio of 2.5:1 in complete medium at 37 °C and 5% CO_2_ for 2 h. To assess the role of the NKG2A or KIR receptors, NK cells pretreated with blocking mAbs for NKG2A (clone Z199 or Monalizumab), a blocking mAb for KIRs (Lirilumab/IPH2102) or IgG control were incubated with Pol I inhibitor-treated target cells. The percentage of CD107a-positive NK cells represents the mean value from at least three independent experiments (*P < 0.05). **E** Expression of NKG2A on NK cells gated from PBMCs, as described before, untreated or treated with the indicated RNA Pol I inhibitor for 48 h. Representative histograms are shown.

We investigated the impact of RNA polymerase I (Pol I) inhibitors on the cell surface expression of HLA-E and classical HLA-I in MM cell lines using flow cytometry. Treatment with CX-5461 led to the upregulation of HLA-E and HLA-ABC expression levels, while BMH-21 exhibited a contrasting effect by downregulating HLA-E expression (Fig. 5A, B). Focusing more in detail on the role of HLA-E, anti-NKG2A blocking monoclonal antibodies (clones Z199 and Monalizumab) increased NK cell degranulation against CX-5461 but not BMH-21 treated SKO-007(J3) cells (Fig. 5C, D). Under these experimental conditions, direct exposure of peripheral blood mononuclear cells (PBMCs) from healthy donors to either inhibitor did not result in substantial changes in NKG2A expression levels in NK cells (Fig. 5E). Similarly, an anti-KIR blocking antibody (Lirilumab) selectively upregulated NK cell activation against CX-5461 treated cells, although more moderately (Fig. 5F). These data were consistently validated in CD138^+^ MM cells isolated from the bone marrow of MM patients at distinct stages of disease progression, exhibiting variable basal HLA-E expression (Supplementary Table II), showing that treatment with CX-5461 but not with BMH-21 increased cell surface expression of HLA-E and classical HLA on malignant PCs (Supplementary Fig. 9A, B). Enhanced degranulation of patient-derived NK cells (gated from the CD138⁻ fraction of BM samples) was observed against BMH-21-treated autologous CD38⁺ MM cells (Supplementary Fig. 9D). Moreover, focusing on HLA-E, increased NK cell degranulation against CX-5461-treated MM PCs in the presence of an NKG2A-blocking monoclonal antibody, corroborated the findings obtained with cell lines, in an autologous setting (Supplementary Fig. 9E). In summary, these findings highlight the differential modulation of HLA-E and HLA-ABC expression by CX-5461 and BMH-21 treated MM cells, leading to distinct effects on NK cell killing.

### Dissecting the molecular mechanisms underlying RNA Pol I inhibitor-driven differential regulation of HLA-E expression in MM

**Fig. 6:**
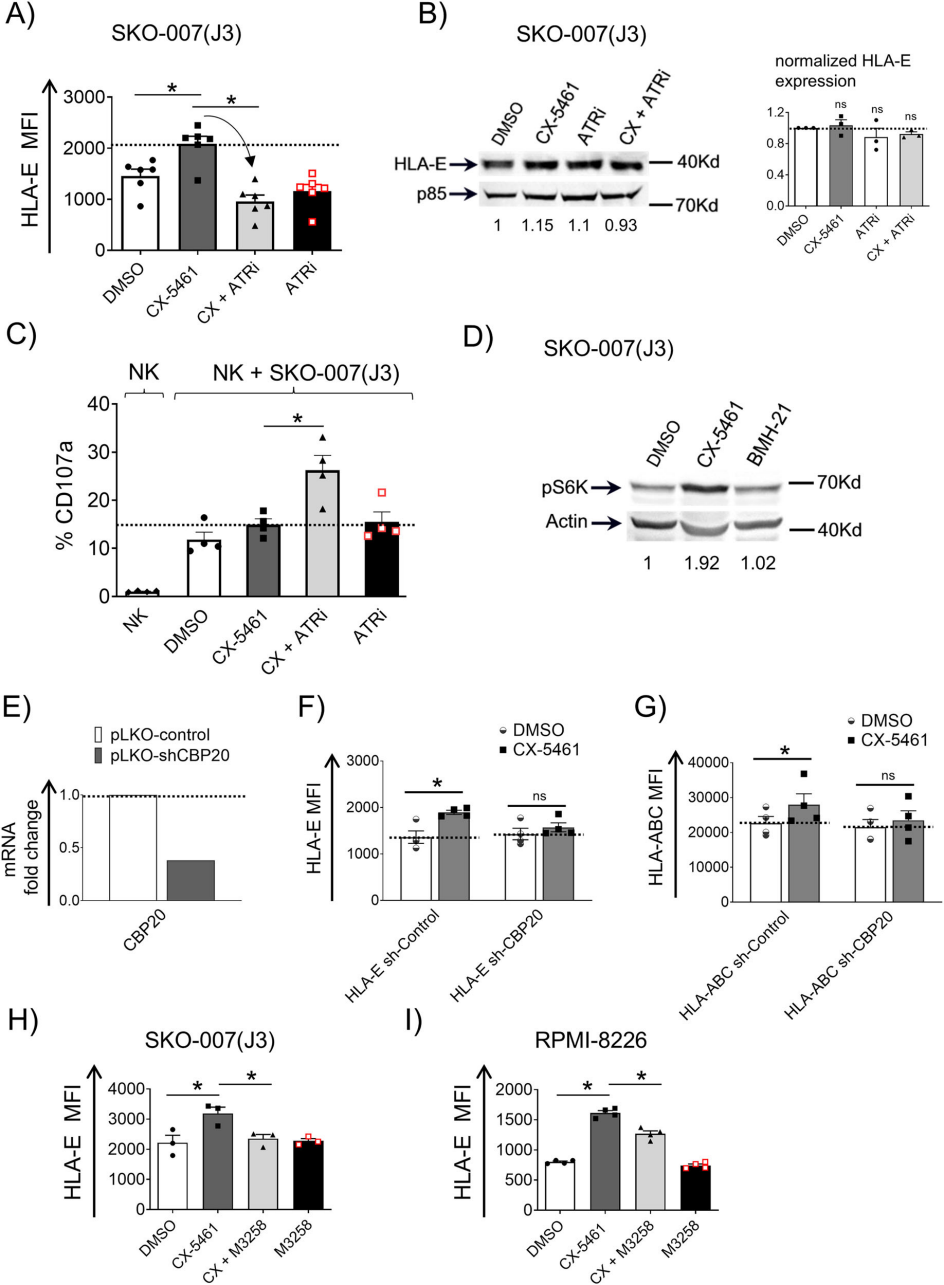
Upregulation of HLA-E by CX-5461: role of DDR and PRT activating pathways. **A** Cell surface expression of HLA-E was assessed by flow cytometry on SKO-007(J3) cells treated with CX-5461in the absence or in the presence of the ATRi (AZD6738/Ceralasertib, 1 µM) for 48 h. Histograms depict the mean fluorescence intensity (MFI) of HLA-E, after subtracting the MFI of the isotype control (*P < 0.05). **B** Western Blot analysis of HLA-E in SKO-007(J3) cells untreated or treated with the indicated inhibitors for 48 h. Expression of p85 was used as protein loading control. A representative Western Blot is shown with densitometric analysis normalized to p85, together with the quantification of three independent experiments (*P < 0.05). **C** NK cell degranulation against CX-treated SKO, in the presence or absence of ATRi was evaluated as the percentage CD107a. PBMCs from healthy donors served as effector cells. Data represent the mean value from four independent experiments (*P < 0.05). **D** Western Blot analysis of pS6K in SKO-007(J3) cells untreated or treated with RNA Pol I inhibitors for 24 h. A representative Western Blot is shown coupled to densitometric analysis, normalized to β-Actin. **E** Total mRNA was obtained from SKO-007(J3) cells stably infected with lentiviruses pLKO-shRNA-CBP20 or pLKO non-targeting shRNA (control) and analyzed for CBP20 mRNA expression by Real-Time qRT-PCR. Data were normalized with GAPDH and referred to the cells infected with non-target shRNA, considered as calibrator. **F**, **G** HLA-E and HLA-ABC expression were analyzed by flow cytometry on SKO-007(J3) pLKO non-target shRNA or pLKO-shRNA-CBP20 infected cells, treated with CX-5461 as described above. The white colored histograms represent basal expression of the indicated HLA, while grey histograms represent the expression after drug treatment. Data are the average of four independent experiments (*P < 0.05). **H**, **I** HLA-E expression was analyzed by flow cytometry on SKO-007(J3) and RPMI-8226 cells treated with CX-5461 in the presence of the LMP7/PSMB8 inhibitor M3258 (300 nM). Data are the average of three independent experiments (*P < 0.05).

Prior research has shown that genotoxic stress induced by ionizing radiation (IR) and chemotherapeutic agents can enhance the expression of classical HLA-I and the presentation of peptides on the cell surface. Nevertheless, the molecular mechanisms through which DNA damage regulates this pathway remain largely unclear. In this scenario, DNA damage can lead to the release of cytosolic nucleic acids, which subsequently activate specific sensors, including cyclic GMP-AMP synthase (cGAS) and the STimulator of Interferon Genes (STING). This activation has been shown to enhance the expression of type I interferons (IFNα/β) which, in turn, promote the transcription of HLA mRNAs [58–61]. In our experimental model, RNA-Seq analysis of SKO-007(J3) cells treated with CX-5461 (able to activate DDR) demonstrated no significant upregulation of genes associated with interferon responses, HLA, or HLA-related pathways (Supplementary Fig. 10A, C, D). As a positive control, IFNα treatment strongly induced the expression of the interferon-responsive gene IFI44 (Supplementary Fig. 10B) and markedly increased HLA-E protein levels, as confirmed by Western Blot analysis in both cell lines (Supplementary Figs. 10E, F), as well as by its expression on the cell membrane (data not shown). Conversely, treatment with RNA Pol I inhibitors did not lead to an increase in HLA-E mRNA or protein levels in MM cell lines (Supplementary Fig. 10G–J). Moreover, the differential effect of CX-5461 remained unchanged following treatment with H-151, a specific STING antagonist, which did not significantly impact the upregulation of HLA-E or HLA-ABC cell surface levels (Supplementary Fig. 11A, B). These findings suggest that the cGAS/STING/IFN pathway is not involved in this model, at least within the treatment timeframe applied in these experiments. Interestingly, recent discoveries have provided insights into the molecular processes underpinning DNA damage-induced HLA-I presentation, emphasizing the involvement of a pathway dependent on polypeptide synthesis through enhanced Pioneer Round of Translation (PRT) [62, 63]. This process, integral to mRNA quality control [64], takes place when the mRNA remains associated with the Cap-Binding Complex (CBP) prior to its transfer to the Eukaryotic Translation Initiation Factor 4E (eIF4E) for “canonical” cytoplasmic translation, and has been proposed as a major contributor to antigen generation in the HLA presentation pathway [28, 29]. Notably, regardless of the nature of DNA damage inducer, the cell type affected and the level of mutational load, the enhanced presentation does not correlate with a general increase in HLA-I protein abundance. Instead, elevated HLA-I expression/presentation arises from enhanced antigen production through a pathway involving the DDR-ATR-AKT-mTORC1-pS6K axis, which upregulates PRT, facilitating the generation of small peptides processed by the proteasome and loaded onto HLA-I molecules [62, 63]. In the context of this novel scenario, high-throughput yeast display techniques for profiling HLA-E ligands interacting with CD94/NKG2x receptors, coupled with computational analyses, have revealed an unexpected complexity in self-peptides able to stabilize HLA-E and modulate NK cell activity. These peptides include proteins linked to cancer metastasis and apoptosis regulation, further extending the concept of surveillance mediated by NK (and potentially T cells) [27]. In addition, also peptides derived from proteins associated with cellular stress states, have been shown to bind to HLA-E, with an effect on the inhibitory activity of NK cells regulated by CD94/NKG2A [26]. Building on these concepts, we hypothesized that the differential effects of the two RNA Pol I inhibitors on HLA-E expression might stem from a shared pathway linked to the regulation of the PRT. This pathway, involving the activation of the DDR, and in combination with the induction of nucleolar stress, could enhance the generation of a distinct repertoire of peptides. These peptides, loaded onto both classical and non-classical HLA-E molecules, may subsequently modulate NK cell activity. Treatment of MM cells with CX-5461 in the presence of a highly specific ATR kinase inhibitor (ATRi-AZD6738/Ceralasertib) resulted in the absence of HLA-E upregulation in both SKO-007(J3) and RPMI-8226 cells. This occurred without significant changes in protein expression, as demonstrated by Western Blot analysis (Fig. 6A, B, Supplementary Fig. 12A, B), thus suggesting a reduced presentation of antigens/peptides. In accord with the effect on HLA-E expression, NK cell degranulation against CX-5461 treated SKO-007(J3) cells was significantly enhanced in the presence of the ATRi, confirming the hypothesis at the functional level (Fig. 6C). In line with previous findings on HLA-ABC presentation after DNA damage [62, 63], the inhibition of AKT and mTORC-1 similarly suppressed HLA-E upregulation in CX-5461-treated cells (Supplementary Fig. 12C–F). Interestingly, CX-5461 enhanced S6K phosphorylation, which promotes PRT initiation [65], while the non-genotoxic inhibitor BMH-21 was unable to replicate this effect (Fig. 6D). In this setting, treatment of MM cells with CX-5461 did not markedly affect the protein expression levels of the kinases ATR and S6K under identical experimental conditions, while a little decrease in the expression level of AKT and S6K was observed in RPMI-8226 cells in the presence of BMH-21 (Supplementary Fig. 13A–F). Notably, lentiviral-mediated shRNA interference targeting the CBP20 gene, an integral component of the PRT initiation complex [66, 67], effectively prevented the upregulation of both HLA-E and HLA-ABC in CX-5461-treated cells, strongly supporting a mechanistic role of PRT in classical and non-classical HLA-I upregulation induced by CX-5461 (Fig. 6E–G); in addition to this, pharmacologic inhibition of the catalytic LMP7/PSMB8 immunoproteasome subunit attenuated upregulation of HLA-E by CX-5461, indicating that the activity of the immunoproteasome is required for increased presentation (Fig. 6H, I). To further validate these observations, we also examined whether key mRNA biogenesis processes, specifically transcription and splicing, are required for enhanced HLA-E presentation induced by CX-5461, as previously described for HLA-ABC after DNA damage [62, 63]. To this end, we assessed HLA-E expression in cells treated with CX-5461 in combination with either the transcription inhibitor Triptolide or the splicing inhibitor Isoginkgetin. Flow cytometry analysis showed that both inhibitors effectively suppressed CX-5461-induced upregulation of HLA-E and HLA-ABC, without significantly altering overall HLA-E and classical HLA-I protein levels. This suggests that post-transcriptional and post-splicing events are crucial for the enhanced HLA-E presentation induced by CX-5461 (Supplementary Fig. 14A, B). Finally, RNA-seq analysis of cells treated with CX-5461 suggests that DNA damage did not significantly alter alternative splicing patterns, at least within the sequencing depth applied in this study. This includes events such as alternative 3’ splice site selection (alt_3prime), alternative 5’ splice site selection (alt_5prime), exon skipping, intron retention, multiple exon skipping, and mutually exclusive exons, with the potential to generate mRNA transcripts encoding premature termination codons (PTCs) proposed as a possible source of neoantigens [68] (Supplementary Fig. 15).

### Regulation of cell cycle progression and HLA-E expression by CX-5461: modulation by Lenalidomide and Panobinostat

**Fig. 7:**
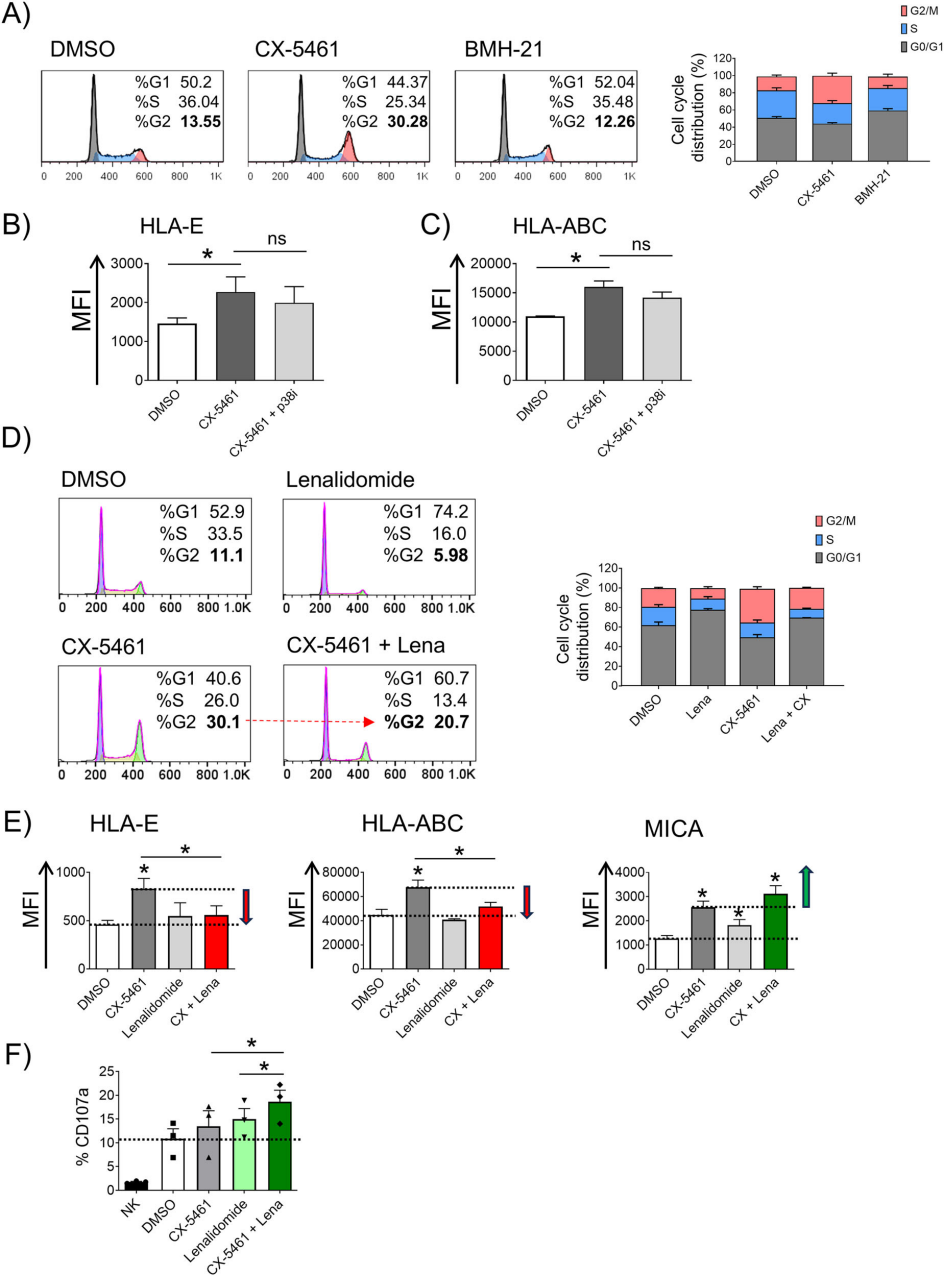
Cell cycle analysis and upregulation of HLA-E by CX-5461: modulation by Lenalidomide. **A** Analysis of the cell cycle in SKO-007(J3) cells following 48 h of treatment with RNA Pol I inhibitors. The figure shows a representative experiment, and the quantification of 5 independent experiments. **B**, **C** Inhibition of p38 kinase (SB203580, 10 µM) does not modify the CX-5461-induced upregulation of HLA-E or HLA-ABC in SKO-007(J3) cells (48 h of treatment) (*P < 0.05). **D** Cell cycle analysis in SKO-007(J3) cells treated with CX-5461 alone or in combination with Lenalidomide (5 µM, 48 h). A representative experiment is shown with the quantification of 3 independent experiments. The red arrow in the representative experiment indicates the decrease in the G2 phase in the presence of Lenalidomide. **E** Flow cytometry was used to evaluate the surface expression of HLA-E, HLA-ABC, and MICA on SKO-007(J3) cells treated with 200 nM CX-5461, either alone or with Lenalidomide (5 µM) for 48 h. Histograms illustrate the mean fluorescence intensity (MFI) of the indicated ligand, adjusted by subtracting the MFI of the isotype control. Red and green arrows signify the downregulation or upregulation, respectively, compared to CX-5461-treated cells. **F** NK cell degranulation was evaluated using the lysosomal marker CD107a as described above. As source of effector cells, PBMCs purified from healthy donor blood were used as described previously. Cells were co-cultured with the SKO-007(J3) untreated or treated with CX-5461 in the absence or in presence of Lenalidomide (48 h). The assay was performed at the Effector/Target (E/T) ratio of 2.5:1 in complete medium at 37 °C and 5% CO_2_ for 2 h. CD107a expression was evaluated on NK cells gated as CD14^-^CD19^-^CD45^+^CD3^-^CD138^-^CD56^+^CD16^+^, using a FACS Canto II flow cytometer (BD Biosciences) and data were analyzed by FlowJo V10 Cytometric Analysis Software (BD Biosciences (*P < 0.05).

Cell proliferation is critically involved in the regulation of downstream signaling pathways following DNA damage, and recent research has demonstrated that enhanced HLA-ABC presentation does not occur in cells arrested in the G0/G1 phase following DNA damage [62, 63]. Furthermore, ATR activation is preferentially observed during the G2 phase rather than the G1 phase post-DNA damage [69]. Notably, the analysis of the cell cycle in SKO-007(J3) cells treated with RNA Pol I inhibitors revealed that CX-5461 significantly enhanced the proportion of cells arrested in the G2 phase, in contrast to BMH-21 (Fig. 7A), in accord with the preferential induction of DNA damage and cellular senescence by this compound (as illustrated in Supplementary Fig. 2). In this regard, the expression of HLA-E has been demonstrated to be regulated by pro-inflammatory cytokines linked to the senescence-associated secretory phenotype (SASP), a mechanism orchestrated through p38 MAP kinase signaling in vitro [70]. Nonetheless, within our experimental model, p38 kinase inhibition did not influence CX-5461-driven upregulation of HLA-E or HLA-ABC (Fig. 7B, C). To better investigate the possible correlation between cell-cycle status and HLA-E expression, we explored the efficacy of IMiDs chemotherapeutic agents (e.g., Lenalidomide) in combination with CX-5461, given their reported capability to induce growth arrest and preferential enrichment in G1 phase of MM cells [71]. Our results indicate that co-treatment of SKO-007(J3) cells with Lenalidomide leads to a reduction in the proportion of cells in the G2 phase (Fig. 7D) while concurrently reducing CX-5461-induced membrane expression of HLA-E (and HLA-ABC) (Fig. 7E). Conversely, this combination further enhanced the expression of the NKG2D ligand MICA, which was independently upregulated by both CX-5461 and Lenalidomide. These results were corroborated by NK cell degranulation assays, which showed a marked enhancement of NK cell activity after co-treatment (Fig. 7F). In this experimental setting, we also assessed HLA-E and MICA expression across distinct phases of the cell cycle. As illustrated in Supplementary Fig. 16A–C, HLA-E expression was preferentially elevated in the G2 phase upon treatment with CX-5461, but its level of expression was reduced in combination with Lenalidomide. Consistently, MICA expression displayed an opposite trend, being higher in the G2 phase under co-treatment, indicating a selective modulation for HLA-E. This regulatory mechanism was further confirmed using the histone deacetylase inhibitor Panobinostat, which was recently demonstrated to exhibit significant synergistic interactions with CX-5461 against MM in both in vitro and in vivo settings [72]. Similar to Lenalidomide, Panobinostat reduced CX-5461-induced G2 phase enrichment and differentially modulated HLA-E and MICA expression (Supplementary Fig. 16D, E). These findings provide deeper insight into the molecular mechanisms underlying HLA-E regulation by RNA Pol I inhibitors and underscore promising combination therapeutic approaches with anti-MM agents aimed at augmenting NK cell-mediated responses against MM.

### Inhibition of RNA Pol I in MM cells increases antibody-dependent degranulation of NK cells

**Fig. 8:**
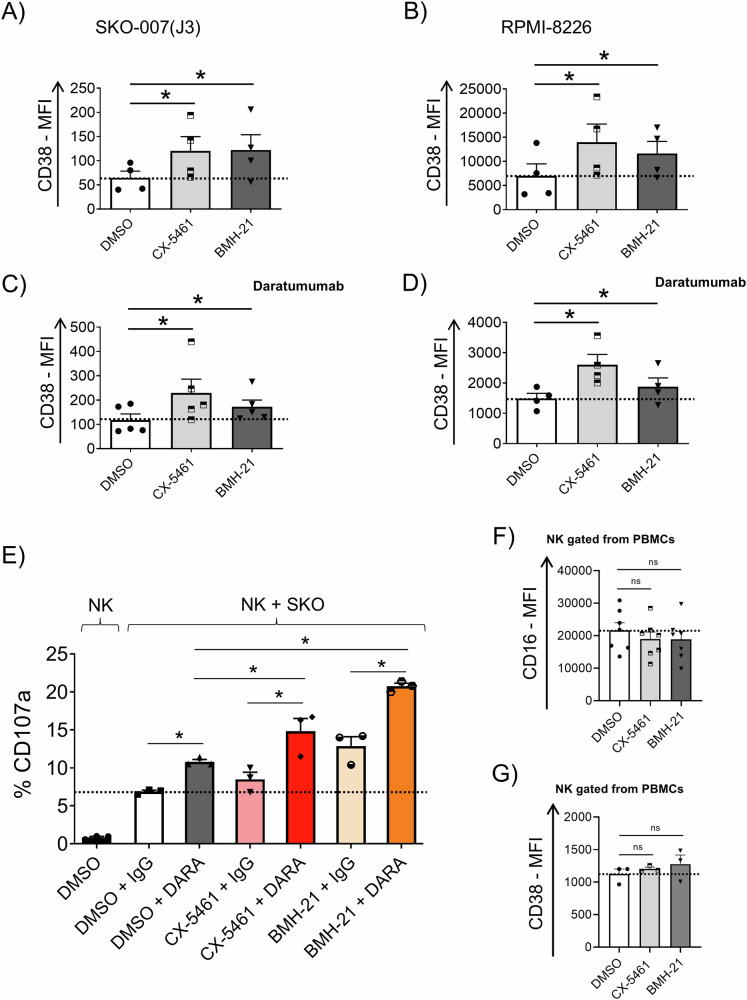
Daratumumab-dependent ADCC against MM cells is increased by RNA Pol I inhibition. **A**, **B** Cell surface expression of CD38 was assessed by flow cytometry on SKO-007(J3) and RPMI-8226 cells treated with CX-5461 or BMH-21 (200 and 400 nM) for 48 h. Histograms depict the mean fluorescence intensity (MFI) of CD38 after subtracting the MFI of the isotype control (*P < 0.05). **C**, **D** Cell surface binding of Daratumumab was evaluated by indirect flow cytometry on SKO-007(J3) and RPMI-8226 cells treated with CX-5461 or BMH-21 (200 and 400 nM) for 48 h. In these assays, Daratumumab was used as an unconjugated primary antibody, followed by staining with a FITC-conjugated anti-human secondary antibody. Histograms display the mean fluorescence intensity (MFI) after subtraction of the isotype control MFI (*P < 0.05). **E** Healthy donor PBMCs were incubated with SKO-007(J3) cells untreated or treated with CX-5461 or BMH-21 as described above. In this setting, anti-CD38/Daratumumab or isotype-control (0.5 µg/10^6^) were added to target cells for 15 min at room temperature and then washed twice in complete medium. CD107a expression was evaluated on gated NK cells from PBMCs. Average percentage of CD107a positive cells was calculated based on three independent experiments (*P < 0.05). **F** Expression of CD16 and **G** CD38 on NK cells gated from PBMCs, untreated or treated with the indicated RNA Pol I inhibitor for 48 h. Histograms represent the average of at least three independent experiments (*P < 0.05).

Antibody-Dependent Cellular Cytotoxicity (ADCC) is a powerful cytolytic mechanism driven by NK cells recognizing IgG-coated targets by means of CD16 low-affinity FcγRIIIA activating receptor; it constitutes a fundamental element in the clinical effectiveness of numerous therapeutic monoclonal antibodies. Consequently, augmenting ADCC remains a critical goal in the field of onco-immunology. Daratumumab, a therapeutic anti-CD38 monoclonal antibody approved for treatment of MM, facilitates Complement-Dependent Cytotoxicity, Antibody-Dependent Cellular Phagocytosis, and ADCC against CD38^+^ MM cells [73, 74]. We examined whether RNA Pol I inhibitors could regulate CD38 expression in MM cell lines. As shown in Fig. 8A, B, both inhibitors were effective in enhancing the membrane expression of CD38 in the cell lines used as models in this study. This increase was observed in the SKO-007(J3) cell line, characterized by low baseline CD38 expression, as well as in the RPMI-8226 cell line, which exhibits high baseline CD38 expression. These results were further confirmed by directly employing Daratumumab as an unconjugated primary antibody, followed by indirect immunostaining with a FITC-conjugated anti-human secondary antibody under identical experimental conditions (Fig. 8C, D). Based on these findings, we used the SKO-007(J3) cell line, which offers a more suitable model for assessing the advantage of Daratumumab-driven ADCC due to its lower CD38 expression, to perform NK cell degranulation assays with various treatment combinations. As illustrated in Fig. 8E, co-treatment with either inhibitor enhanced Daratumumab efficacy, with the most pronounced effect observed with BMH-21, as expected. Notably, neither inhibitor affected the basal expression of the CD16 receptor nor CD38 in NK cells (Fig. 8F, G). In a distinct set of experiments, the combination of CX-5461 + Lenalidomide + Daratumumab was also analyzed. The data indicate that the triple treatment does not significantly enhance the effect of either Daratumumab + CX-5461 or Daratumumab + Lenalidomide (Supplementary Fig. 17).

Collectively, these findings underscore an additional therapeutic and immunomodulatory function of RNA Pol I inhibitors in MM, highlighting its promise for boosting NK cell-mediated immune responses in a perspective of combinatory chemo-immunotherapeutic strategy.

## Discussion

This study explored the immunomodulatory effects of Pol I inhibitors on NK cell activity to support the therapeutic potential of targeting RiBi in MM. Analysis of CRISPR knockout screening data from the DepMap database demonstrated a significant enrichment of pathways associated with ribosomal proteins, highlighting the critical role of RiBi in MM pathogenesis. Furthermore, GSEA analysis across publicly available MM datasets reinforced these findings, showing that genes differentially expressed in advanced MM stages are enriched in biological processes related to ribosome biogenesis, translation, and RNA splicing (Fig. 1 and Supplementary Fig. 1). These findings align with the increased demand for ribosome production and protein synthesis, driven by the rapid proliferation of cancer cells. This is particularly evident in MM, which is marked by intense antibody synthesis and production, highlighting RiBi as a promising target for therapeutic advancement. Thus, therapeutic strategies able to affect ribosome function, either through direct inhibition of RNA Pol I or by modulating translation initiation, can offer novel therapeutic potential. Both RNA Pol I inhibitors CX-5461 and BMH-21 induced NSR in our MM cell model, as evidenced by the formation of NPM1 rings, fibrillarin condensation, and the suppression of 47S rRNA precursor transcription. However, the inhibitors displayed distinct molecular profiles. RNA-seq analysis revealed that BMH-21 primarily downregulated genes involved in rRNA processing, c-MYC, E2F, mTORC1 targets, whereas CX-5461, in addition to inhibiting genes involved in rRNA processing also induced genes related to DNA damage, ROS production, and p53 activation (Fig. 2). Surprisingly, BMH-21 treated MM cells more effectively induced NK cell degranulation and cytokine production, despite not inducing significant DNA damage, ROS production or senescence as observed for CX-5461, molecular pathways described to upregulate the expression of NK cell-activating ligands in MM after genotoxic stress [75–77] (Fig. 3 and Supplementary Fig. 2). Indeed, CX-5461, more effectively than BMH-21, enhanced the expression of NK cell-activating ligands (MICA, MICB, PVR/CD155, NECTIN-2, and B7-H6) on MM cell lines, and this upregulation was partially corroborated in patient-derived CD138^+^ MM cells (Fig. 4 and Supplementary Figs. 5 and 7). As NK cell activation is under the control of opposite activating and inhibitory signals provided by target cells, we turned our attention on examining the possible impact of Pol I inhibitors on the expression of classical and non-classical HLA-I molecules, which represent ligands for inhibitory KIR and CD94/NKG2A receptors, respectively, as an event that could explain the differential ability of CX-5461 and BMH-21 to enhance NK cell activation against MM. Our data show that CX-5461 consistently upregulated HLA-E and HLA-ABC expression levels, while BMH-21 exhibited a contrasting effect by downregulating HLA-E expression in MM cell lines. These data were further confirmed in primary CD138⁺ MM cells isolated from patients at distinct stages of disease progression, exhibiting variable basal HLA-E expression. Treatment with CX-5461, but not with BMH-21, led to an upregulation of cell surface HLA-E and classical HLA molecules. In addition, blocking NKG2A, the inhibitory receptor for HLA-E, significantly enhanced NK cell degranulation in CX-5461-treated cells, while blocking KIR receptors upregulated NK cell activation against CX-5461 treated cells more moderately (Fig. 5D, F and Supplementary Fig. 9E). These findings may help explain the apparent paradox of reduced NK cell degranulation against CX-5461-treated target cells, despite the latter demonstrating a more pronounced upregulation of NK cell-activating ligands compared to BMH-21-treated cells.

To dissect the molecular mechanisms driving the differential regulation of HLA-E induced by these two inhibitors, we investigated the possible roles of the DDR and the PRT pathways. The results indicate that CX-5461-mediated HLA-E upregulation is regulated by a ATR-AKT-mTORC1-pS6K axis, already shown to enhance PRT activity and classical HLA-I presentation after genotoxic damage [62, 63]. Indeed, pharmacologic inhibition of ATR, AKT, mTORC1 or downregulation of CBP20 expression (a factor initiating PRT [64]) significantly abrogated HLA-E upregulation in MM cells treated with CX-5461 (Fig. 6 and Supplementary Fig. 12). In addition, we also investigated the interplay between cell cycle progression and HLA-E expression, as recent research has shown that classical HLA presentation is regulated by the proliferative state of cells, being higher in the G2 phase of the cell cycle post-DNA damage, where ATR activation is preferentially observed [69]. Unlike BMH-21, CX-5461 triggered the activation of DDR and increased G2 phase arrest (Supplementary Fig. 2 and Fig. 7D), which correlated with enhanced HLA-E expression. This observation suggests that HLA-E presentation can be regulated through a similar mechanism. In contrast, Lenalidomide and Panobinostat, which preferentially increase G1-phase arrest in these cells [71, 72], mitigated CX-5461-induced G2-phase arrest and HLA-E upregulation, thereby enhancing NK cell activity (Fig. 7 and Supplementary Fig. 16). Notably, under identical experimental conditions, the expression of the NK cell-activating ligand MICA was markedly upregulated, suggesting an intriguing potential for combinatorial therapeutic approaches in conjunction with other anti-MM agents. Furthermore, both RNA Pol I inhibitors induced an increase in CD38 expression on MM cells, thereby potentiating Daratumumab-mediated ADCC, with BMH-21 demonstrating the most pronounced effect (Fig. 8). These findings suggest that administration of IMiDs or Panobinostat prior to CX-5461 could more effectively attenuate CX-5461 induced accumulation of cells in the G2 phase, during which HLA-E expression is more elevated. Further preclinical investigations are warranted to better elucidate the regulatory interplay between Pol I inhibitors, IMiDs, and/or Daratumumab, with the objective of optimizing treatment regimens and scheduling.

**Fig. 9:**
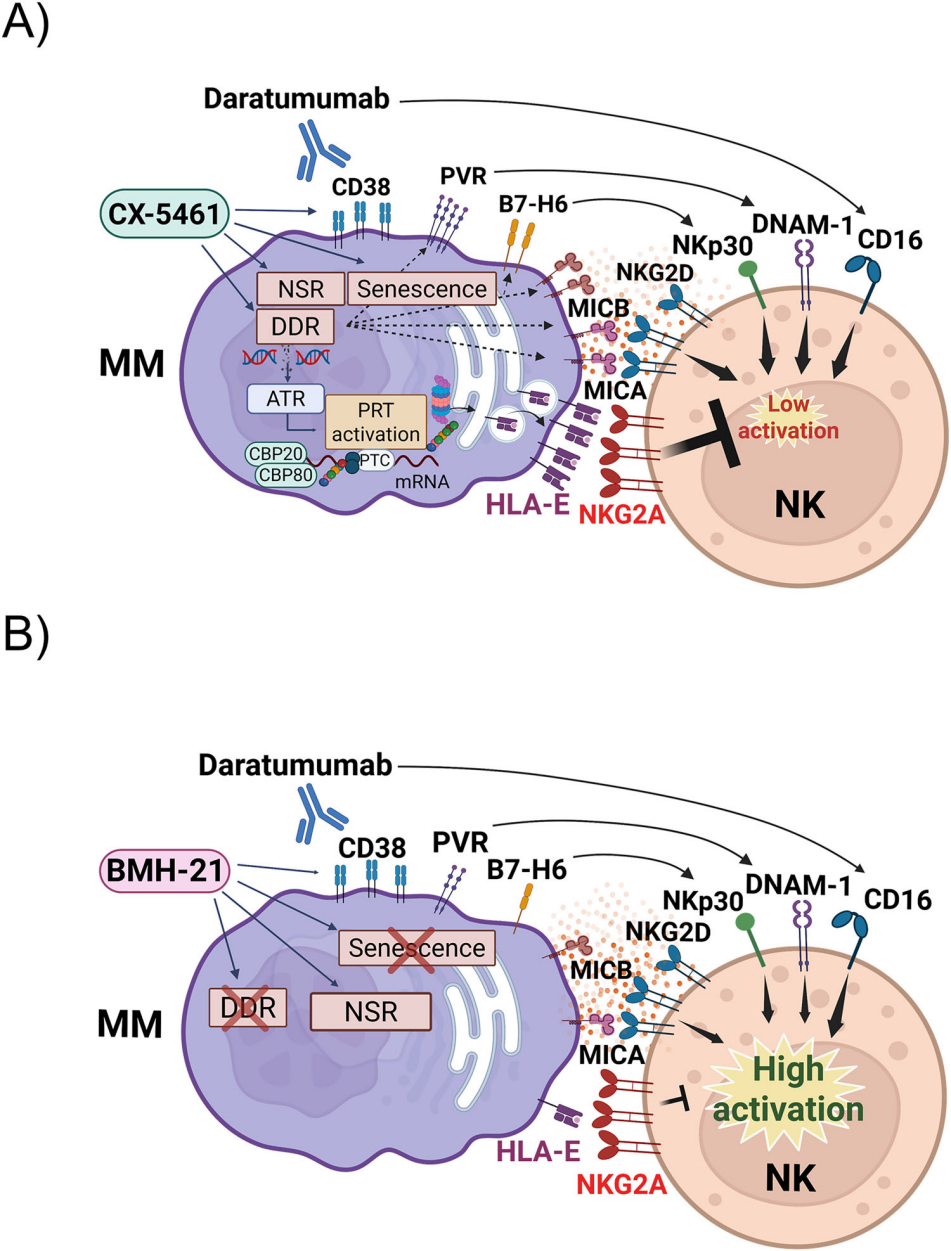
Final model: RNA Pol I inhibitors differentially modulate NK cell-mediated anticancer activity in MM. RNA Polymerase I inhibitors, CX-5461 (**A**) and BMH-21 (**B**), distinctly regulate the expression of NK cell-activating and inhibitory ligands in MM cells. CX-5461 induces DDR and promotes cellular senescence, leading to a significant upregulation of NK cell-activating ligands. However, this effect is accompanied by an increased expression of HLA-E, an inhibitory NK cell ligand, which attenuates NK cell degranulation against MM cells. In contrast, BMH-21 induces a moderate activation of NK cell-activating ligands without upregulating HLA-E, thereby facilitating more effective NK cell degranulation compared to CX-5461. Mechanistic analyses suggest that HLA-E expression is governed by ATR/AKT/mTORC1/S6K signaling and PRT, linking its regulation to DDR activation. (Figure Created with BioRender.com with granted permission and license).

In summary, this study confirms the role of RiBi as a therapeutic target in MM and highlights the distinct immunomodulatory effects of RNA Pol I inhibitors with differential capacities to induce DNA damage, particularly in shaping NK cell-mediated anti-MM responses (Fig. 9). Notably, these findings align with recent research on macrophages in breast cancer, underscoring RiBi’s essential role in remodeling the tumor immune microenvironment. Indeed, the RNA Pol I inhibitor BMH-21 effectively reprogrammed macrophages from a tumor-promoting to a tumor-suppressing state, fostering an inflammatory microenvironment through nucleolar stress [78]. In this scenario, the distinctive molecular characteristics of RNA polymerase I inhibitors, including their capacity to modulate HLA-E presentation, enhance the expression of NK cell-activating ligands, and influence macrophage polarization, present promising avenues for the development of innovative cancer therapies. In a broader context, these findings merit further investigation and expansion in future studies, as the application of these inhibitors and the modulation of the NSR may also affect additional immune cell subsets. This has been exemplified in a colorectal cancer (CRC) immunotherapy model, where treatment with CX-5461, either alone or in combination with anti-PD-1 antibodies, resulted in higher levels of CD3⁺, CD4⁺, and activated cytotoxic T lymphocytes (CTLs), alongside a reduction in myeloid-derived suppressor cells (MDSCs) within the murine spleen [79]. These observations were further substantiated by a recent study that examined the single-cell tumor landscape in preclinical models (CRC - MC38 and CT26) treated with CX-5461. The analysis revealed that CX-5461 administration led to increased levels of cytotoxic granules secreted by CD8⁺ T cells and NK cells and to a decrease of regulatory T cells (Tregs) and MRC1^+^ (immunosuppressive-like) TAMs [80]. Notably, and consistent with the above mentioned observations in a breast cancer model treated with BMH-21, CX-5461 also promoted pro-inflammatory reprogramming of macrophages [80], confirming the pivotal role of RiBi in regulating immunosuppressive non-malignant cell populations within the tumor microenvironment. Moreover, alterations in the repertoire of peptides presented by MHC class I molecules may also occur in the presence of the genotoxic CX-5461. Finally, CX-5461 has recently been shown to modulate regulatory T cell function via the p53-DUSP5 signaling axis in a skin allograft model [81], raising the possibility of a similar regulatory mechanism operating within the tumor microenvironment.

While this study focused on the regulatory effect of RNA Pol I inhibitors on HLA-E/NKG2A regulatory activity within the context of MM and NK cell responses, further investigations will be necessary to define the repertoire of binding peptides presented by HLA-E (and classical HLA-I), in cells treated with this class of drugs. In this regard, peptides originating from cellular stress-related proteins have been reported to influence the inhibitory activity of NK cells through CD94/NKG2A regulation [26]. Moreover, recent high-throughput profiling of HLA-E-presented ligands has revealed a broader repertoire of self-peptides than previously anticipated, which can contribute to HLA-E stabilization and modulation of NK cell responses. These peptides include proteins involved in cancer metastasis and apoptosis regulation, with the capacity to selectively bind either NKG2A or the counterpart NKG2C activating receptor, thereby expanding the regulatory potential on cytotoxic lymphocytes expressing varying levels of CD94/NKG2x receptors [27]. Within this framework, enhancement of the PRT may serve as a pathway contributing to or expanding the repertoire of peptides presented under conditions of nucleolar stress and DNA damage. In addition, HLA-E trafficking patterns and the associated regulatory mechanisms can also be influenced by NSR/DDR signalling events and increased availability of peptides. This modulation could potentially alleviate ER retention and regulate the surface stability of HLA-E, which has recently been identified as a key factor contributing to the regulation of its expression on the cell surface [82].

A further layer of complexity, linked to the genotoxic effects mediated by CX-5461, has also emerged from recent studies revealing its unique and pronounced mutagenic capability, surpassing drugs with similar mechanisms, such as etoposide (a topoisomerase II poison) and pyridostatin (a G-quadruplex binder) [83]. CX-5461’s capacity to induce a distinctive mutational signature, significantly higher than those observed with other treatments across various human cancers, could contribute to generating mutations and novel peptides for presentation on classical and non-classical HLA molecules. Further studies will be needed to investigate this possibility.

The anticipated progression of RNA Pol I inhibitors with reduced genotoxic and mutagenic effects, along with enhanced regulation of effector lymphocyte activity, offers substantial potential for optimizing therapeutic strategies. In this context, the inhibitor BMH-21, a heteroaromatic intercalator, demonstrates a molecular structure and anticancer activity of considerable interest [84–87]. It does not induce the cellular DNA damage response and operates independently of damage signalling, activation of PI3-kinase (ATM, ATR, DNA-PKcs) and repair pathways to activate NSR [18]. Likewise, BMH-21 derivatives retaining the stacking tetracycle have not been observed to elicit significant DDR. These findings indicate that the heterocyclic core itself lacks inherent DNA-damaging properties despite its ability to intercalate DNA [18]. Further optimization of BMH-21 derivatives with enhanced immunomodulatory properties, while minimizing cellular DNA damage, will be critical for advancing NK cell-mediated responses in MM, with broader implications for cancer therapy.

As a final consideration, an intriguing alternative approach is that CX-5461, given its ability to upregulate HLA-E presentation/expression, could be explored within a distinct therapeutic framework. Specifically, its application could be investigated in combination with recently characterized chimeric NKG2A/NKG2C A/C switch receptor-transduced NK cells [88]. These engineered NK cells integrate the high HLA-E binding affinity of the NKG2A ectodomain with the activating signaling potential of the NKG2C endodomain, potentially enabling enhanced and selective cytotoxicity against tumor cells exhibiting elevated HLA-E expression.

Further investigations are warranted to validate these findings obtained using cell lines and patient-derived MM cells, employing optimized preclinical in vivo models and, subsequently, informing the development of clinical protocols. These efforts will focus on RNA Pol I inhibitors in combination with immunotherapeutic agents, aiming to potentiate anti-tumor immune responses and improve therapeutic efficacy. Such strategies may refine current immunotherapy approaches, enhancing precision in counteracting tumor immune evasion.

## Materials and methods

### Cell lines and clinical samples

Peripheral blood mononuclear cells (PBMCs) from healthy donors were isolated from blood samples by Ficoll-Hypaque density gradient centrifugation and used as effector cells. Healthy donors were recruited by the Blood Transfusion Centre of “Policlinico Umberto I” Hospital (Rome, Italy) in anonymized form and gave written informed consent. Cultivated human NK cells were obtained after 10-day co-cultures of PBMCs with irradiated Epstein-Barr virus positive (EBV^+^) RPMI 8866 lymphoblastoid cell line as already described in [89]. On day 10, the cell population was routinely >90% CD56^+^CD16^+^CD3^-^, as assessed by immunofluorescence and flow cytometry analysis.

The human myeloma cell lines SKO-007(J3) and RPMI-8226 have been already described [50] and were kindly provided by Prof. P. Trivedi (University of Rome, Sapienza, Italy). Cells were cultured at 37 °C and 5% CO_2_ in complete medium for no longer than 4 weeks and tested for mycoplasma monthly [Mycoplasma PCR Detection Kit, Applied Biological Materials Inc. (ABM) - Richmond, BC, Canada]. MM cell lines were authenticated as already described in [90]. The human 293 T embryonic kidney cell line was purchased from ATCC and was maintained in Dulbecco’s modified Eagle’s supplemented with 10% FBS, 2 mM L-glutamine, 100 U/ml penicillin and 100 U/ml streptomycin. Bone marrow samples from MM patients were managed at the Division of Hematology, Department of Cellular Biotechnologies and Hematology (University of Rome, Sapienza, Italy) (Supplementary Table II). Informed consent in accordance with the Declaration of Helsinki was obtained from all patients, and approval was obtained from the Ethics Committee of the Sapienza University of Rome (Rif. 5191). Bone marrow mononuclear cells (BMMCs) were isolated from bone marrow aspirates and were cultured in RPMI 1640 complete medium supplemented with the addiction of IL-3 (20 ng/ml) and IL-6 (2 ng/ml) at 1 × 10^6^ cells/ml. In some experiments, BMMCs were depleted of myeloma cells, by negative selection, using anti-CD138 magnetic beads (Miltenyi Biotec. S.R.L. Bologna, IT) and used as effector cells (referred as the CD138^-^ fraction). Purified myeloma cells (more than 90% of them expressing CD138 and CD38) were used as targets in autologous degranulation experiments.

### Reagents and antibodies

The polymerase I inhibitors CX-5421, the ATR inhibitor (AZD6738/Ceralasertib) the AKT inhibitor (MK-2206), the mTOR inhibitor (Everolimus) were purchased from Selleckchem (Houston, Texas, USA), the transcription and splicing inhibitors (Triptolide and Isoginkgetin) and BMH-21 was purchased from Sigma-Aldrich (St. Louis, MO, USA). The highly selective immunoproteasome subunit LMP7 inhibitor M3258, was acquired from MedChemExpress (New Jersey, USA). The final concentration of DMSO in all experiments was maintained below 0.1%. RNA Pol I inhibitors treatment at the concentration used in these experiments [200 nM (CX-5461) and 400 nM (BMH-21)], only minimally affected cell viability of these cell lines after 48 h treatment, as assessed by PI and BD Horizon™ Fixable Viability Staining (data not shown). Daratumumab was provided by the “Policlinico Umberto I” Hospital - hematology center pharmacy. Monalizumab and Lirilumab were acquired from MedChemExpress (New Jersey, USA). The anti NKG2A mAb Z199 was a kind gift of Dr. López-Botet (Department of Medicine and Life Sciences, University Pompeu Fabra, Barcelona, Spain). The following monoclonal antibodies (mAbs) were used for immunostaining: anti-CD14-APC-H7 (MφP9), anti-CD19-APC-H7 (HIB19), anti-CD45-PE-Cy™7 (HI30), anti-CD3-BV510 (HIT3a and UCHT1), anti-CD56-BV421/PE (NCAM16.2), anti-CD16-PerCP-Cy™5.5 (3G8), anti-CD107a-APC (H4A3), anti-CD138-PE (MI15), anti-CD38-APC (HIT2), anti-IFN-γ-PE (4S.B3), anti-NKG2D-APC (1D11), anti-DNAM1-FITC (DX11), anti-NKp30-Alexa Fluor® 647 (p30-15), anti-TIM-3-BB515 (7D3), and anti-PD-1-BV421 (EH12.1), anti-HLA-E-PerCP-Cy5.5 (3D12), anti-FasL-PE (NOK-1) and anti-Trail-APC (RIK-2) all obtained from Becton Dickinson (Franklin Lakes, New Jersey, USA). Additionally, anti-NKp46-PE (9E2) and anti-TIGIT-APC (A15153G) were acquired from BioLegend (San Diego, California, USA), anti-TNFα-FITC (cA2) was purchased from Miltenyi Biotec (Bergisch Gladbach, North Rhine-Westphalia, Germany). Anti-MICA (159227), anti-MICB (236511), anti-ULBP-1 (170818), anti-ULBP-2/5/6 (165903), anti-ULBP-3 (166510), anti-B7-H6 (875001), anti-NECTIN2 (610603) were purchased from R&D System (Minneapolis, MN, USA). Anti-PVR/CD155 (SKII.4) was kindly provided by Prof. M. Colonna (Washington University, St Louis, MO). Anti-MHC class I (W6/32) was purchased from ATCC. Allophycocyanin (APC)-conjugated with goat anti mouse antibody was purchased from Jackson Immuno-Research laboratories. Human recombinant IFN-γ, IL-3 and IL-6 were purchased from PeproTech.

### Flow cytometry, degranulation assay and cell cycle

Degranulation assays were performed using PBMCs from healthy donors or BMMCs from bone marrow aspirates depleted of MM plasma cells (from now referred as CD138^-^ cells) as source of NK effector cells. PBMCs were cultured (in 24 or 12-well tissue culture plates) at 10^6^ cells/ml in complete medium supplemented with 100 U/ml of IL-2 for 48 h. Cells were then (washed in complete medium and) co-cultured with untreated or RNA Pol Is treated SKO-007(J3) or RPMI-8266 cells [Effector:Target (E:T) ratio of 2.5:1] for 2 h at 37 °C and 5% CO_2_ in the presence of anti-CD107a-APC mAb; 50 µM Monensin (Golgi-stop; Sigma-Aldrich). Thereafter, cells were washed with phosphate-buffered saline (PBS) 2% FBS and incubated with anti-CD14-APC-H7, anti-CD19-APC-H7, anti-CD3-BV510 (from now on mentioned as lin^-^), anti-CD45-PE-Cy™7, anti-CD56-BV421, anti-CD16-PerCP-Cy™5.5 for 45 min at 4 °C. In some experiments, SKO-007(J3) cells were pre-treated with anti-CD38/Daratumumab or isotype-control (0.5 µg/10^6^) cells for 15 min at room temperature and then washed twice in complete medium. In other experiments, PBMCs were pre-treated with anti-NKG2D (149810) (R&D System), anti-DNAM-1 (DX11, Bio-Rad), anti-NKp30 (P30-15) BioLegend, anti-NKG2A (clone Z199 or Monalizumab), anti-KIR (Lirilumab) or isotype-control (0.2 to 1 µg/10^6^ cells) for 15 min at room temperature and then washed twice in complete medium. CD107a cell surface expression was evaluated on the NK cell population, gated as lin^-^CD56^+^CD16^+/^, using a FACS Canto II flow cytometer (BD Biosciences). CD138^-^ cells were cultured in complete medium supplemented with IL-2 (100 U/ml) at 3 × 10^6^ cells/ml for 48 h. For autologous degranulation experiments, CD138^-^ cells were co-cultured with autologous patient-derived enriched plasma cells, untreated or treated with RNA Pol Is for 48 h [(E:T ratio of 2.5:1)] for 2 h at 37 °C and 5% CO_2_. Then cells were washed with PBS 2% FBS and incubated with lin^-^, anti-CD45-PE-Cy™7, anti-CD56-BV421, anti-CD16-PerCP-Cy™5.5 to gate NK cells, anti-CD138-PE/FITC to exclude PCs and the degranulation marker anti-CD107a-APC for 45 min at 4 °C. In some experiments, CD138^-^ cells were pre-treated with anti-CD94/NKG2A (Z199) or isotype-control for 15 min at room temperature and then washed twice in complete medium. Samples were acquired using FACS Canto II flow cytometer. In these experiments, autologous MM PCs were cultured (after purification) in complete medium supplemented with the addiction of IL-3 (20 ng/ml) and IL-6 (2 ng/ml) at 1 × 10^6^ cells/ml.

To evaluate cell surface expression of specified NK cell-activating ligands, HLA-E and HLA-ABC in patient-derived malignant PCs, BMMCs were isolated from bone marrow aspirates and treated with Pol I inhibitors or vehicle for 48 h. HLA-E and HLA-ABC expression were evaluated on CD38^+^CD138^+^ PCs.

For cell cycle analysis, cells were harvested after 48 h/treatment, washed in PBS with 0.1% sodium azide, fixed in cold 70% ethanol and incubated at −20 °C o/n. Thereafter, to remove ethanol and precipitated proteins, cells were washed twice with PBS and then incubated with a solution containing propidium iodide (50 μg/mL) and RNAse (100 μg/mL) for 30 min at R/T. using a FACS Canto II flow cytometer (BD Biosciences). The expression levels of the cytokines IFN-γ and TNF-α were analyzed in NK cells gated from PBMCs, co-cultured with RNA Pol I-treated or untreated SKO-007(J3) target cells (E:T ratio 2.5:1) for 6 h at 37 °C and 5% CO_2_. After one-hour Brefeldin A (5 µg/ml) and Monensin (25 µM) (Sigma-Aldrich) were added. Thereafter, cells were washed with PBS 2% FBS and incubated with lin^-^, anti-CD45-PE-Cy™7, anti-CD56-BV421, anti-CD16-PerCP-Cy™5.5. After extracellular staining, cells were fixed and permeabilized using BD Cytofix/Cytoperm™ Fixation/Permeabilization Kit (BD Biosciences) and stained with anti-IFN-γ-PE or anti-TNFα-FITC. Fluorescence was analyzed, on gated NK cells, using a FACS Canto II flow cytometer (BD Biosciences). The expression of different NK cell activating, and inhibitory receptors was analyzed, on gated NK cells from PBMCs (lin^-^, anti-CD45-PE-Cy™7, anti-CD56-BV421/PE^+^, anti-CD16-PerCP-Cy™5.5^+/^) from healthy donors, treated with the drugs. Receptor immunofluorescent staining was performed using the following directly conjugated monoclonal antibodies/mAbs: anti-NKG2D-APC, anti-DNAM1-FITC, anti-NKp30-Alexa Fluor® 647/BB700, anti-NKp46-PE, anti-TIM-3-BB515, anti-PD-1-BV421, anti-TIGIT-APC, anti-FasL-PE, anti-Trail-APC, and analyzed through a FACS Canto II flow cytometer (BD Biosciences). Non-specific fluorescence was assessed by using an isotype-matched control Ig. In all experiments, cells were stained with BD Horizon™ Fixable Viability Stain 780 (BD Biosciences) to assess cell viability and exclude dead cells. To calculate the averages of the different mean fluorescence intensities (MFI) of each sample/treatment, the corresponding MFI of the control isotype was always subtracted from the MFI of the specific mAb (for that treatment). Data were analyzed by FlowJo v10.10 Cytometric Analysis Software (BD Biosciences, San Jose, California, USA). Cell surface binding of Daratumumab to CX-5461 or BMH-21 treated or untreated MM cells, was evaluated by indirect flow cytometry. Daratumumab was used as an unconjugated primary antibody, followed by staining with a FITC-conjugated goat Fab2 anti-human IgG Fc (Cappel Laboratories).

### Analysis of cellular senescence and DNA damage

Senescence-associated β-galactosidase assay was performed using the fluorogenic substrate 5-dodecanoylaminofluorescein di-β-D-galactopyranoside (C_12_FDG) (Invitrogen, Frederick, MD) to measure β-galactosidase activity by flow cytometry [91]. Briefly, after treatment cells were incubated 1 h with 100 nM Bafilomycin A1 to induce lysosomal alkalinization, followed by 1 h incubation with C_12_FDG (33 μM). The C_12_-fluorescein signal of senescent cells was measured on the FL-1 detector using a FACS Canto II flow cytometer. The expression of the DNA damage marker γH2AX on SKO-007(J3) cells was evaluated by the FITC-conjugated anti-γH2AX Ab. After washing, cells were fixed, permeabilized with 70% ethanol, and incubated with anti-γH2AX-FITC (JBW301) Merk/Millipore.

### Confocal microscopy

SKO-007(J3) cells were plated on multi-chamber slides (Falcon) pre-coated with poly-L-lysine (Sigma-Aldrich) and let adhere by centrifugation at 100 × *g*. Cells were then fixed with 4% paraformaldehyde, treated with Glycine 0.1 M for 20 min to quench PFA and permeabilized with 0.1% Triton-X-100 for 5 min. Cells were stained with anti-Fibrillarin (B-1) and anti-NPM1 (FC-8791) (Santa Cruz) for 1 h followed by Alexa Fluor 488-conjugated goat anti-mouse IgG (A11001 - Invitrogen, Life Technologies) for 1 h, all diluted in blocking buffer (PBS 0.01% Triton-X-100, 1% FBS). After extensive washing coverslips were mounted using SlowFade Gold Antifade Mountant with DAPI (S36938 – ThermoFisher Scientific). High-resolution images (1024 × 1024 pixel, 4,10 μs/pixel, zoom 3x) were acquired at room temperature using Zeiss LSM 980 confocal microscope with a Plan-Apochromat 63x/1.40 NA oil immersion objective (all from Zeiss – Jena Germany). Images were processed with ZEISS ZEN 3.7 Software. Data analysis was performed with ZEISS ZEN 3.7 Software.

### Plasmids, virus production and in vitro transduction

For knocking down CBP20, we used the following lentiviral vectors/sequences: pLKO.1-shCBP20 (NCBP2) TRCN0000303544, TRCN0000059994 and the control vector pLKO/puro non-targeting shRNA (MISSION™ Sigma-Aldrich). For lentivirus production, lentiviral vectors were co-transfected together with the packaging vectors pVSVG and psPAX2 into 293 T cells using Lipofectamine 2000 (Invitrogen - Waltham, Massachusetts, USA). After transfection, cells were placed in fresh medium. After a further 48-hour culture, virus-containing supernatants were harvested, filtered and used immediately for infections. Infections were performed on 1.5 × 10^6^ SKO-007(J3) cells in 2 ml virus-containing supernatants with Polybrene (8 μg/ml) (Hexadimethrine bromide - Sigma-Aldrich) for 2 h as previously described [89]. To obtain stable clones, cells were allowed to expand for 24 h and then selected for puromycin (1 μg/ml) resistance.

### mRNA and quantitative real-time polymerase chain reaction (qRT-PCR)

Total RNA was extracted using Total RNA Mini kit (Geneaid Biotech, New Taipei City, Taiwan), according to manufacturer’s instructions. The concentration and quality of the extracted total RNA was determined by measuring light absorbance at 260 nm (A260) and the ratio of A260/A280. Reverse transcription was carried out in a 25 µl reaction volume with 1 µg of total RNA according to the manufacturer’s protocol for M-MLV reverse transcriptase (Promega, Madison, Wisconsin, USA). cDNAs were amplified (TaqMan assays) in triplicate with primers for MICA (Hs00792195_m1), MICB (Hs00792952_m1), PVR (Hs00197846_m1), NECTIN-2 (Hs01071562_m1) and GAPDH (Hs02758991_g1), conjugated to the fluorochrome FAM (Applied Biosystems, Foster City, California, USA). The expression level was measured using the comparative Ct (threshold cycle) method. ΔCt was obtained by subtracting the Ct value of the gene of interest from the selected housekeeping gene (GAPDH) Ct value. In the present study, we used the ΔCt of the control sample as calibrator. The fold change was calculated according to the formula 2^-ΔΔCt^, where ΔΔCt was the difference between ΔCt of the sample and the ΔCt of the calibrator (according to the formula, the value of the calibrator in each run is 1). All PCR reactions were performed using an ABI Prism 7900 Sequence Detection system (Applied Biosystems).

### Western Blot analysis

For Western Blot analyses, cultured MM cells were centrifuged at 1300 RPM for 6 min, washed two times with ice-cold phosphate-buffered saline (PBS) and resuspended in lysis buffer [1 mM EDTA, 50 mM Tris-HCl pH 7.6, 150 mM NaCl, 0.2% Triton X-100, 0.3% Nonidet P-40 (NP-40), 50 mM NaF, 1 mM Na₃VO₄, 1 mM PMSF, Protease Inhibitor Cocktail 1X (Sigma-Aldrich, St. Louis, Missouri, USA), Phosphatase Inhibitor Cocktail 3 1X (Sigma-Aldrich)] before incubation on ice for 20 min. The lysates were centrifuged at 16,000 × *g* for 20 min at 4 °C, and the supernatant was collected as the final cell lysate. Protein concentration was determined using the BCA method (Pierce - ThermoFisher Scientific, Waltham, Massachusetts, USA). Between 15 and 25 µg of protein was resolved on a 10% SDS-polyacrylamide denaturing gel. Proteins were then electroblotted onto Amersham™ Protran™ nitrocellulose membranes (GE Healthcare Life Science, Chicago, Illinois, USA), stained with Ponceau solution to confirm equal protein loading across lanes, and blocked with 5% BSA in blocking buffer. Immunoreactive bands on the nitrocellulose membranes were visualized using primary monoclonal antibodies: anti-HLA-E (MEM-E/02 and 3H2679), anti-HLA-ABC (W6/32), anti-ATR (C1) (Santa Cruz), anti-pS6K (9234), anti-AKT (9272) and anti-S6K (49D7) (Cell Signaling Technology) and horseradish-peroxidase-linked/coupled donkey anti-rabbit (NA934V), sheep anti-mouse (NA931V) IgG (Amersham, GE Healthcare Life Science) and the ECL substrate WESTAR ηC ULTRA 2.0 (Cyanagen, Bologna, Italy), following the manufacturer’s instructions. Results were normalized using specific anti-β-actin (AC-15, Sigma-Aldrich) and anti-p85 (ABS233, Millipore, Massachusetts, USA) antibodies. The iBright Analysis Software (ThermoFisher Scientific) was used for densitometric analysis of the gels acquired using iBright™ CL1500 Imaging System (ThermoFisher Scientific).

### Bulk RNAseq and transcriptomic analysis

Total RNA was extracted using “Total RNA Mini kit” (Geneaid Biotech, New Taipei City, Taiwan), using Ambion™ DNase I (RNase-free), according to manufacturer’s instructions. The concentration and quality of the extracted total RNA were determined by measuring light absorbance at 260 nm (A260) and the ratio of A260/A280 (Qbit System - ThermoFisher). Sequencing libraries were generated using Nextflex Rapid Directional RNA-Seq kit 2.0 Kit with Poly(A) Beads 2.0 (PerkinElmer). Libraries were sequenced on Illumina NovaSeq with 2 × 150 bp paired-end run. Average seq throughput for the project ≥30 M PE read per sample; Q30 ≥ 85%. Transcriptomic/Bioinformatic analysis has been done on Galaxy Europe server. Post-sequencing Quality Control was performed by FastQC v.0.12.1. Adapter sequences were trimmed, and low-quality reads were removed using Trimmomatic v.039. Mapping of RNA and generation of gene counts was done using HISAT2 aligner v.2.2.1 against human reference (GRCh38 build hg38) and FeatureCounts 2.0.8 using locally cached gene annotation. Differential Expression Analysis (DEGs) was performed by DESeq2 v.2.11.40.8. We compared three conditions (Untreated, CX-5461 and BMH-21), with three replicates per condition. For pre-Ranked GSEA analysis, gene ranking has been obtained from normalized DESeq2 results using the pre-ranking metric (−log10 Pvalue * log2 Fold Change). Reactome_RRNA_Processing Signature has been downloaded from - Molecular Signatures Database (MSigDB), https://www.gsea-msigdb.org/gsea/msigdb. To perform single-sample GSEA (ssGSEA), TPMs were calculated using Salmon quant v.1.10.1 (transcriptome: genecode.v46) on Galaxy Europe server, and analyzed via (ssGSEA-module - https://cloud.genepattern.org/, Broad Institute, UC San Diego). Differently from traditional GSEA, which relies on phenotypic differences across sample groups, ssGSEA calculates an enrichment score for each sample individually by comparing the cumulative distribution functions of gene expression ranks inside and outside a given gene set. This score quantifies the coordinated up- or down-regulation of the gene set, effectively turning an individual expression profile into a detailed pathway activity profile. It provides a fine-grained approach to determine pathway enrichment at the single-sample level, offering a robust framework for dissecting biological processes without the need for predefined phenotypic categories. Hallmark gene sets have been downloaded from Molecular Signatures Database (MSigDB). Analysis of alternative splicing and isoform switches with predicted functional consequences has been done using IsoformSwitchAnalyzeR (v 1.20.0) after transcript quantification (Salmon quant v.1.10.1) (transcriptome: genecode.v46) on Galaxy Europe server.

The data generated in this study are publicly available in the Gene Expression Omnibus (GEO) repository, GSE298220.

### Statistical analysis

Error bars represent SEM. Data have been evaluated by paired Student’s *t*-test or ANOVA using GraphPad Prism 8, and a *P* < 0.05 was considered statistically significant.

## Supplementary information

Suppl. Figures - Tables + Legends

Gels Uncropped

## Data Availability

All data generated or analyzed during this study are included in this published article and its supplementary information files. RNA-Seq data are publicly available in the Gene Expression Omnibus (GEO) repository, GSE298220.
