# Supplementary material for: Targeting RNA polymerase I to boost natural killer cell anticancer activity in multiple myeloma
Source: Cell Death Dis. 2025 Nov 28;16(1):865. doi: 10.1038/s41419-025-08196-6 (PMC12663147; doi:10.1038/s41419-025-08196-6)

**SUPPLEMENTARY FIGURES – TABLES and LEGENDS**

**Targeting RNA Polymerase I to boost Natural Killer cell anticancer activity in Multiple Myeloma.**

Elena Sproviero^1^, Eleonora Gnocchini^1^, Tommaso Cipollone^1^, Sara Petillo^2^, Chiara Cassone^1^, Rosa Molfetta^1^, Alessandra Zingoni^1^, Alessandra Soriani^1^, Cristina Cerboni^1^, Maria Teresa Petrucci^3^, Francesca Fazio^3^, Rossella Paolini^1^, Gabriella Palmieri^4^ and Marco Cippitelli^1,§,*^.

^1^Department of Molecular Medicine, Sapienza University of Rome, Italy.

^2^National Eye Institute, NIH, Bethesda, MD, USA.

^3^Hematology, Department of Translational and Precision Medicine, Sapienza University of Rome, Italy.

^4^Department of Experimental Medicine, Sapienza University of Rome, Italy.

^§^Laboratory affiliated to Istituto Pasteur Italia-Fondazione Cenci Bolognetti.

*Correspondence to Marco Cippitelli - Department of Molecular Medicine, University of Rome “La Sapienza” - Viale Regina Elena 291, 00161 - Rome, Italy.

Telephone: +39-0649255152 FAX: +39-0644340632.

Email: [marco.cippitelli@uniroma1.it](mailto:marco.cippitelli@uniroma1.it) or [marco.cippitelli@hotmail.it](mailto:marco.cippitelli@hotmail.it)

**Running title**: Nucleolar Stress Response, HLA-E and MM recognition by NK cells.

**Competing Interests**: The authors declare they have no competing interests.


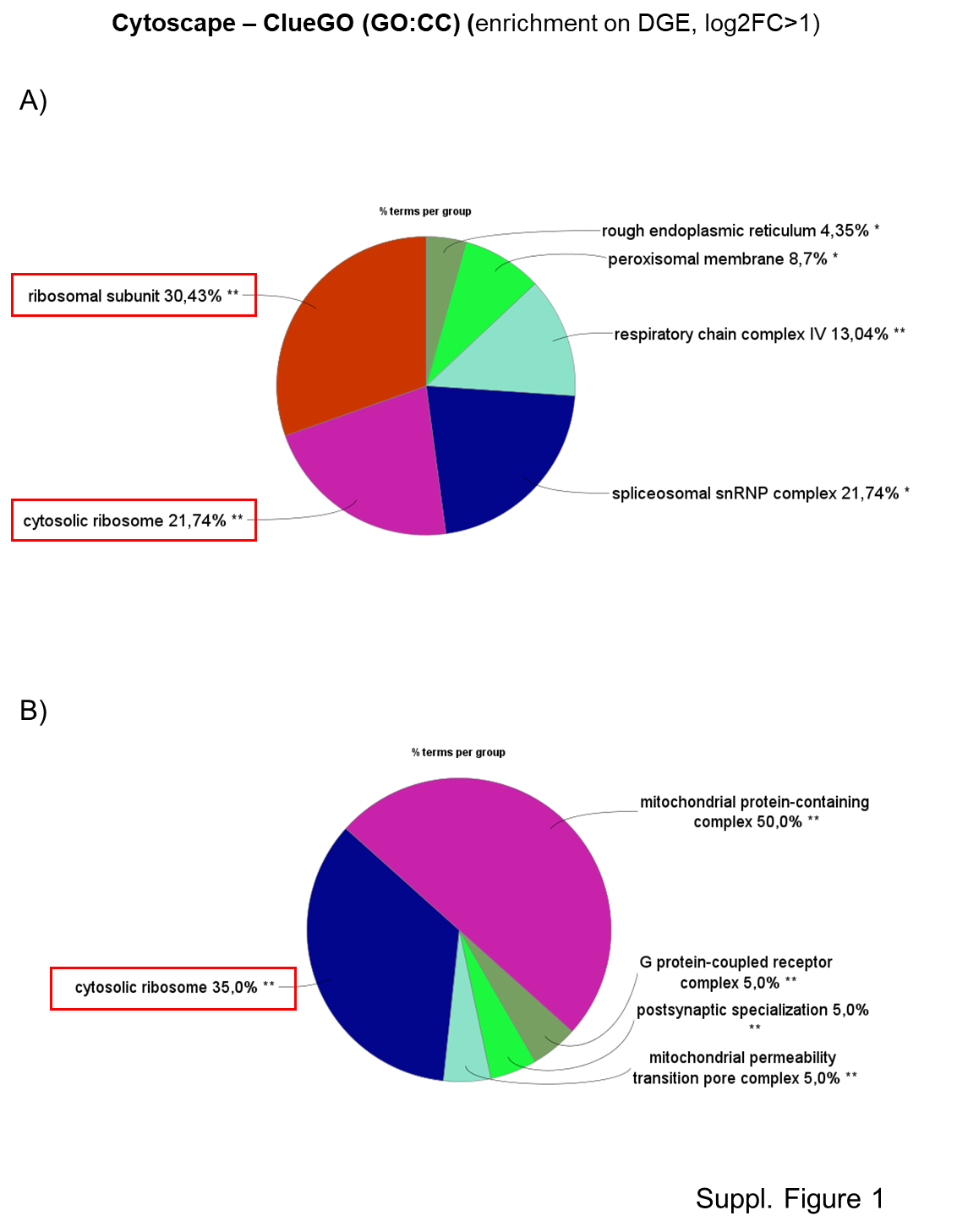


Suppl. Fig. 1 - **Molecular pathways related to ribosomal biogenesis are upregulated in MM.** Gene expression profiling data were collected from GSE47552 and GSE6477 datasets (Gene Expression Omnibus) including Normal Plasma cells (NPC) and Multiple Myeloma plasma cells (MM), and processed via GEO2R web-based bioinformatics analysis platform (<https://www.ncbi.nlm.nih.gov/geo/geo2r/>) to obtain differentially expressed genes (FC >1, p-value adjusted < 0.05), and ClueGO (Cytoscape) for GO - Cellular Component (CC) pathways enrichment terms.


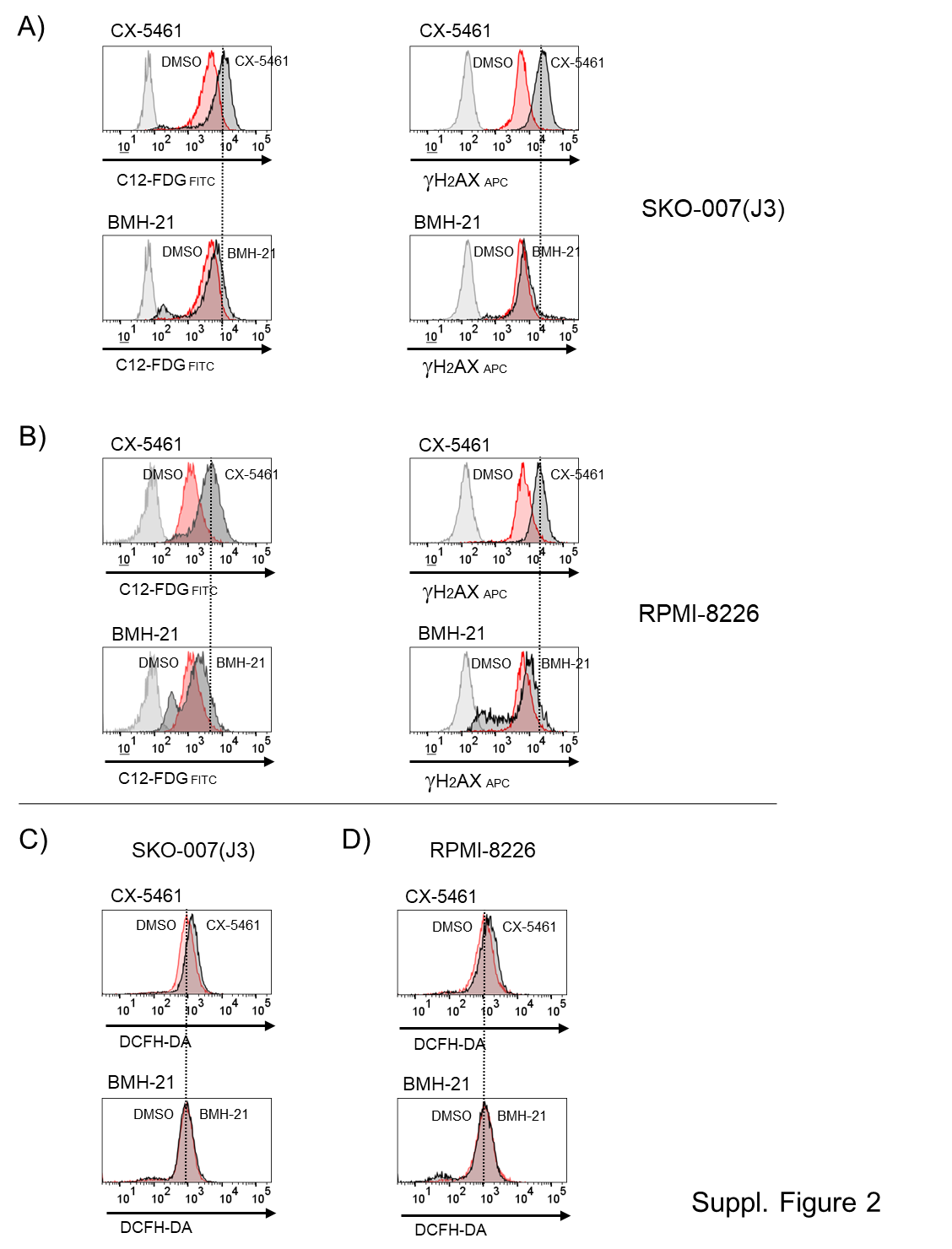


Suppl. Fig. 2 - **RNA Pol I inhibitors exert differential effects on cell senescence, DNA damage and ROS production.** A, B) SKO-007(J3) and RPMI-8226 cells were either untreated or exposed to RNA Pol I inhibitors for 48 hours, then subjected to lysosomal alkalization by incubation with 100 nM bafilomycin A1 for 1 hour, followed by a subsequent 1-hour incubation with 33 µM C12FDG. The mean florescence intensity of SA-βGal-positive cells was then assessed using flow cytometry. Phosphorylation of γH2AX was examined via immunofluorescence and FACS analysis by staining with anti-γH2AX or control IgG antibodies after a 24-hour exposure to RNA Pol I inhibitors. Red histograms represent untreated cells, while dark grey histograms represent RNA Pol I inhibitor-treated cells. Cells were analyzed using a FACS Canto II flow cytometer (BD Biosciences) and FlowJo Cytometric Analysis Software (BD Biosciences). The results shown are representative of one of three independent experiments. C, D) Intracellular ROS levels were assessed by staining SKO-007(J3) and RPMI-8226 cells, either untreated or treated with RNA Pol I inhibitors for 48 hours, using 5 μM DCFH-DA as outlined in the Materials and Methods. The analysis was performed by flow cytometry. Red histograms represent untreated cells, whereas black histograms correspond to RNA Pol I inhibitor-treated cells. The results shown are representative of one of three independent experiments.


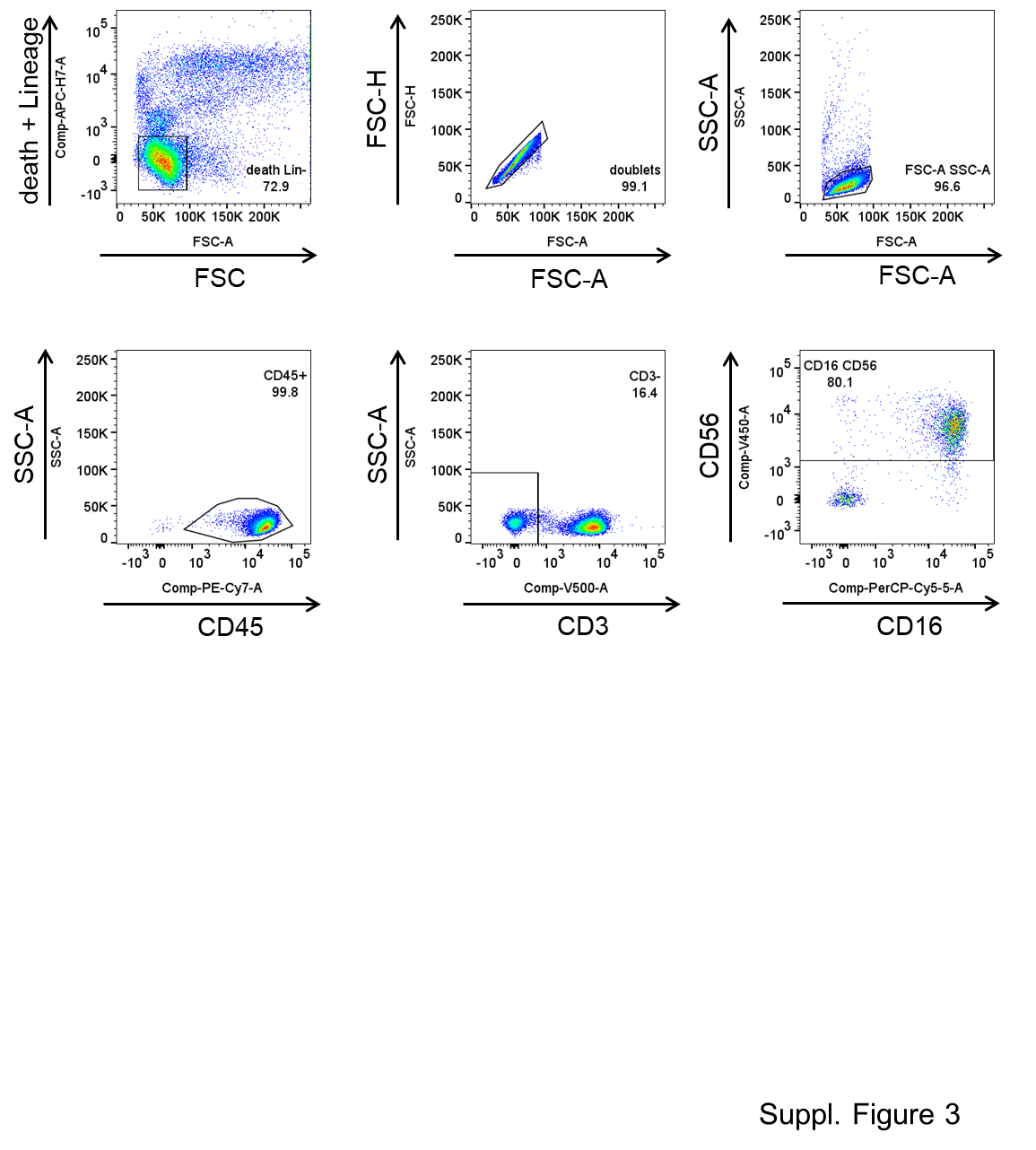


Suppl. Fig. 3 - **Gating strategy to analyze NK cells out of total PBMCs from healthy donors.** CD107a expression was analyzed on CD14^-^CD19^-^CD45^+^CD3^-^CD56^+^CD16^+^ NK cells. Data were analyzed using FlowJo Cytometric Analysis Software (BD Biosciences).


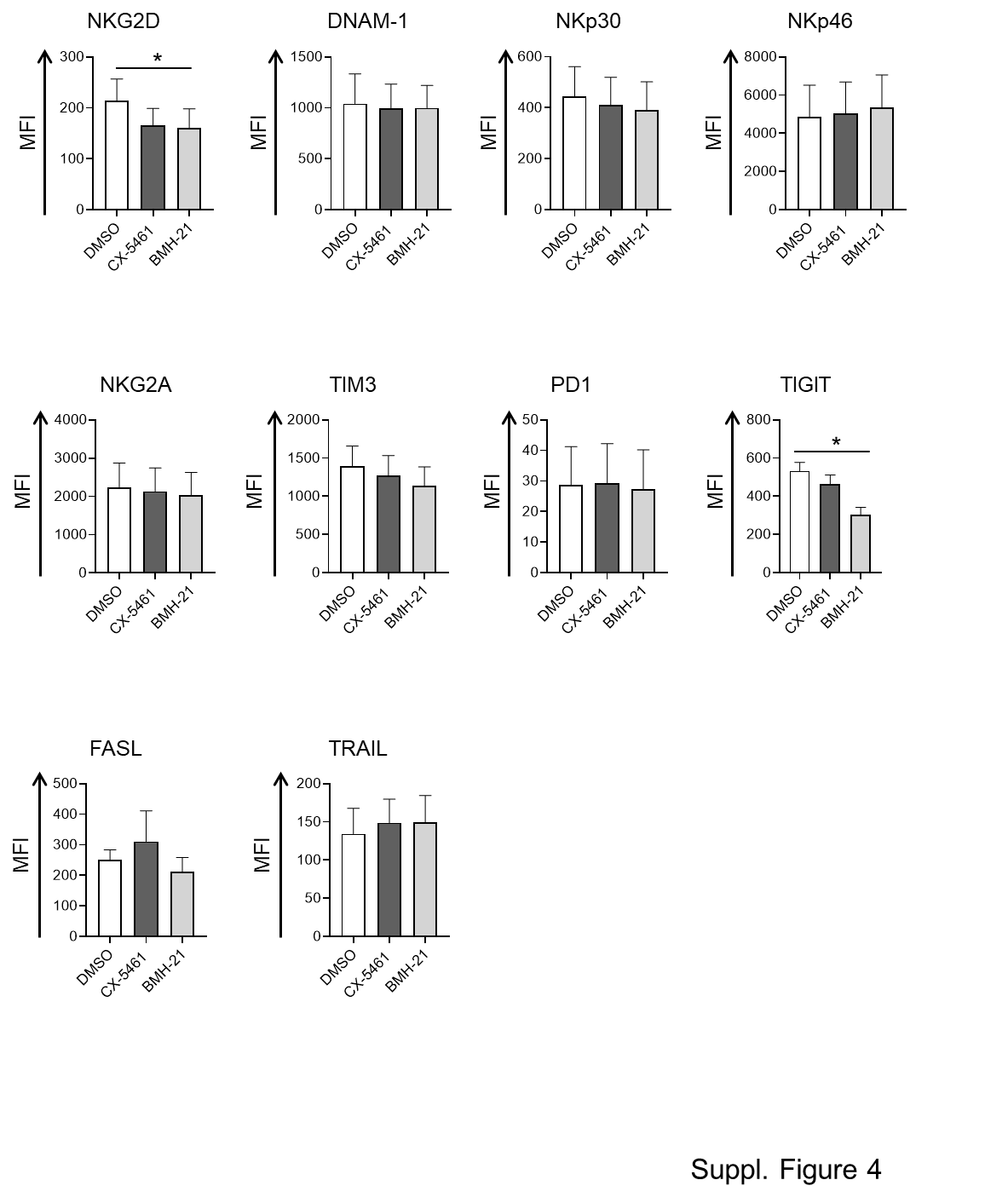


Suppl. Fig. 4 - **Analysis of NK cell activating and inhibitory receptors in RNA Pol I-treated NK cells.** The expression of the indicated receptor was analyzed on CD14^-^CD19^-^CD45^+^CD3^-^CD56^+^CD16^+^ NK cells from RNA Pol I treated or untreated PBMCs (48 h). Histograms represent the results of at least four independent experiments (*P < 0.05). Fluorescence intensity (MFI) was calculated after subtracting the MFI of the isotype control. Data were analyzed by FlowJo Cytometric Analysis Software (BD Biosciences).


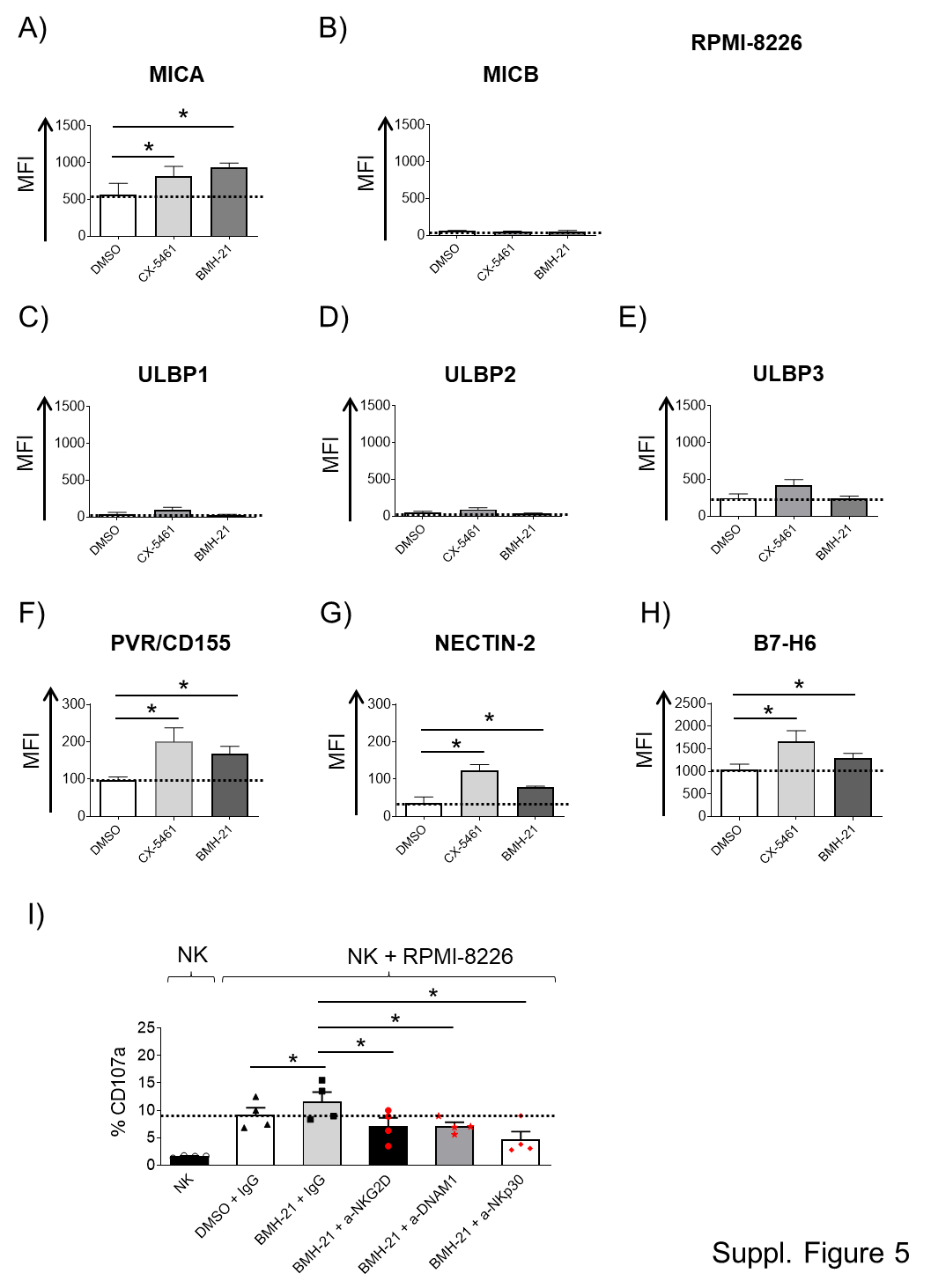


Suppl. Fig. 5 - **Regulation of NK cell-activating ligands expression by RNA Pol I inhibitors differentially in RPMI-8226 MM cells.** A-H) Cell surface expression of the specified ligands was assessed by flow cytometry on RPMI-8226 cells treated with CX-5461 or BMH-21 (200 and 400 nM) for 48 hours. Histograms depict the mean fluorescence intensity (MFI) of the specific monoclonal antibody, after subtracting the MFI of the isotype control (*P < 0.05). Data were analyzed by FlowJo Cytometric Analysis Software (BD Biosciences). I) PBMCs were co-cultured with RPMI-8226 target cells, either untreated or treated with the indicated inhibitors for 48 hours. The assay was conducted at an Effector/Target (E/T) ratio of 2.5:1. To assess the role of NKG2D, DNAM-1 and NKp30 receptors, NK cells pretreated with the specified monoclonal antibodies or IgG control were stimulated with BMH-21-treated target cells. The percentage of CD107a-positive NK cells represents the mean value from at least three independent experiments (*P < 0.05).


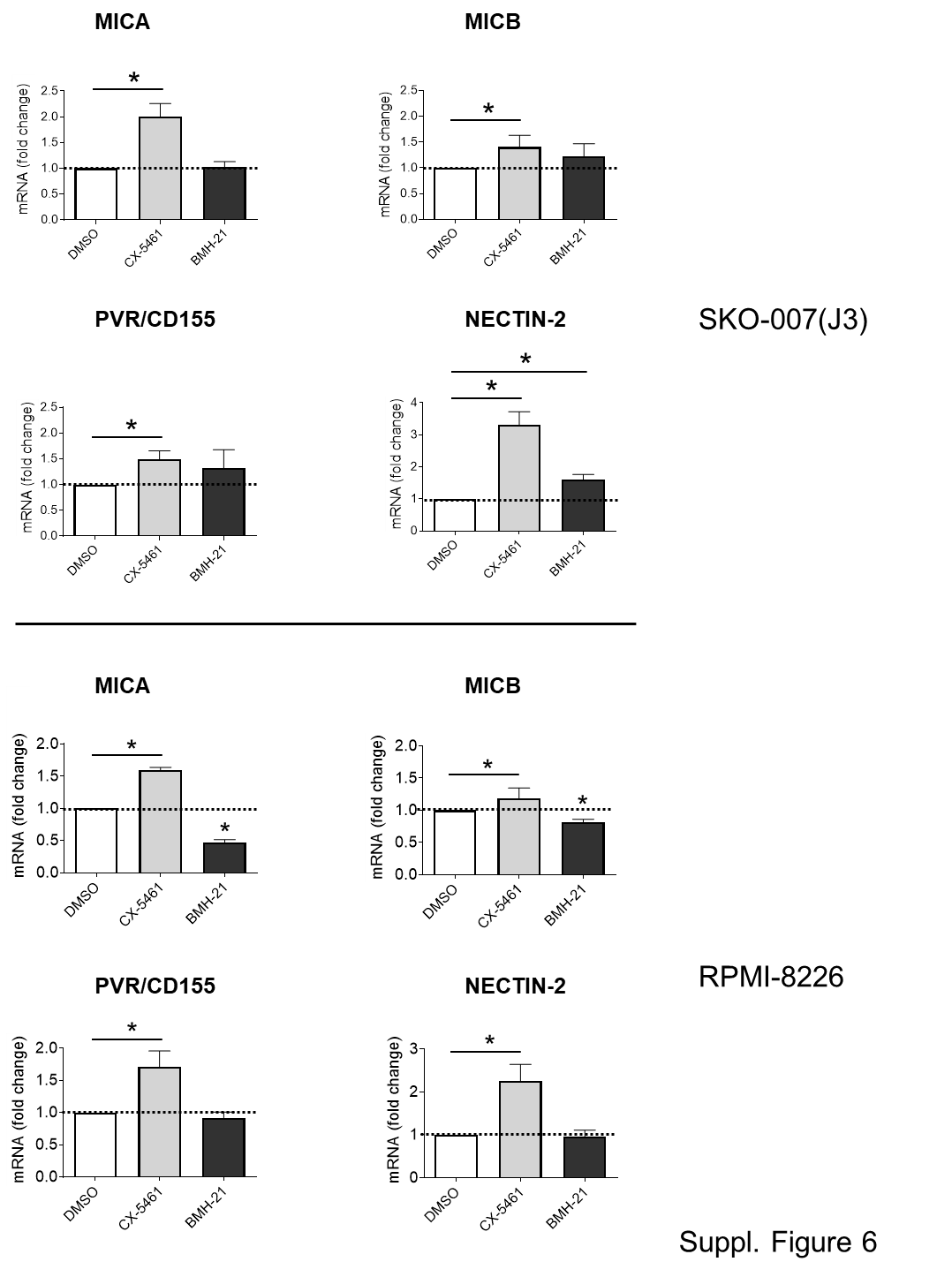


Suppl. Fig. 6 - **RNA Pol I inhibitors differentially regulate MICA/B, NECTIN-2 and PVR/155 mRNA levels in human MM cell lines.** Real Time PCR analysis of total mRNA extracted from SKO-007(J3) and RPMI-8226, untreated or treated with the indicated RNA Pol I inhibitor for 48h. Data, expressed as fold change units, were normalized to GAPDH, and were referred to the untreated sample considered as calibrator (*P < 0.05).


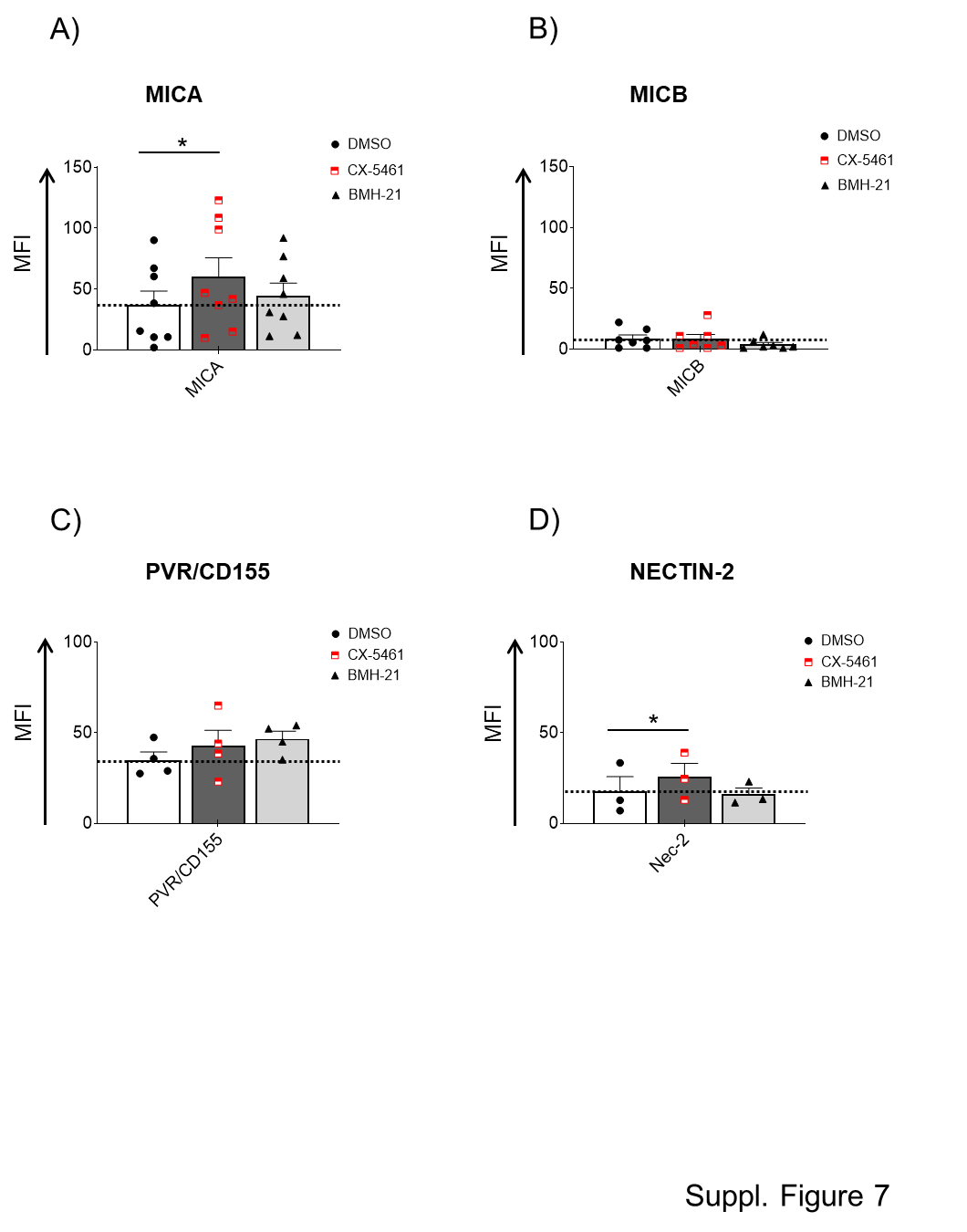


Suppl. Fig. 7 - **RNA Pol I inhibitors differentially regulate NK cell-activating ligands in patient-derived malignant PCs.** A-D) Cell surface expression of the specified ligands was assessed by flow cytometry on patient-derived BMMCs treated with CX-5461 or BMH-21 (200 and 400 nM) for 48 hours. Histograms depict the mean fluorescence intensity (MFI) of the specific monoclonal antibody, after subtracting the MFI of the isotype control (*P < 0.05). The analysis of NK cell-activating ligands was performed on CD38^+^CD138^+^ PCs.


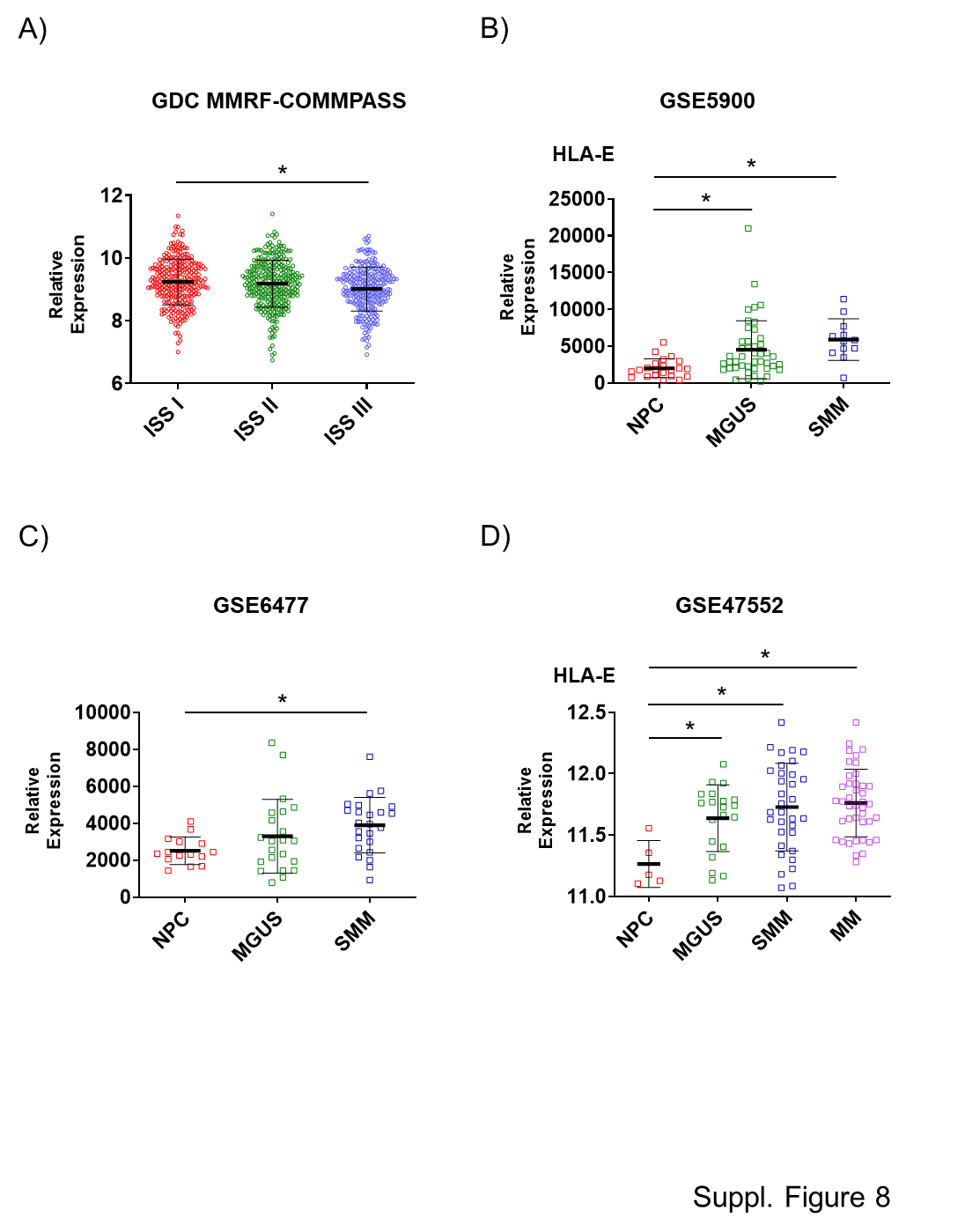


Suppl. Fig. 8 - **Analysis of HLA-E mRNA expression data from the publicly available dataset.**

HLA-E mRNA expression was analyzed using data from the publicly available datasets MMRF-COMMPASS, GSE5900, GSE6477, and GSE47552. These analyses assessed HLA-E expression across various stages of MM progression, including International Staging System (ISS), NPC, MGUS, SMM, and MM. Expression levels are reported as relative values, with statistical significance indicated (*P < 0.05). These analyses were performed using the interactive web-based platforms GEO2R (<https://www.ncbi.nlm.nih.gov/geo/info/geo2r.html>) and the Xena Functional Genomic Explorer (<https://xenabrowser.net/>).


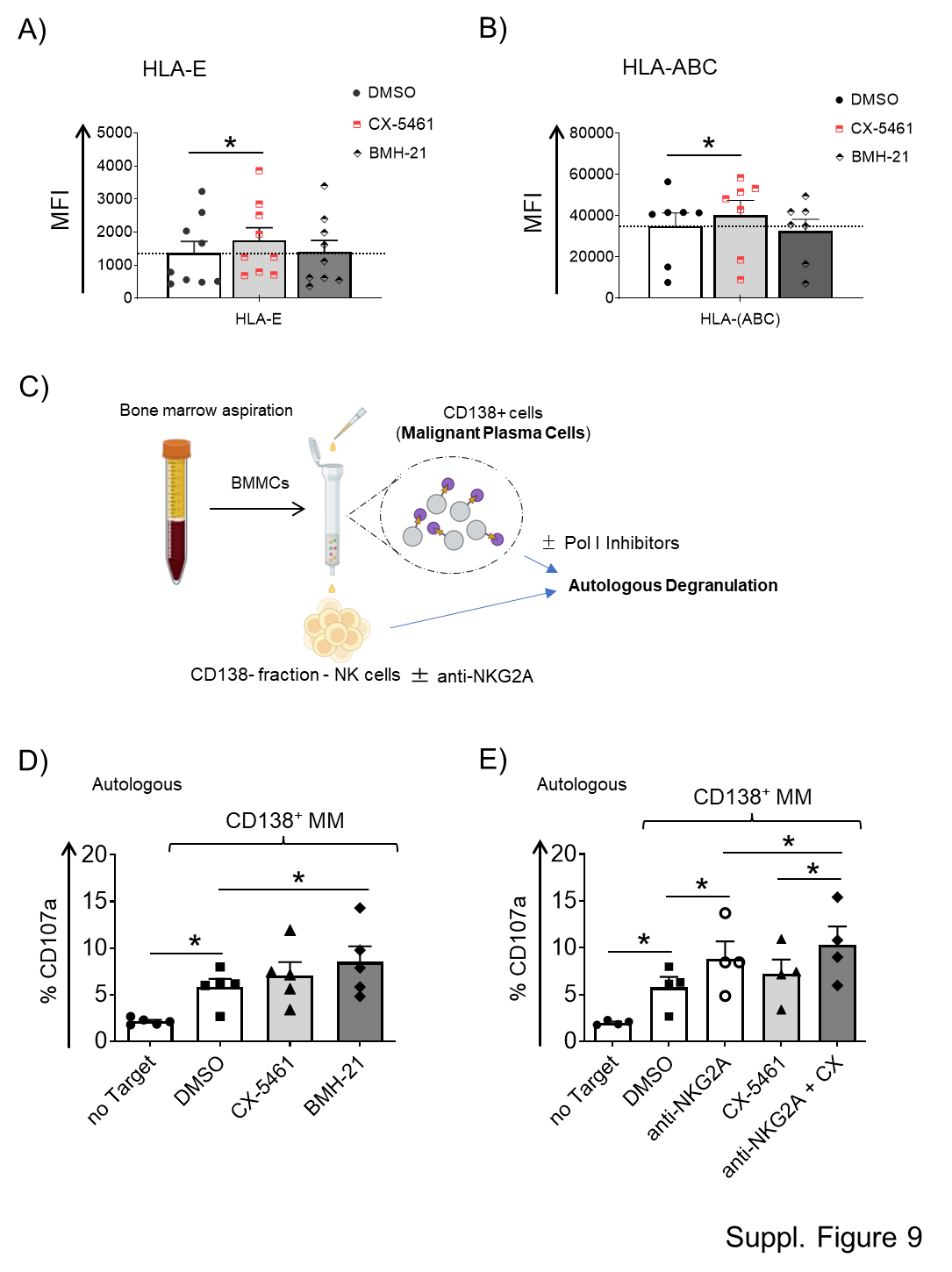


Suppl. Fig. 9 - **RNA Pol I inhibitors differentially regulate HLA-E and HLA-ABC expression and NK susceptibility in patient-derived PCs.** A, B) Cell surface expression of HLA-E and HLA-ABC was analyzed by flow cytometry on patient-derived plasma cells (PCs) treated with CX-5461 or BMH-21 (200 and 400 nM) for 48 hours. Histograms represent the mean fluorescence intensity (MFI) of the specified HLA molecule, with the MFI of the isotype control subtracted (*P < 0.05). Bone marrow mononuclear cells (BMMCs) were isolated from bone marrow aspirates and treated with Pol I inhibitors or vehicle for 48 hours. HLA-E and HLA-ABC expression were evaluated on CD38^+^CD138^+^ PCs. C) Experimental design of the NK cell autologous degranulation assay evaluated via detection of the lysosomal marker CD107a. D) CD138⁻ BMMCs were co-incubated with autologous CD138^+^ MM PCs, either untreated or treated with RNA Pol I inhibitors. The assay was conducted at an E:T ratio of 2.5:1 for 2 hours. The percentage of CD107a-positive NK cells represents the mean value from five independent experiments (*P < 0.05). E) To assess the role of the NKG2A receptor, autologous NK cells pretreated with a blocking mAb for NKG2A (clone Z199) or IgG control, were incubated with malignant PCs treated with CX-5461.


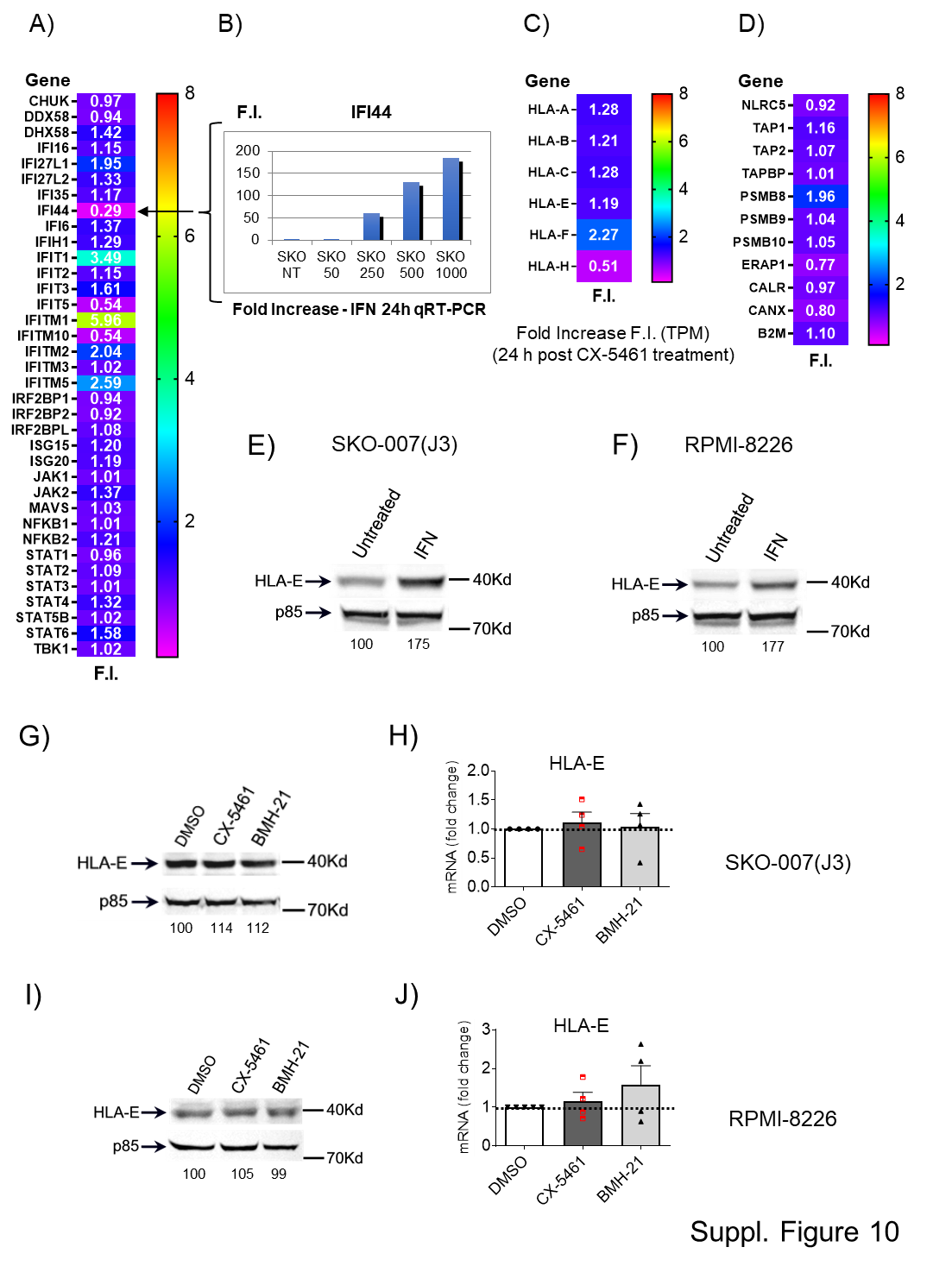


Suppl. Fig. 10 - **CX-5461-induced upregulation of HLA-E on the cell surface is independent of Interferon Signature Gene (ISG) expression and protein level increases.**

A, C, D) RNA-Seq data from SKO-007(J3) cells treated 24h with CX-5461 200 nM. A) Representative ISG and immune-inflammatory responsive genes and C, D) representative Ag presentation-related genes were selected to examine their regulation after CX-5461 treatment. A heat map of the fold increase (F.I.) in TPM compared with untreated cells is shown. B, E, F) Responsiveness of SKO-007(J3) cells to type I interferons (IFN). B) SKO-007(J3) cells were stimulated with the indicated units of IFN-α and the expression of IFI44 was evaluated by qRT-PCR. Data are expressed as fold change units with respect to GAPDH and referred to the untreated sample considered as calibrator (a representative experiment is shown). E, F) IFN treatment (48h) enhances expression of HLA-E in SKO-007(J3) and RPMI-8226 cells. A representative Western Blot image is shown, accompanied by densitometric analysis normalized to p85. G-J) RNA Pol I inhibitors do not increase HLA-E mRNA or protein levels in SKO-007(J3) and RPMI-8226 cells. For qRT-PCR and Western Blot assays cells were simulated with RNA Pol I inhibitors for 48h. A representative Western Blot is shown, accompanied by densitometric analysis normalized to p85. Data of qPCR analysis, expressed as fold change units, were normalized to GAPDH and were referred to the untreated sample considered as calibrator.


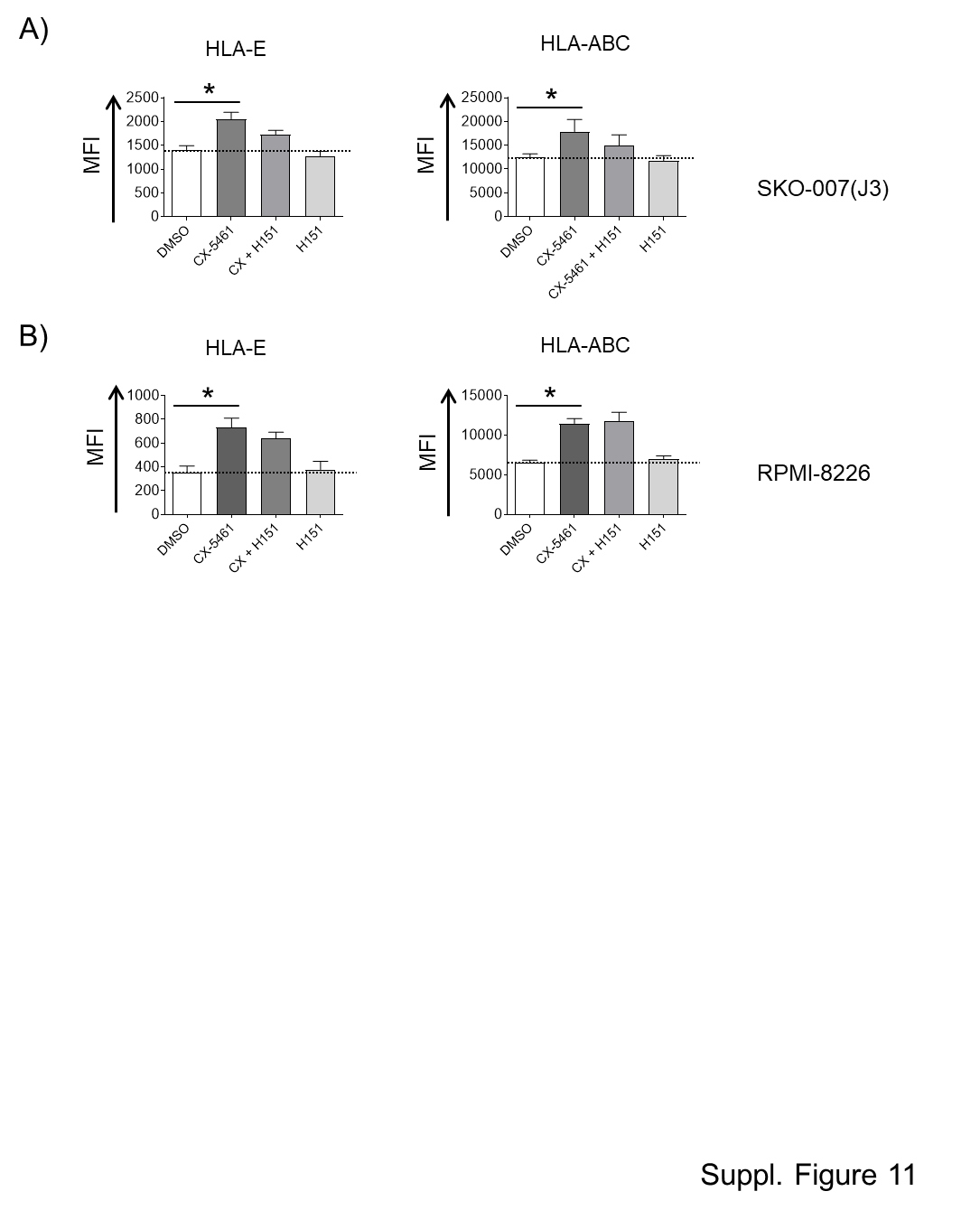


Suppl. Fig. 11 - **Increased HLA-E expression by CX-5461 is not associated with activation of STING.** Cell surface expression of HLA-E and HLA-ABC was analyzed by flow cytometry on SKO-007(J3) and RPMI-8226 cells treated with CX-5461 (200 nM), either in the absence or presence of the STING inhibitor H151 (1 µM) for 48 hours. Histograms depict the mean fluorescence intensity (MFI) of the specific monoclonal antibody, after subtracting the MFI of the isotype control (*P < 0.05).


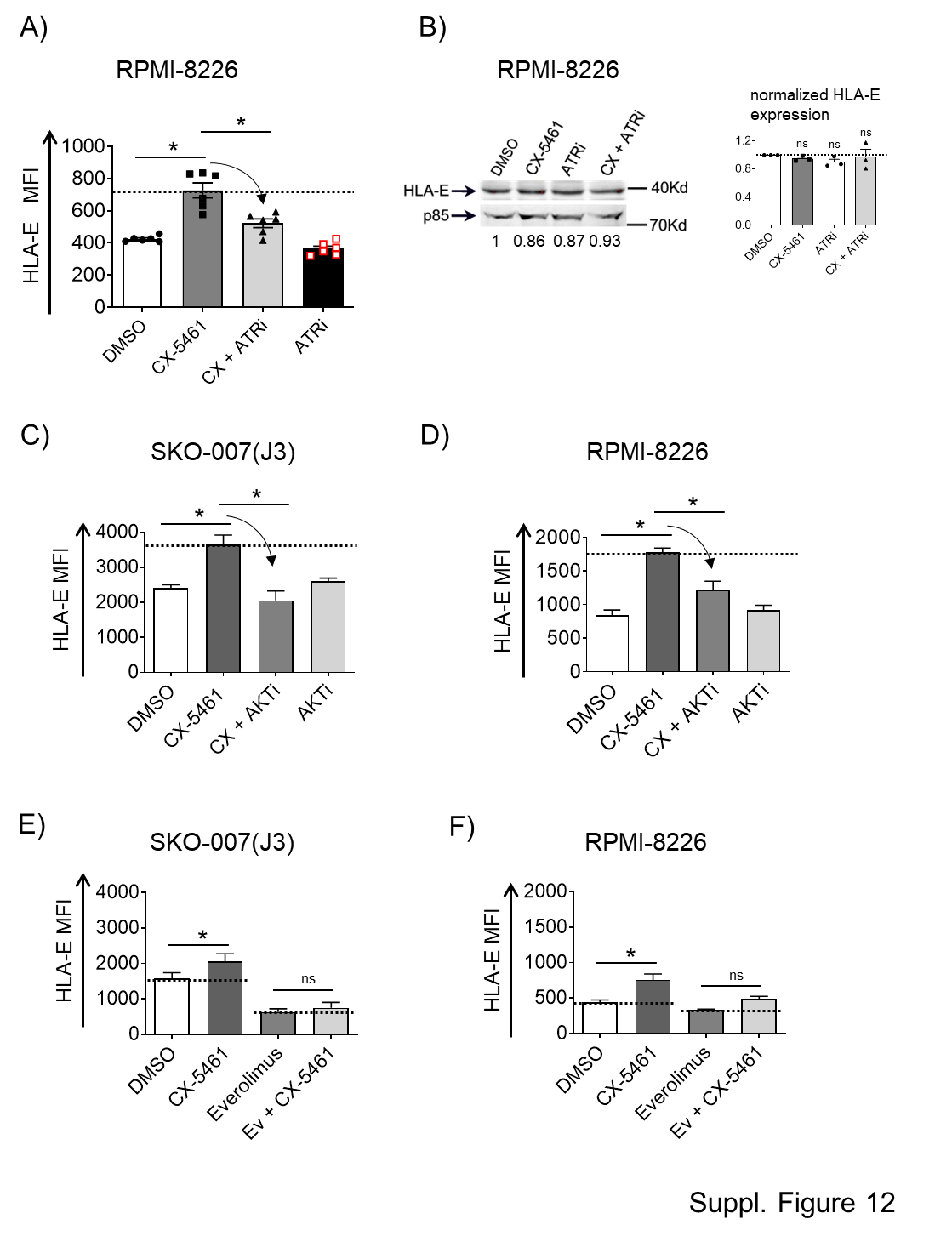


Suppl. Fig. 12 - **Upregulation of HLA-E by CX-5461: role of ATR, AKT, mTORC-1 activity.** A) Cell surface expression of HLA-E was assessed by flow cytometry on RPMI-8226 cells treated with CX-5461in the absence or in the presence of the ATRi (AZD6738/Ceralasertib, 1 µM) for 48 hours. Histograms depict the mean fluorescence intensity (MFI) of the indicated HLA, after subtracting the MFI of the isotype control (*P < 0.05). B) Western Blot analysis of HLA-E in RPMI-8226 cells untreated or treated with the indicated inhibitors for 48 h. Expression of p85 was used as protein loading control. A representative Western Blot is shown with densitometric analysis normalized to p85, together with the quantification of three independent experiments (*P < 0.05). C-F) Cell surface expression of HLA-E was assessed by flow cytometry on SKO-007-(J3) or RPMI-8226 cells treated with CX-5461 in the absence or in the presence of a AKT inhibitor (MK-2206, 5 µM) or mTORC-1 inhibitor (Everolimus, 100 nM) for 48 hours. Histograms depict the mean fluorescence intensity (MFI) of the indicated HLA, after subtracting the MFI of the isotype control (*P < 0.05).


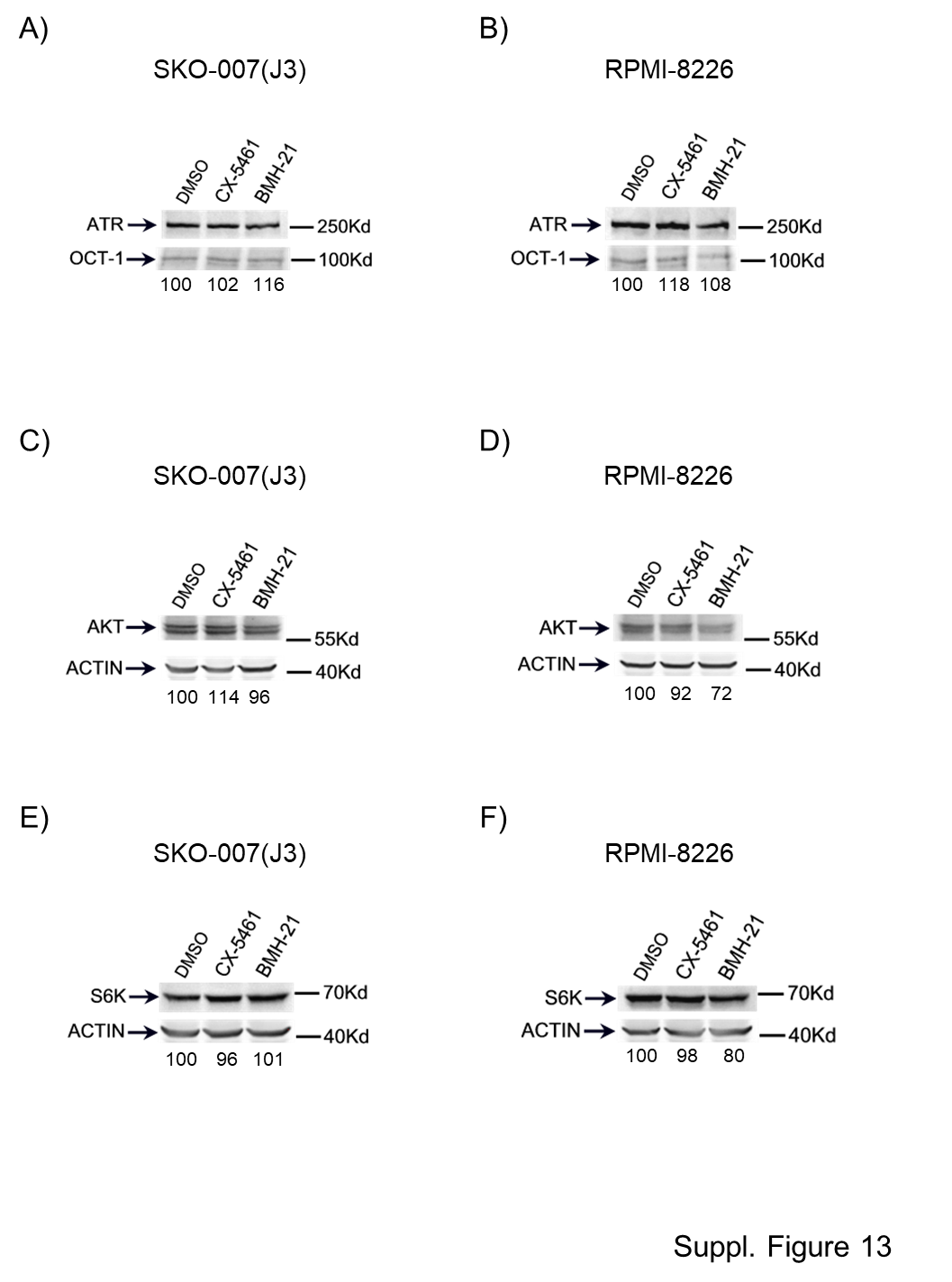


Suppl. Fig. 13 - **Expression levels of the ATR and AKT kinases in MM cells treated with RNA Pol-I inhibitors.** (A, B), (C, D) and (E-F) SKO-007(J3) and RPMI-8226 MM cells were treated with the indicated RNA Pol-I inhibitors [CX-5461 or BMH-21 (200 and 400 nM)] for 48h, and protein expression levels of ATR and AKT and S6K were assessed by Western Blot. A representative experiment is shown, accompanied by densitometric analysis normalized to OCT-1 (for ATR) and β-Actin (for AKT and S6K).


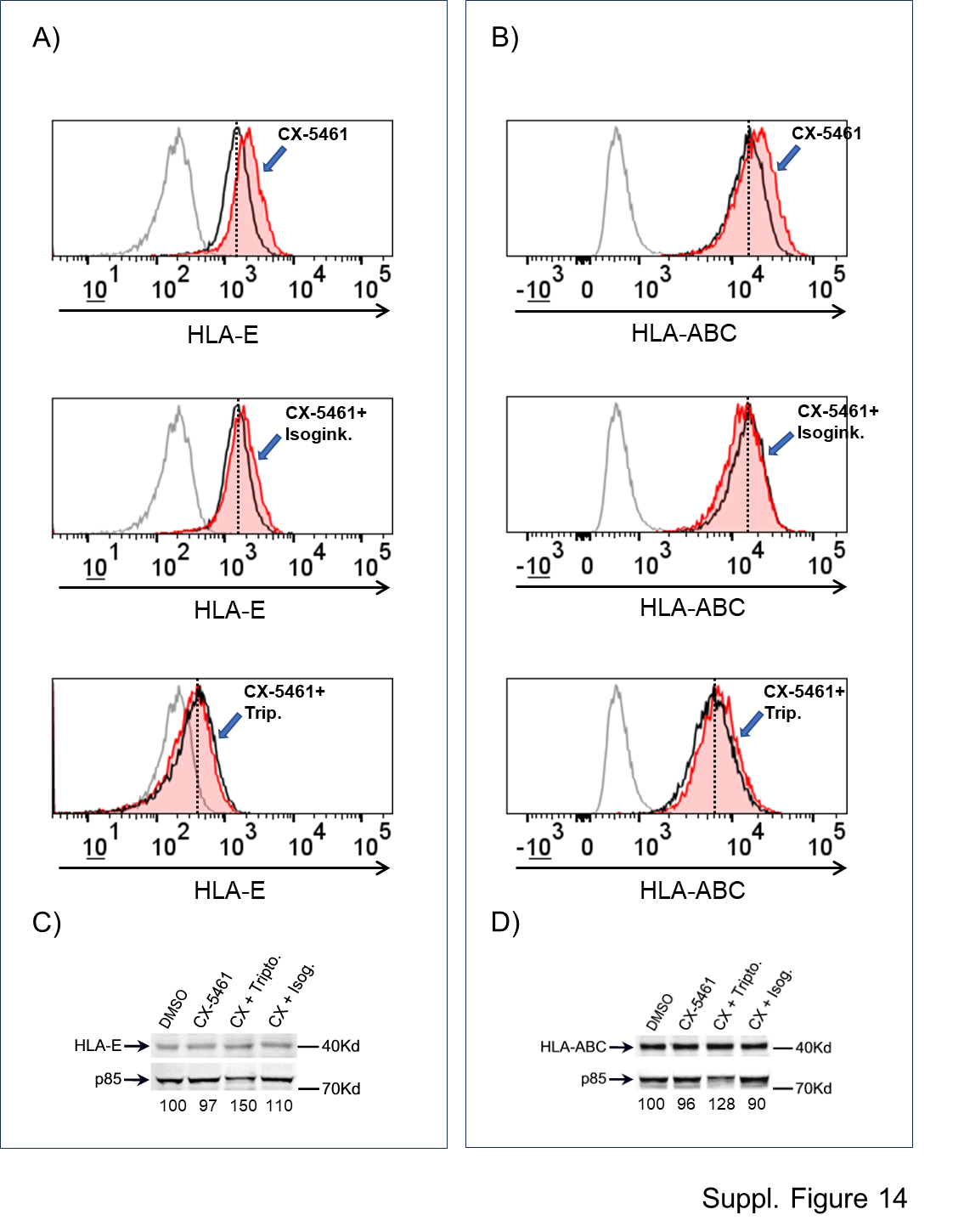


Suppl. Fig. 14 - **Upregulation of HLA-E by CX-5461 requires transcription and splicing.** A, B) Cell surface expression of HLA-E and HLA-ABC was analyzed by flow cytometry on SKO-007(J3) cells treated with CX-5461, either in the absence or presence of the transcription and splicing inhibitors Triptolide (0.5 µM), and Isoginkgetin (5 µM), for 48 hours. Representative overlays are shown. The black histogram illustrates basal expression, while the red histogram represents regulation mediated by the applied inhibitors. C, D) Western Blot analysis of HLA-E and HLA-ABC expression were performed on SKO-007(J3) cells that were either untreated or treated with the specified inhibitors for 48 hours. A representative Western Blot image is shown, accompanied by densitometric analysis normalized to p85.


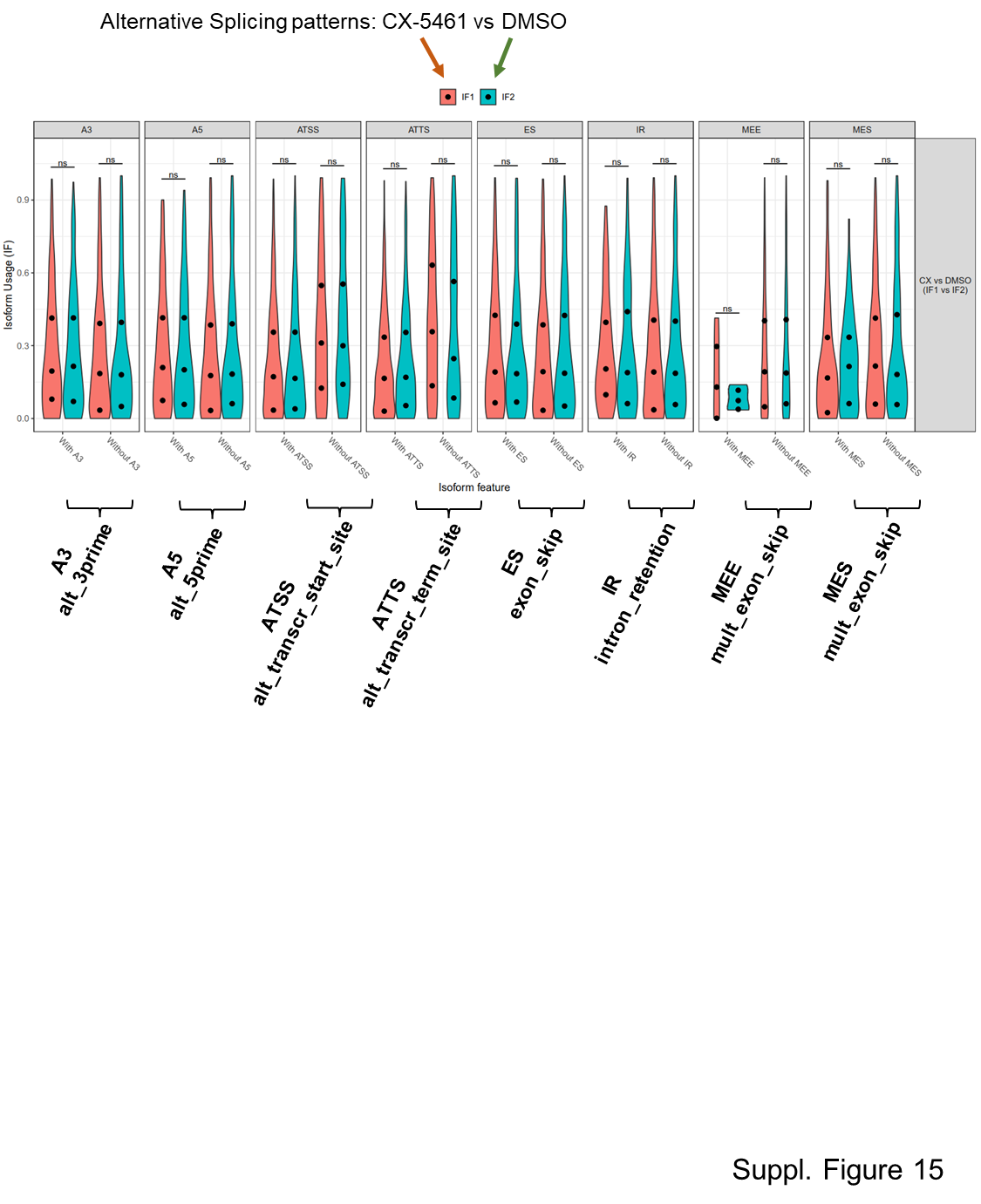


Suppl. Fig. 15 - **Alternative splicing patterns are not significantly changed in SKO-007(J3) cells treated with CX-5461.** RNA-seq data from SKO-007(J3) cells, either untreated or treated with 200 nM CX-5461 for 24 hours, were analyzed to identify splicing patterns. Violin plots illustrate the global distribution of isoform usage (IF) values across splicing events. Alternative splicing events and isoform switches with potential functional implications were assessed using IsoformSwitchAnalyzeR (v 1.20.0).


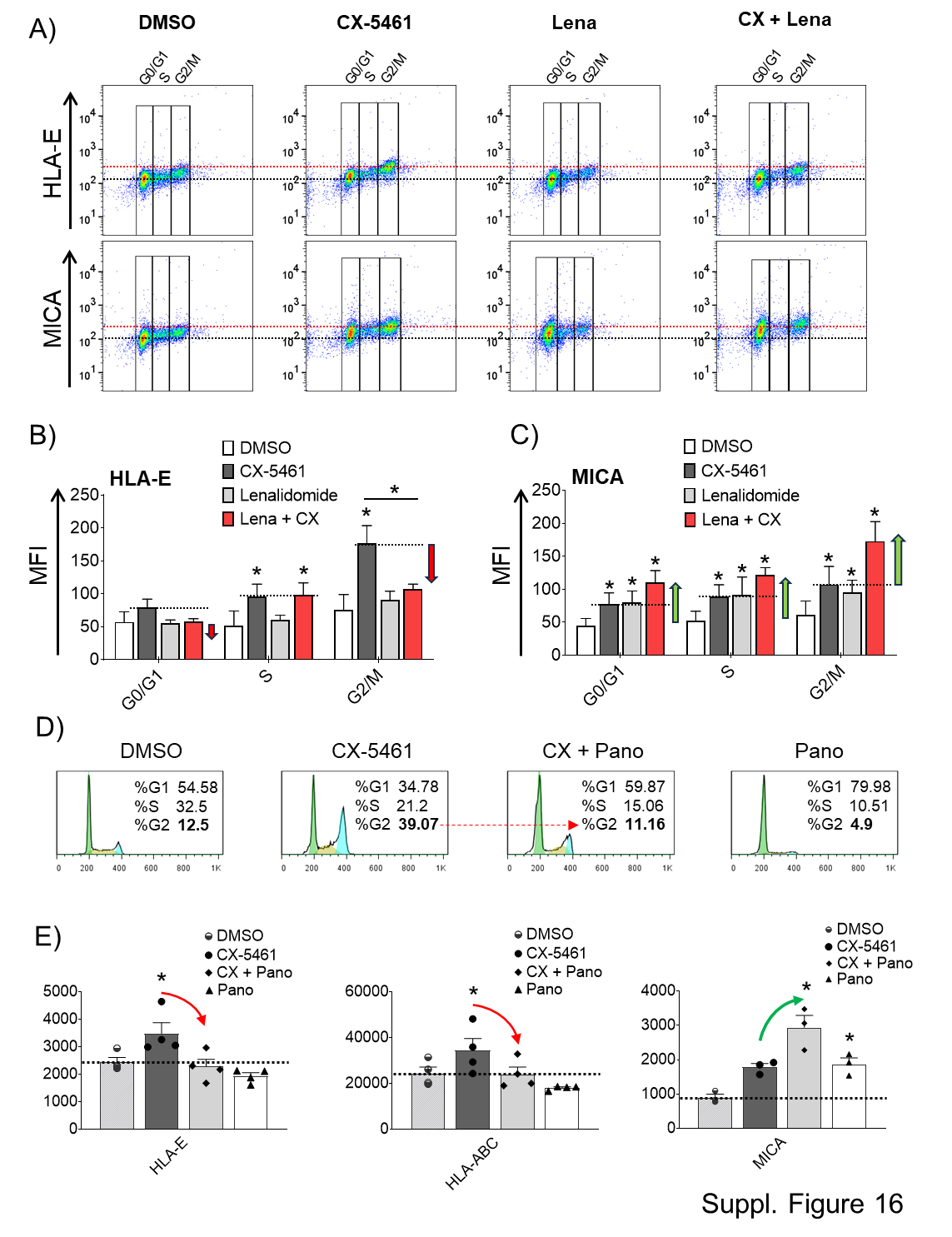


Suppl. Fig. 16 - **Upregulation of HLA-E expression by CX-5461 is predominant in the G2 phase of the cell cycle. Modulation by Lenalidomide and Panobinostat.** A) Cell cycle analysis of SKO-007(J3) cells treated with CX-5461 in the absence or in the presence of Lenalidomide (5 µM - 48 h). A representative experiment of the expression of HLA-E and MICA in the distinct phases of the cell cycle is shown. In figures B and C, histograms depict the mean fluorescence intensity (MFI) of HLA-E or MICA in the distinct phases of the cell cycle, after subtracting the MFI of the isotype control. Red and green arrows highlight the downregulation, or the upregulation as compared to CX-5461 treated cells (*P < 0.05). D) Cell cycle analysis of SKO-007(J3) cells treated with CX-5461 (200 nM) in the absence or in the presence of Panobinostat (10 nM - 48 h). A representative experiment is shown. E) Cell surface expression of HLA-E, HLA-ABC and MICA were analyzed by flow cytometry on SKO-007(J3) cells treated with CX-5461 (200 nM) either in the absence or presence of Panobinostat (10 nM) for 48 hours. Histograms depict the mean fluorescence intensity (MFI) of the indicated ligand, after subtracting the MFI of the isotype control. Red and green arrows highlight the downregulation, or the upregulation as compared to untreated cells (*P < 0.05).


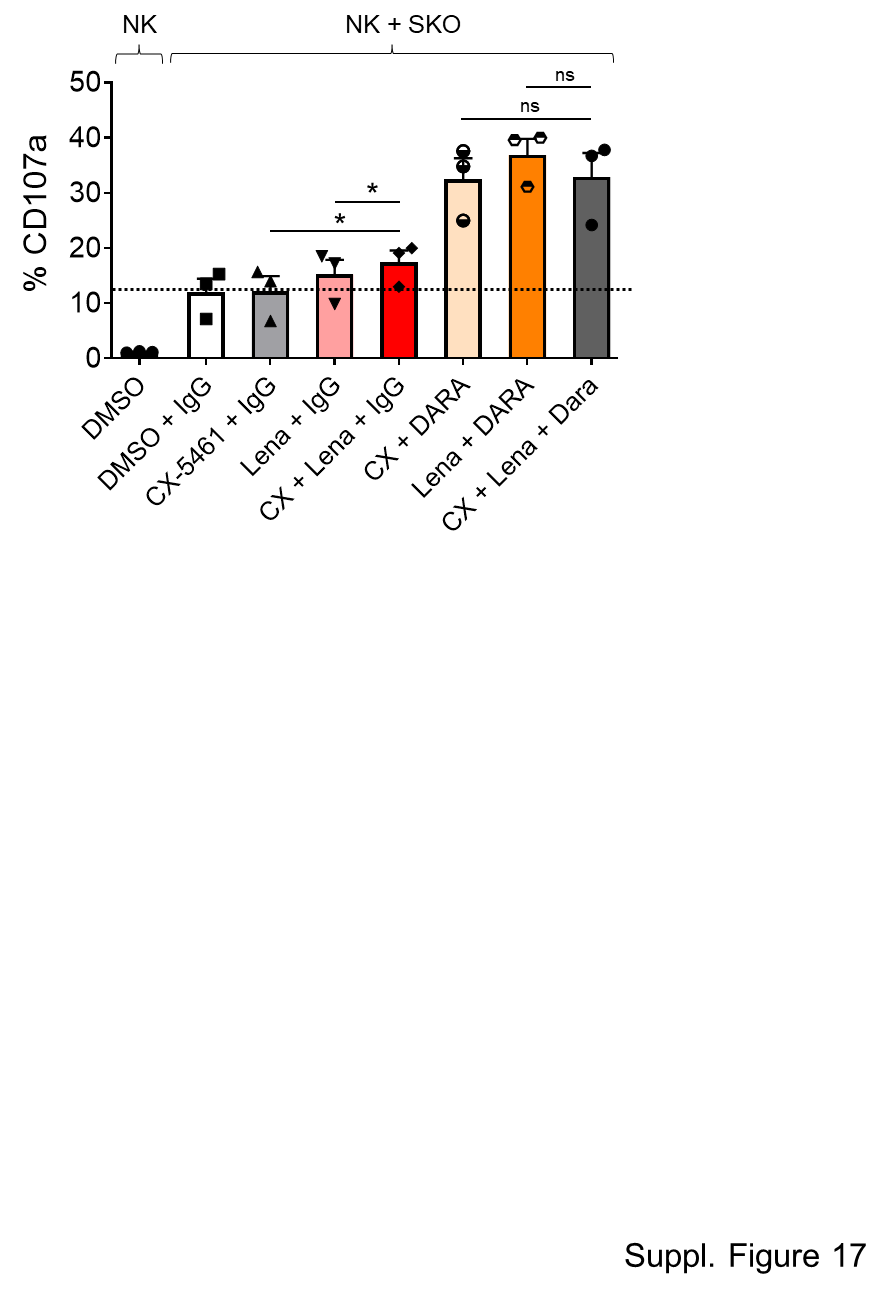


Suppl. Fig. 17 - **Co-treatment with CX-5461 and Lenalidomide does not enhance Daratumumab-mediated ADCC beyond the levels observed with either drug alone.** NK cell degranulation was evaluated using the lysosomal marker CD107a as described above. As source of effector cells, PBMCs purified from healthy donor blood were used as described previously. Cells were co-cultured with the SKO-007(J3) untreated or treated with CX-5461 and/or Lenalidomide for 48 h. Where indicated, anti-CD38/Daratumumab or isotype-control (0.5 µg/10^6^) were added to target cells for 15 min at room temperature and then washed twice in complete medium. The assay was performed at the Effector/Target (E/T) ratio of 2.5:1 in complete medium at 37°C and 5% CO_2_ for 2 h. CD107a expression was evaluated on NK cells gated as CD14^-^CD19^-^CD45^+^CD3^-^CD138^-^CD56^+^CD16^+^, using a FACS Canto II flow cytometer (BD Biosciences) and data were analyzed by FlowJo V10 Cytometric Analysis Software (BD Biosciences (*P < 0.05).

Suppl. Table 1

**Mean Gene Effect (MGE) (Chronos) of 19 Myeloma models CRISPR (DepMap Public 24Q4+Score, Chronos). 100 most essential genes.**

| **gene name** | **MGE (Chronos)** | |
| --- | --- | --- |
| RAN | -3.806825743 |  |
| HSPE1 | -3.586865762 |  |
| RPL17 | -3.081339016 |  |
| RPS29 | -3.011752358 |  |
| RRM1 | -2.937257824 |  |
| PCNA | -2.917903443 |  |
| SF3B5 | -2.865666777 |  |
| RPS19 | -2.767295072 |  |
| RPL23 | -2.765949913 |  |
| RPS20 | -2.757714572 |  |
| RPS8 | -2.743533005 |  |
| SNRPA1 | -2.722653522 |  |
| SMU1 | -2.719586588 |  |
| RPS15A | -2.718997776 |  |
| PRELID1 | -2.709685961 |  |
| RPS4X | -2.68496888 |  |
| PLK1 | -2.662360643 |  |
| PSMB3 | -2.641724656 |  |
| RPL12 | -2.637487023 |  |
| PSMA3 | -2.637283545 |  |
| PUF60 | -2.624268671 |  |
| RPL3 | -2.619862556 |  |
| SF3B3 | -2.59861067 |  |
| UBL5 | -2.590690015 |  |
| PSMA6 | -2.589597716 |  |
| RPL31 | -2.58781981 |  |
| RPL8 | -2.580923705 |  |
| RPL11 | -2.57494409 |  |
| RPL5 | -2.567831733 |  |
| CDC27 | -2.562238799 |  |
| LRR1 | -2.555481834 |  |
| SRSF3 | -2.542577714 |  |
| SNRPF | -2.536952659 |  |
| FAU | -2.529901908 |  |
| PSMA7 | -2.52601588 |  |
| RPS11 | -2.504571703 |  |
| VCP | -2.491323738 |  |
| RRM2 | -2.480002341 |  |
| RPS3 | -2.454121469 |  |
| PRPF38A | -2.428677386 |  |
| RPL13 | -2.423193027 |  |
| MYC | -2.398647899 |  |
| ATP6V0C | -2.390943147 |  |
| SPC24 | -2.388624703 |  |
| RPS18 | -2.367133286 |  |
| RPS23 | -2.358716241 |  |
| PHF5A | -2.35412954 |  |
| SRSF2 | -2.3485273 |  |
| WEE1 | -2.348031075 |  |
| POLR2B | -2.341249781 |  |
| RPL18A | -2.336739199 |  |
| SNU13 | -2.331426966 |  |
| SNRNP200 | -2.326123335 |  |
| PSMA2 | -2.321384078 |  |
| EEF2 | -2.318625564 |  |
| KIF11 | -2.307323294 |  |
| HSPA9 | -2.307033127 |  |
| TXNL4A | -2.300887661 |  |
| SF3A3 | -2.294169008 |  |
| DHDDS | -2.287915454 |  |
| AURKB | -2.286534036 |  |
| CDK1 | -2.284890712 |  |
| PRPF19 | -2.283777297 |  |
| RPS12 | -2.283299376 |  |
| RPL32 | -2.281305454 |  |
| SRP54 | -2.281175949 |  |
| RPS6 | -2.280270041 |  |
| RPL10A | -2.280163214 |  |
| LSM2 | -2.275701801 |  |
| RPL7A | -2.267904572 |  |
| BUB3 | -2.257112181 |  |
| PSMB4 | -2.255741452 |  |
| POLR2L | -2.255628891 |  |
| RPS15 | -2.255602868 |  |
| TOP2A | -2.244128509 |  |
| RPS14 | -2.226918372 |  |
| ABCE1 | -2.215079027 |  |
| RBM39 | -2.206165698 |  |
| COPB1 | -2.205957507 |  |
| VPS25 | -2.201983587 |  |
| CDC7 | -2.199870496 |  |
| COPB2 | -2.195755298 |  |
| SF1 | -2.194806896 |  |
| KPNB1 | -2.19168589 |  |
| CCT4 | -2.190517705 |  |
| WDR82 | -2.182723238 |  |
| PSMA1 | -2.180804875 |  |
| DTL | -2.180153801 |  |
| BANF1 | -2.175835068 |  |
| SNRPD1 | -2.175114415 |  |
| POLD1 | -2.173115036 |  |
| NIP7 | -2.163075595 |  |
| HNRNPK | -2.159628375 |  |
| DUT | -2.158225656 |  |
| ISCU | -2.157847421 |  |
| RPS16 | -2.138843806 |  |
| EIF4A3 | -2.128488546 |  |
| PPWD1 | -2.127291031 |  |
| MED30 | -2.124706347 |  |
| CDC45 | -2.123527836 |  |

Suppl. Table 2

**Clinical parameters of MM Patients**.


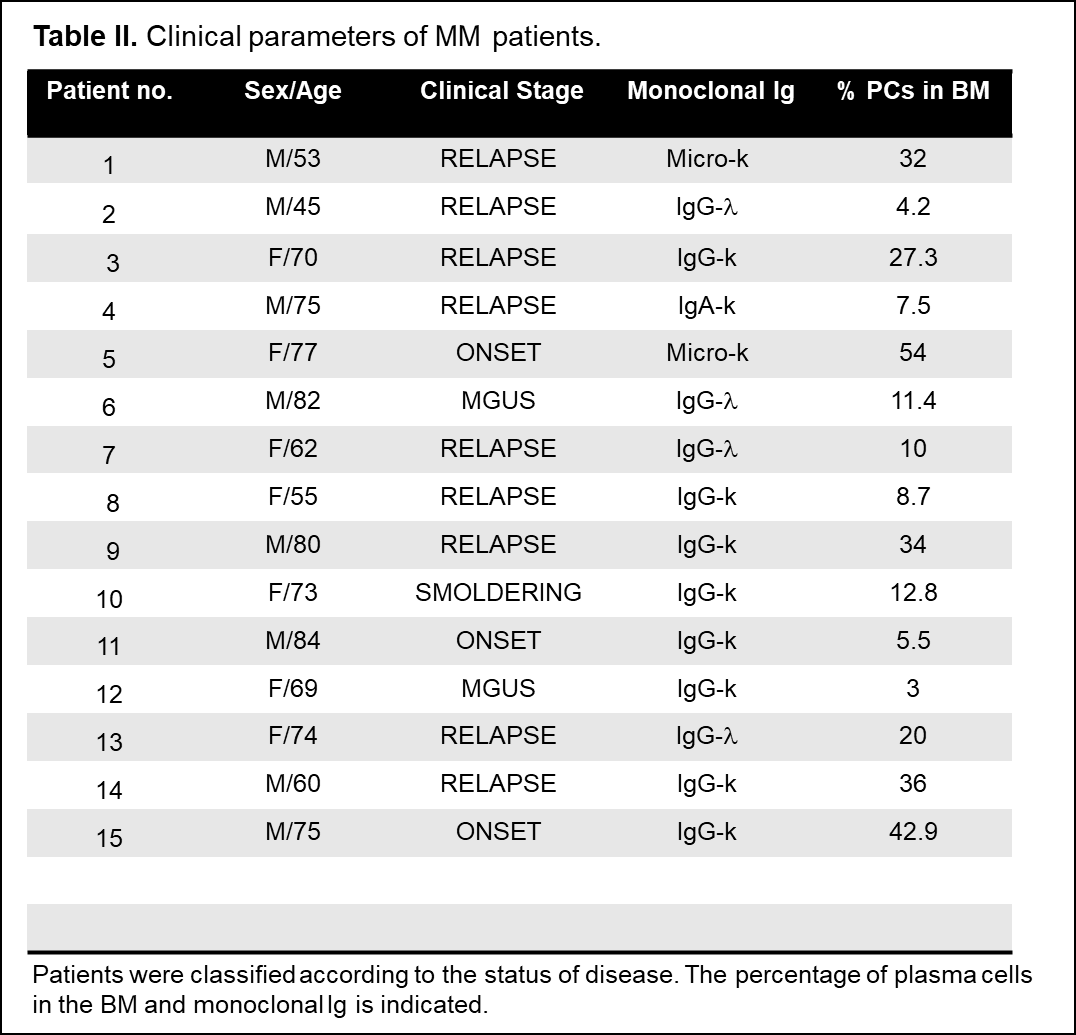

Supplement: Supplementary file 1 — Suppl. Figures - Tables + Legends [file 41419_2025_8196_MOESM1_ESM.docx]
