# Supplementary material for: Targeting RNA polymerase I to boost natural killer cell anticancer activity in multiple myeloma
Source: Cell Death Dis. 2025 Nov 28;16(1):865. doi: 10.1038/s41419-025-08196-6 (PMC12663147; doi:10.1038/s41419-025-08196-6)

Suppl. Figure 10 E, F

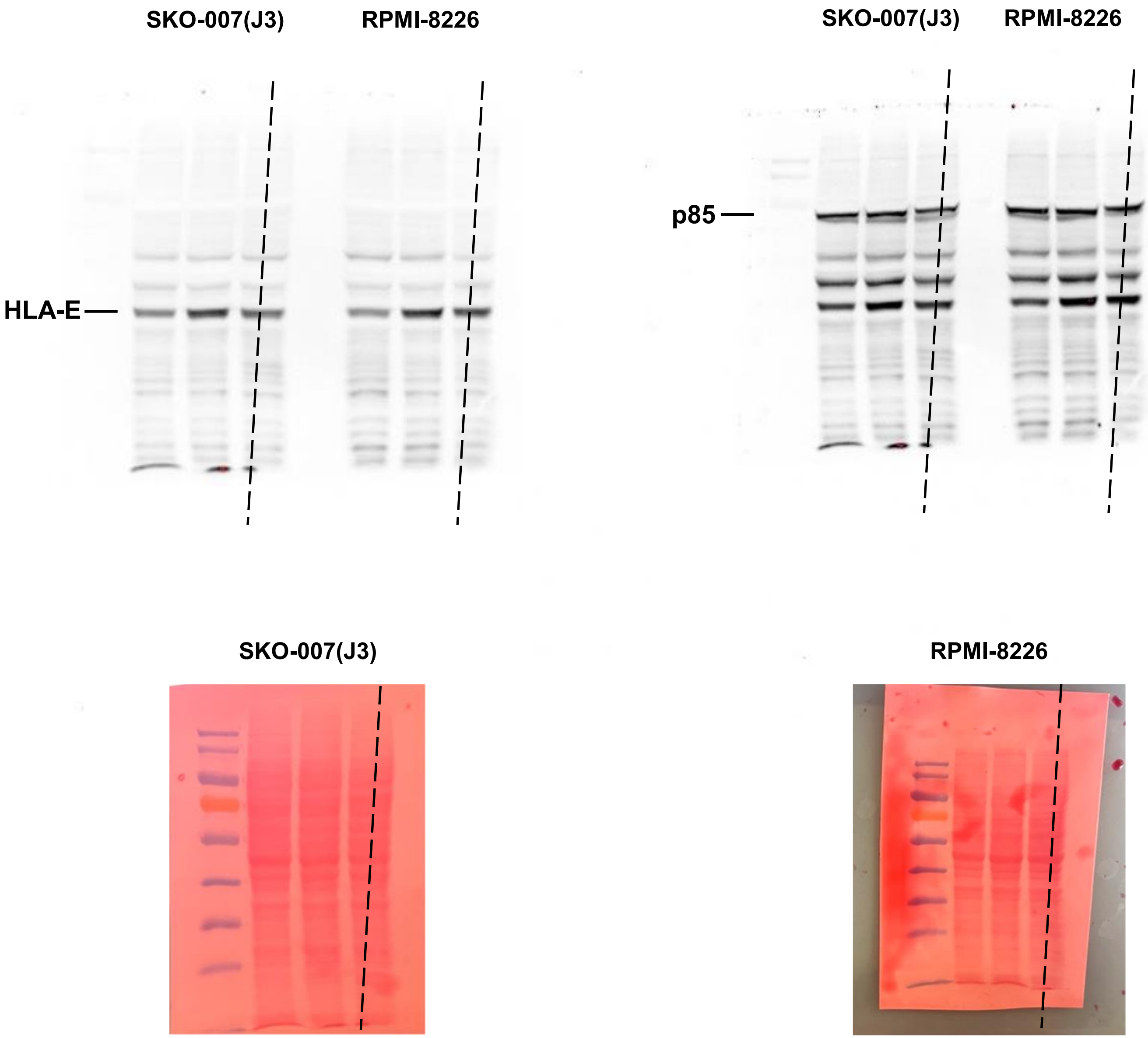

Suppl. Figure 10 G

SKO-007(J3)

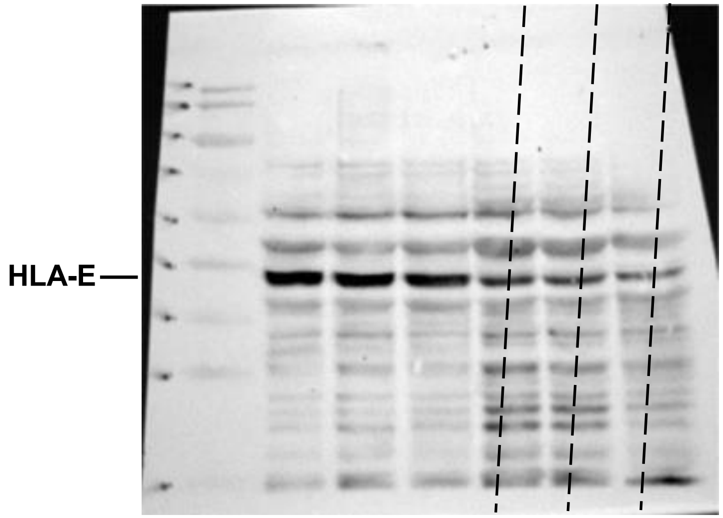

SKO-007(J3)

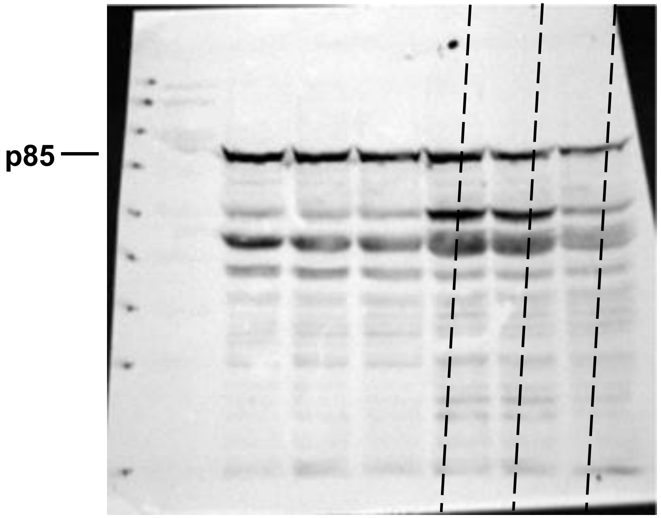

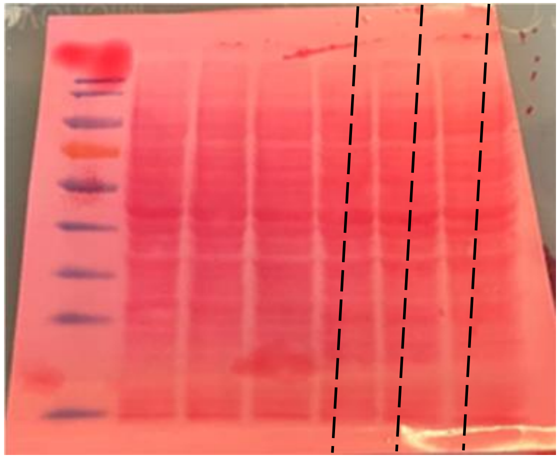

Suppl. Figure 10 I

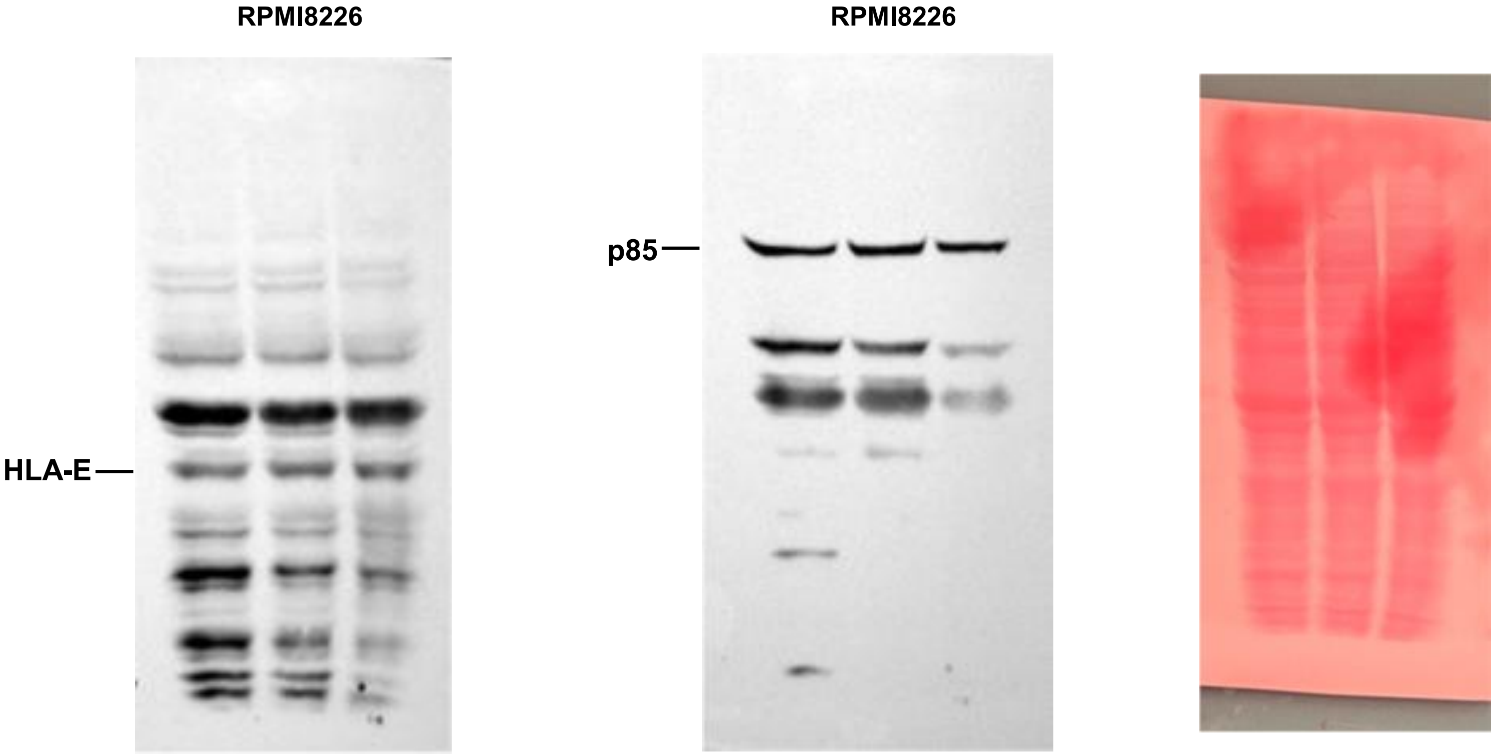

Figure 6B

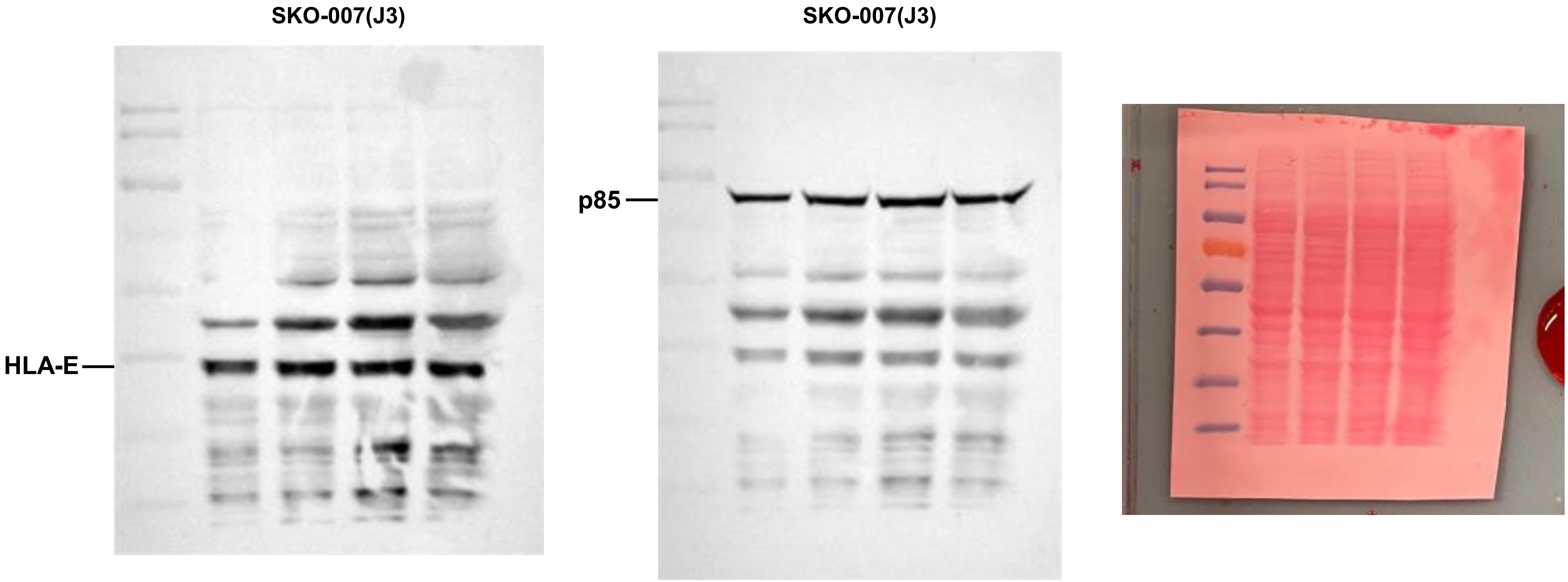

Figure 6D

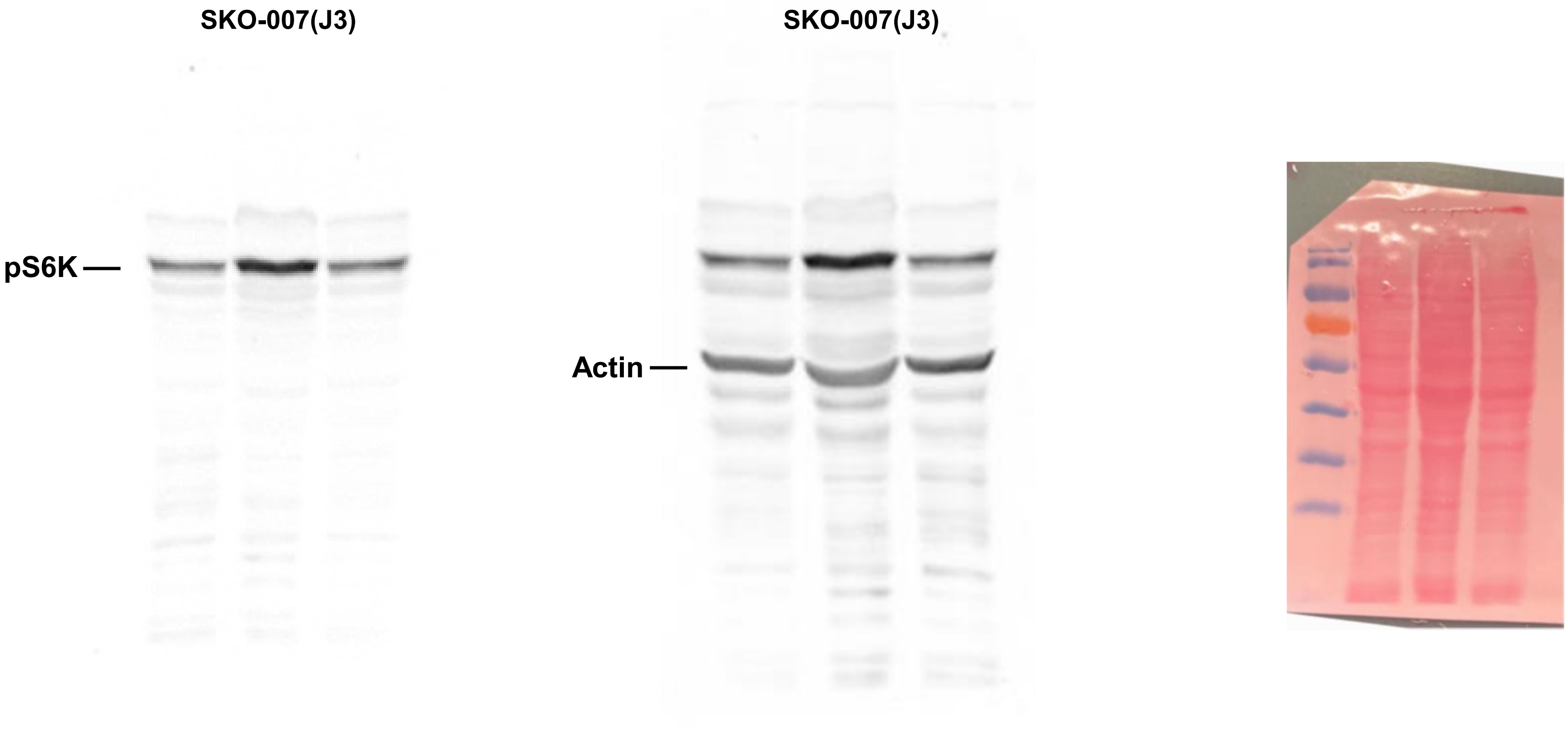

Suppl. Figure 12B

RPMI8226

HLA-E

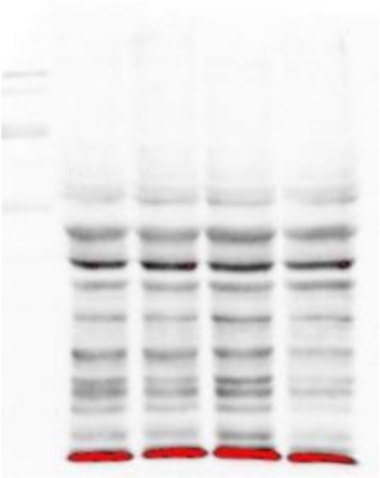

RPMI8226

p85

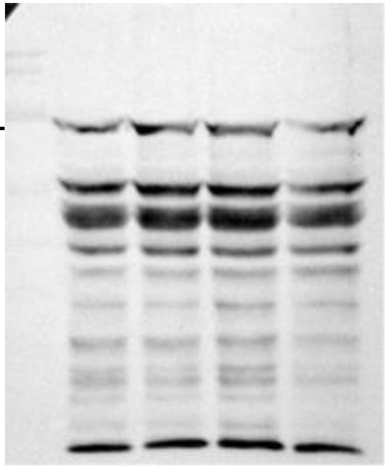

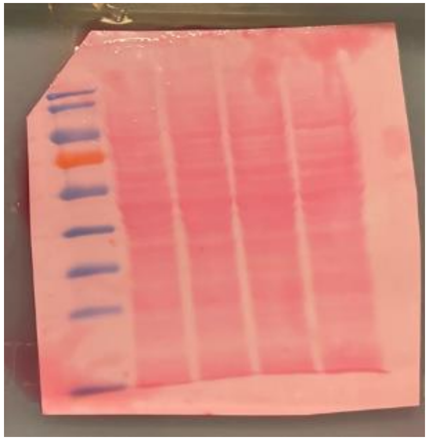

Suppl. Figure 13 A-B

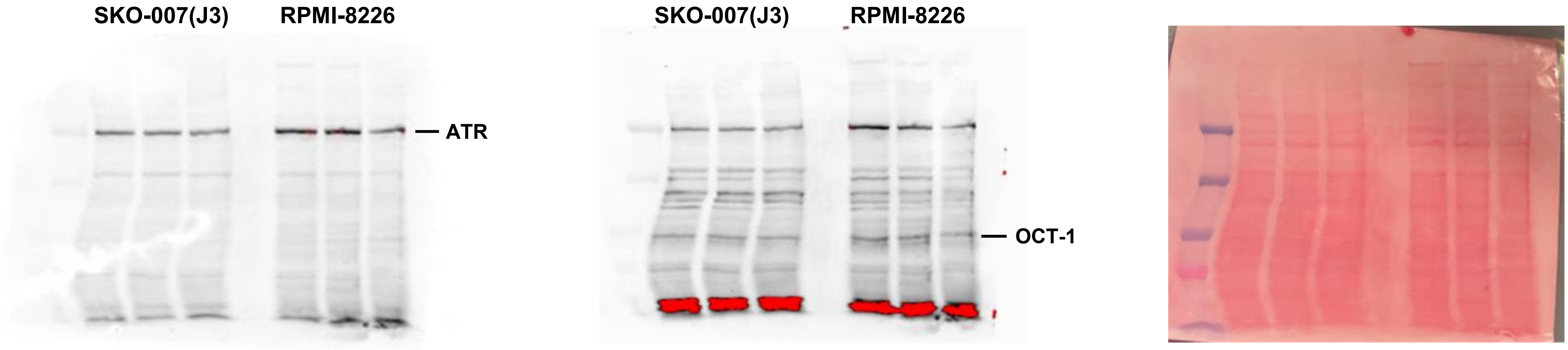

Suppl. Figure 13 C-D

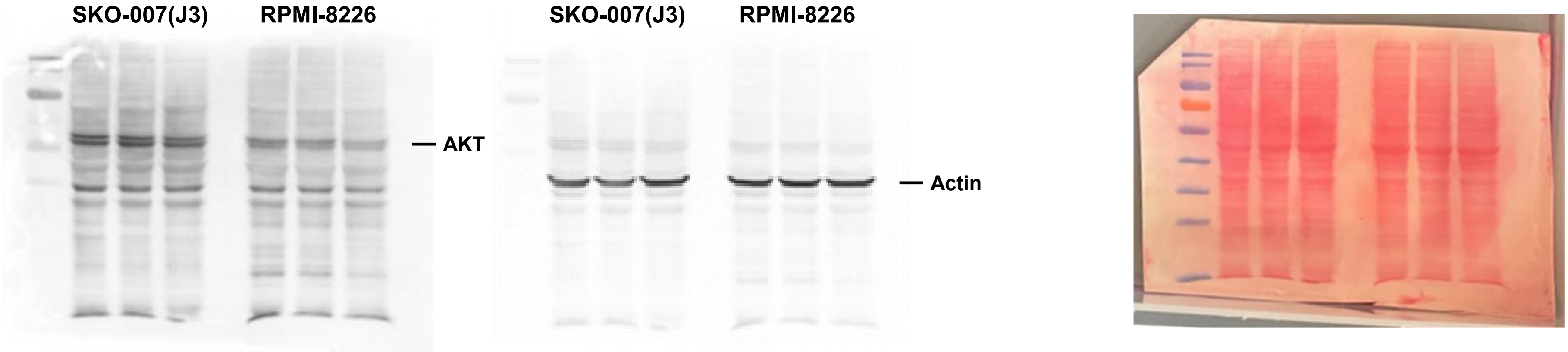

Suppl. Figure 13 E-F

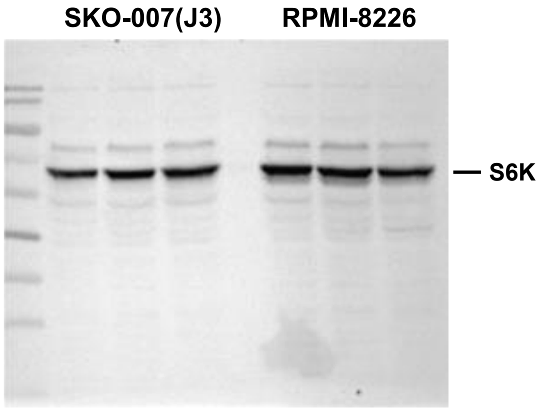

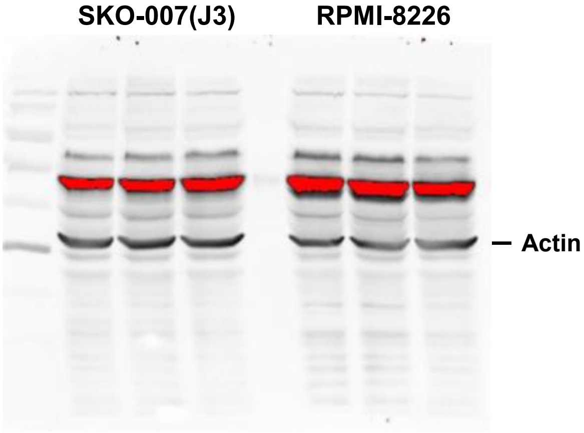

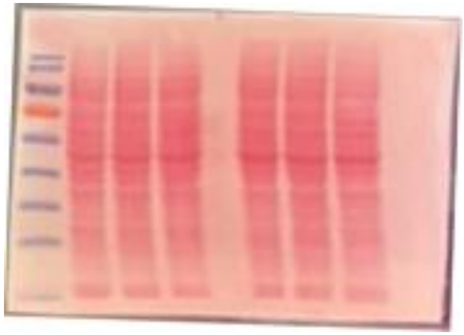

Suppl. Figure 14C

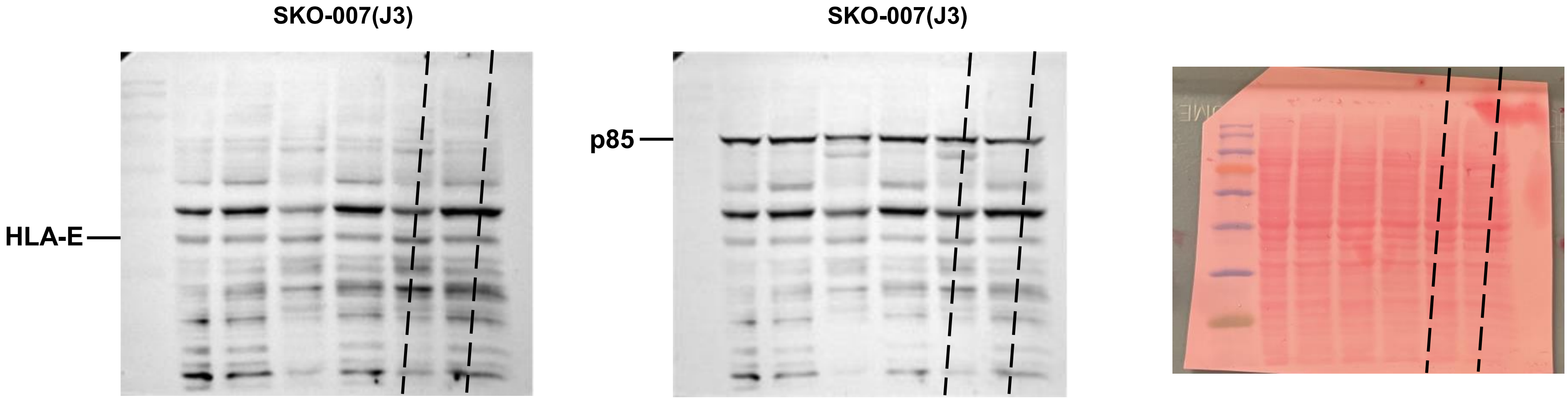

Suppl. Figure 14D

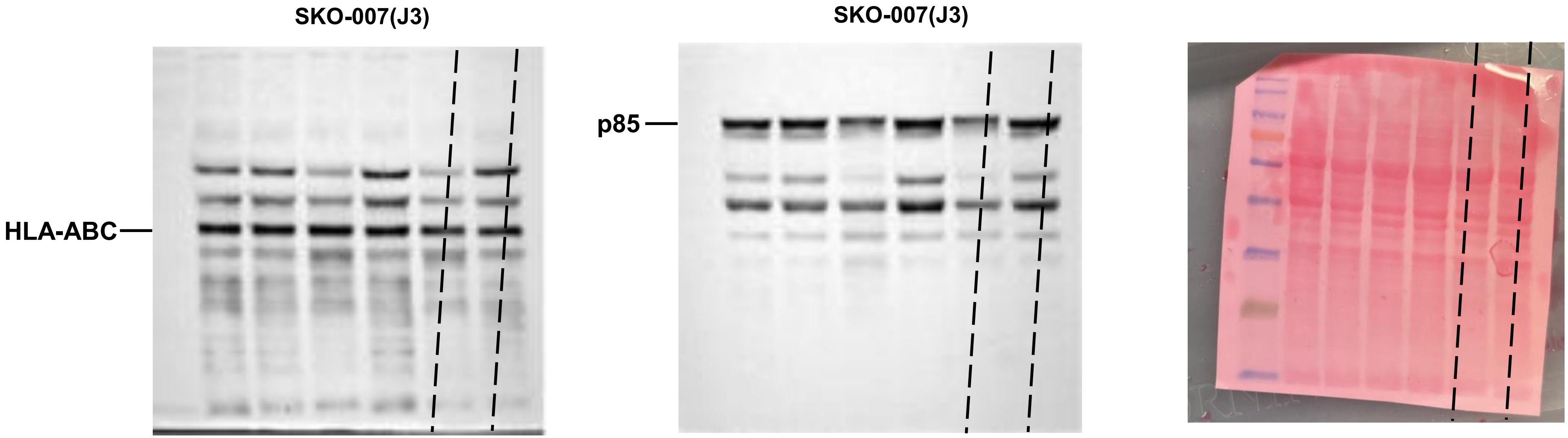

Supplement: Supplementary file 2 — Gels Uncropped [file 41419_2025_8196_MOESM2_ESM.pdf]
